# Identification of an Immune-Related Gene Signature to Improve Prognosis Prediction in Colorectal Cancer Patients

**DOI:** 10.3389/fgene.2020.607009

**Published:** 2020-12-04

**Authors:** Siqi Dai, Shuang Xu, Yao Ye, Kefeng Ding

**Affiliations:** ^1^Department of Colorectal Surgery and Oncology, Key Laboratory of Cancer Prevention and Intervention, Ministry of Education, The Second Affiliated Hospital, Zhejiang University School of Medicine, Hangzhou, China; ^2^Zhejiang University Cancer Center, Hangzhou, China; ^3^Department of Clinical Laboratory, Peking University People’s Hospital, Beijing, China

**Keywords:** colorectal cancer, immunity, prediction model, gene signature, prognosis

## Abstract

**Background:**

Despite recent advance in immune therapy, great heterogeneity exists in the outcomes of colorectal cancer (CRC) patients. In this study, we aimed to analyze the immune-related gene (IRG) expression profiles from three independent public databases and develop an effective signature to forecast patient’s prognosis.

**Methods:**

IRGs were collected from the ImmPort database. The CRC dataset from The Cancer Genome Atlas (TCGA) database was used to identify a prognostic gene signature, which was verified in another two CRC datasets from the Gene Expression Omnibus (GEO). Gene function enrichment analysis was conducted. A prognostic nomogram was built incorporating the IRG signature with clinical risk factors.

**Results:**

The three datasets had 487, 579, and 224 patients, respectively. A prognostic six-gene-signature (CCL22, LIMK1, MAPKAPK3, FLOT1, GPRC5B, and IL20RB) was developed through feature selection that showed good differentiation between the low- and high-risk groups in the training set (*p* < 0.001), which was later confirmed in the two validation groups (log-rank *p* < 0.05). The signature outperformed tumor TNM staging for survival prediction. GO and KEGG functional annotation analysis suggested that the signature was significantly enriched in metabolic processes and regulation of immunity (*p* < 0.05). When combined with clinical risk factors, the model showed robust prediction capability.

**Conclusion:**

The immune-related six-gene signature is a reliable prognostic indicator for CRC patients and could provide insight for personalized cancer management.

## Introduction

Colorectal cancer (CRC) is the third most common malignant tumor worldwide and ranks second in tumor-related deaths (Bray et al., 2018). In China, CRC is third highest in annual incidence and is the fifth leading cause of cancer-related deaths (Chen W. Q. et al., 2018). For operable disease, resection offers the best chance of long-term survival and potential cure (Adam et al., 2004). For inoperable patients, chemotherapy (mostly 5-fluorouracil- or oxaliplatin-based) and target therapy (epidermal growth factor receptor or vascular endothelial growth factor-targeted) have been the standard of care (Xie et al., 2020). However, despite recent advancements in chemo-regimens and clinical management, the overall survival of CRC remains unsatisfactory: The 5 years overall survival is just over 50% (Frampton and Houlston, 2017). More disconcertingly, there is great heterogeneity in individuals not only regarding tumor development but also in the response to uniform treatment: In those receiving surgeries, while some enjoyed disease-free survival, many suffered from tumor recurrence (Stelzner et al., 2019). The same is seen during non-surgical management, where tumor reactions vary: Less than 60% of patients had objective treatment response (Okuno et al., 2017), and adverse tumor response remains a strong predictor for unfavorable survival (Saskia et al., 2019). Thus, identifying reliable biomarkers for prediction of tumor behavior and outcome will benefit personalized modification in clinical management.

Recently, immune checkpoint blockade therapies that provide revolutionary treatments in multiple solid tumors (melanoma, non-small cell lung cancer, head-and-neck squamous cancer, colorectal cancer, etc.) have brought the community’s attention to tumor-related immunology (Chen Q. et al., 2018; Pagni et al., 2019). It is increasingly recognized that immune conditions play a decisive role in the genesis and progression of malignant tumors. The host’s immune dysfunction significantly impairs the body’s anti-tumor surveillance, along with cells’ immune-avoiding mechanisms acquired from the accumulation of gene mutations, marking a vital step toward tumor development (Croci et al., 2007; Shi et al., 2015; García-Albéniz et al., 2019). The most widely recognized prognostic biomarkers for immune therapy are programmed death ligand (PD-1), tumor mutation burden (TMB), and microsatellite instability/mismatch repair deficiency (dMMR) (Duffy and Crown, 2019). However, throughout the published research, these solitary biomarkers only showed moderate stratification efficacy (Snyder et al., 2014; Patel and Kurzrock, 2015; Van Allen et al., 2015; Mansfield et al., 2016), more so in CRC (Ciardiello et al., 2019), and a universal immune-related gene (IRG) panel as prognostic signature in CRC has not been scored.

In the last decade, several limited-scale studies have attempted to develop a predictive gene signature to stratify high-risk populations using high-throughput technology (Ito et al., 2013; Abdul Aziz et al., 2016; Li et al., 2020). However, most suffer from overfitting due to insufficient sample pools, and external validation is rarely presented. In addition, differences among high-throughput protocols often lead to inconsistency in expression values among studies, presenting a challenge to comprehensive meta-data analysis. From this perspective, the publicly available large-scale genomic databases provide sufficient samples, comparable gene expression at the probe level, and solid follow-up information, and thus have been recognized as ideal platforms for gene signature construction and validation.

In this study, we aimed to identify and validate an IRG signature to stratify CRC patients with significantly worse survival in two independent public databases. The signature was then incorporated with clinical risk factors to provide robust prediction efficacy regarding long-term survival.

## Materials and Methods

**FIGURE 1:**
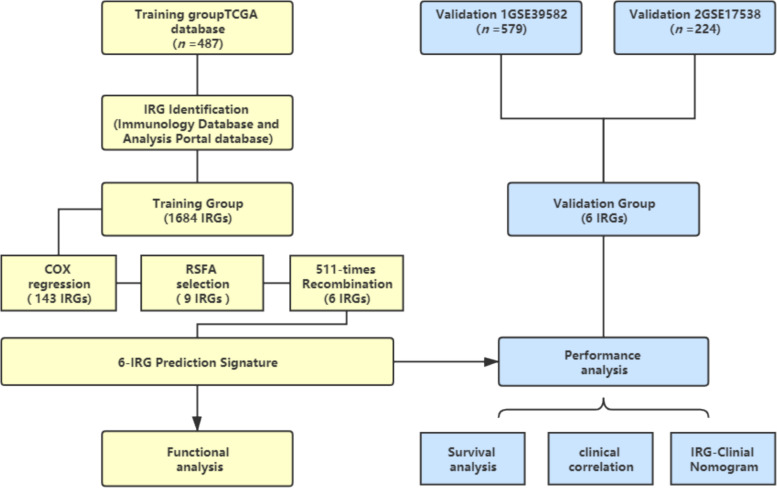
Schematic of this study. Survival and relevant clinical information, along with IRG expression data, were acquired from an online database. The TCGA database (*n* = 487) was used as the training group. An IRG prediction panel was developed via the aforementioned procedures and was tested for its function via GO and KEGG analyses. The model’s discrimination was assessed and validated in GSE39582 (*n* = 578) and GSE17538 (*n* = 224). TCGA, The Cancer Genome Atlas; IRG, Immune-related gene; RSFA, random survival forest algorithm.

A schematic of this study is shown in Figure 1.

### Gene Expression Data Acquisition of CRC Patients

Two sets of colorectal cancer patients with clinical information, including survival status and survival time, were retrospectively enrolled from the publicly available The Cancer Genome Atlas (TCGA)^1^ ^,2^ and Gene Expression Omnibus (GEO, GSE39582, GSE17538) data pool as training and external validation sets, respectively. CRCs with clinical variables and genes were comprehensively extracted using the following procedures. Samples with complete survival information were selected, and genes with missing expression values in >20% samples were removed.

### Selection of Immune-Related Genes

To filter genes that actively participate in immune activity, a third comprehensive data set of immune genes was acquired from the Immunology Database and Analysis Portal database (ImmPort)^3^. After cross-referencing with the ImmPort database, a pack of 2,112 immune-related genes (IRGs) was obtained. As some genes showing no expression value in the above three gene expression profiles, a panel of 1,684 expressing IRGs was further selected for survival analysis (Supplementary Table S1).

### Development of Prognostic Gene Models in the Training Set

Univariate Cox regression was performed for each gene regarding survival status to screen for prognostic immune-related genes. For those showing statistical significance (*p* < 0.05) in Cox regression, the random survival forest algorithm (RSFA) was adopted for dimensional reduction. Further, the risk scores of the prognostic models were determined as follows:

$$\mathrm{Risk}\,\mathrm{Score}=\Sigma_{i=1}^{N}\,\left(E\,x\,p\,r\,e\,s\,s\,i\,o\,n_{i}\times c\,o\,e\,f\,f\,i\,c\,i\,e\,n\,t_{i}\right)$$

where *N* is the number of genes, *Expression* is the gene expression value, and *coefficient* is the gene coefficient value in the Cox regression analysis, while the median risk score was utilized to group the patients as Low-Risk and High-Risk population.

To rule out overfitting, we constructed full-scale combinations of genes yielded in the RSFA. Time-dependent receiver operating characteristic (time-ROC) analysis was used to test the performance. The C-index, which by value equals the area under curve (AUC), was used to evaluate the concordance between the prediction model and reality. The combination with the highest C-index was designated as the optimal prediction model, which was subsequently verified for performance in internal and external verification (GSE39582 and GSE17538).

### Construction and Assessment of a Novel Nomogram Incorporating IRGs and Clinical Factors

We then sought to develop a comprehensive model with clinical features. Via univariate and multivariate Cox regression analyses, independent clinical risk factors (*p* < 0.05) were incorporated into the IRG panel. Based on these, a comprehensive prediction nomogram was formulated. Subsequently, we used a time-ROC test at different time points to test its performance in the training and validation groups. In addition, the nomogram’s prediction bias was evaluated.

### Statistical and Bioinformatics Analysis

Statistical analysis was performed with R Software (version 3.6.2), while pROC, TimeROC, randomForestSRC, and survival packages were utilized. Data distribution was validated using the Kolmogorov-Smirnov test. The statistical significance of continuous variables between the training and validation sets was measured using Student’s *t*-test. Chi-square or rank-sum tests were performed for layered variables. Kaplan-Meier analysis was used to assess the high- and low-risk groups. A *Z*-test was adopted for statistical differences between ROC curves. The co-expressing genes (CEGs) of the selected IRGs were then screened using co-expression network analysis by Pearson test (| Pearson coefficient| 0.6, *p* < 0.001). To explore the function of the selected co-expressing genes, gene enrichment, namely gene ontology (GO) analysis and KEGG analysis, was analyzed by ClueGO (Bindea et al., 2009), a Cytoscape plug-in to perform GO and KEGG analysis.

## Results

### Identifying the Prognostic Signature in the Training Set

Following the aforementioned criteria, three datasets with a total of 1,290 patients with CRC were enrolled: one training set (TCGA, *n* = 487) and two validation sets (GSE39582, *n* = 579; GSE17538, *n* = 224). The clinical characteristics are presented in Table 1. The median age of the patients in TCGA was 68 years. At the time of enrollment, most patients were alive (77.8% in the training and 66.6% and 59.8% in the two independent validation sets, respectively), and the median surveillance times were 699, 1,582, and 1,401 days in TCGA, GSE39582, and GSE17538, respectively. Most patients did not have lymph node involvement (stages I–II).

**TABLE 1:** Clinical characteristics of the CRC patients.

**Characteristic**	**TCGA (*n* = 487)**	**GSE39582 (*n* = 579)**	**GSE17538 (*n* = 224)**
**Age**			
Unknown		1	
≤68	243	311	128
>68	244	267	96
**Gender**			
Female	229	260	106
Male	258	319	118
**Survival status**			
Living	379	385	134
Dead	108	194	90
**Pathological M**			
Unknown	63	22	
M0	355	496	
M1	69	61	
**Pathological N**			
Unknown		26	
N0	291	311	
N1	111	136	
N2	85	106	
**Pathological T**			
Unknown	1	24	
T1	11	12	
T2	83	48	
T3	334	376	
T4	58	119	
**Tumor stage**			
Unknown	12	4	0
Stage I	80	37	27
Stage II	193	269	70
Stage III	133	209	75
Stage IV	69	60	52

*The clinical characteristics of the training group (TCGA database, n = 487) and the two test groups (GSE39582, n = 578; GSE17538, n = 224). The median age of the training group was 68 years. By the end of surveillance, most patients were alive (77.8, 66.6, and 59.82%). CRC, Colorectal Cancer.*

**FIGURE 2:**
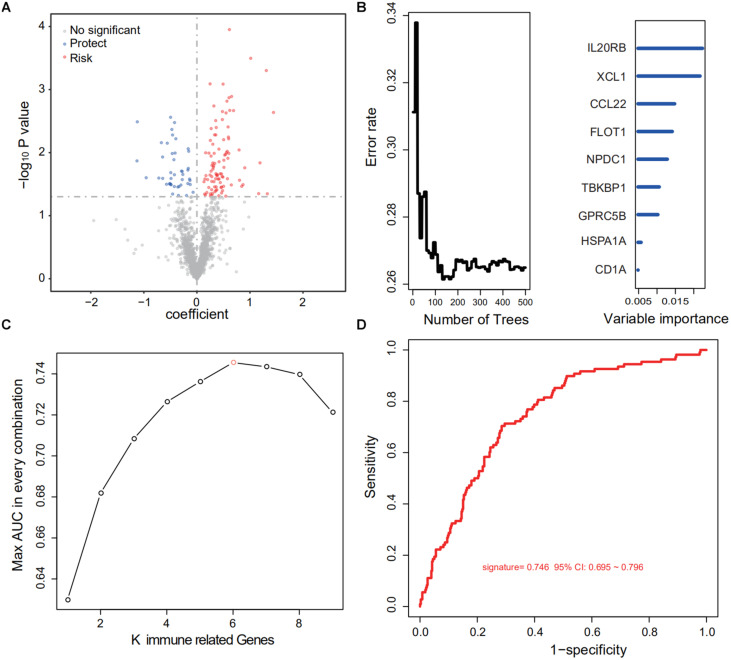
Identification of a six-IRG panel for prognosis prediction. **(A)** Univariate Cox regression of 1,684 IRGs regarding patient survival. IRGs with statistical significance (*n* = 143) were marked blue if protective or red if risk factors. **(B)** RSFA was used to select highly survival-correlated IRGs. Nine genes were enrolled. **(C)** Full-scale recombination of the nine IRGs was conducted to rule out overfitting (combinations, *n* = 511). A six-gene panel with an AUC of 0.746 was selected as the optimal prediction panel. **(D)** ROC curve of the designated IRG panel.

After cross-comparison with the ImmPort database, 2,112 immune-related genes were selected. Then, repeated, not, or inconsistently expressed genes were excluded, leaving 1,684 genes as candidates. For each gene, univariate COX regression was performed, and 143 IRGs suggested significant protective or risk effects (Figure 2A and Supplementary Table S1). Via RSFA, nine immune-related genes were identified as independent prognostic predictors (Figure 2B).

Next, to explore the optimal IRG signature and preclude overfitting, we formed a panel of full-size combinations of these nine genes (2^9^ − 1 = 511 combinations, Supplementary Table S2). Using the previously discussed risk formula, 511 candidate predictive signatures were calculated. The performance of each signature was verified via a time-ROC curve. The AUC values of each were rated. A combination of the following genes: CCL22, LIMK1, MAPKAPK3, FLOT1, GPRC5B, IL20RB was screened out with the highest prediction precision (AUC = 0.746; Figures 2C,D). Each IRG’s hazard ratio (HR) and *p*-value is listed in Table 2. Thus, the designated risk model was: Risk score = (−0.421 × expression value of CCL22) + (0.402 × expression value of LIMK1) + (−0.465 × expression value of MAPKAPK3) + (0.599 × expression value of FLOT1) + (0.613 × expression value of GPRC5B) + (0.596 × expression value of IL20RB).

**TABLE 2:** Survival analysis of the IRG in the prognostic signature.

**Symbol**	**Ensemble ID**	**HR**	**Right**	**Left**	**COX *P-value***
CCL22	ENSG00000102962	0.66	0.5	0.87	<0.001***
LIMK1	ENSG00000106683	1.5	1.05	2.13	0.03*
MAPKAPK3	ENSG00000114738	0.63	0.44	0.9	<0.01**
FLOT1	ENSG00000137312	1.82	1.19	2.79	<0.01**
GPRC5B	ENSG00000167191	1.85	1.35	2.52	<0.001***
IL20RB	ENSG00000174564	1.82	1.35	2.43	<0.001***

The final prediction model included six immune-related genes. The genes’ hazard ratios (HRs), 95% confidence intervals (CIs), and corresponding p-values are presented. IRG, Immune-related gene; HR, Hazard ratio. * P < 0.05; ** P < 0.01; *** P < 0.001.

### The Performance of the Signature in Predicting Overall Survival

**FIGURE 3:**
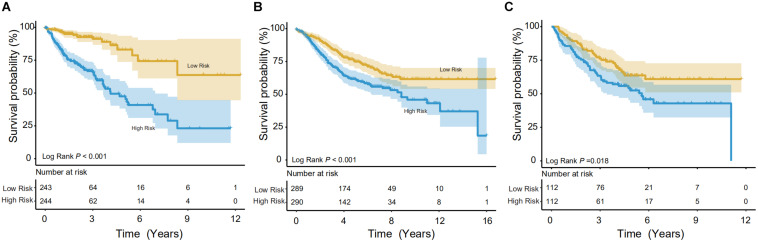
Survival analysis in the training and validation datasets. The Kaplan-Meier (KM) test was performed on the training and validation data to verify survival between high- and low-risk populations. **(A)** In the training group (TCGA), the model showed good discrimination (*p* < 0.001). **(B,C)** In both validation groups (GSE39582 and GSE17538, respectively), the low-risk population showed significantly better survival.

Using the IRG model, a risk score was calculated for individuals. In the training set, the Kaplan-Meier (KM) test was performed to verify the survival difference between the high- (*n* = 243) and low-risk (*n* = 244) populations, divided by median risk-score-value. The method was consistent with other studies (Song et al., 2019; Wang et al., 2020a). As shown in Figure 3A, significant longevity was observed in low-risk patients in the training set (log-rank *p* < 0.001). The median survival time was 8.46 years in low-risk patients vs. 4.12 years in high-risk populations. To explore this in other independent databases, the same methodology was then adopted for the GSE39582 validation set with a relatively larger sample pool (Figure 3B), and the model showed significant differentiation capability (median survival time: 8.83 years in the low- vs. 4.67 years in the high-risk group, *n* = 579, log-rank *p* < 0.001). Finally, the survival prediction performance was tested in the GSE17538 dataset, and it could also distinguish the CRCs into high- or low-risk groups (5 years survival: 45.90 vs. 63.68% (*n* = 224), log-rank *p* < 0.05, Figure 3C).

**FIGURE 4:**
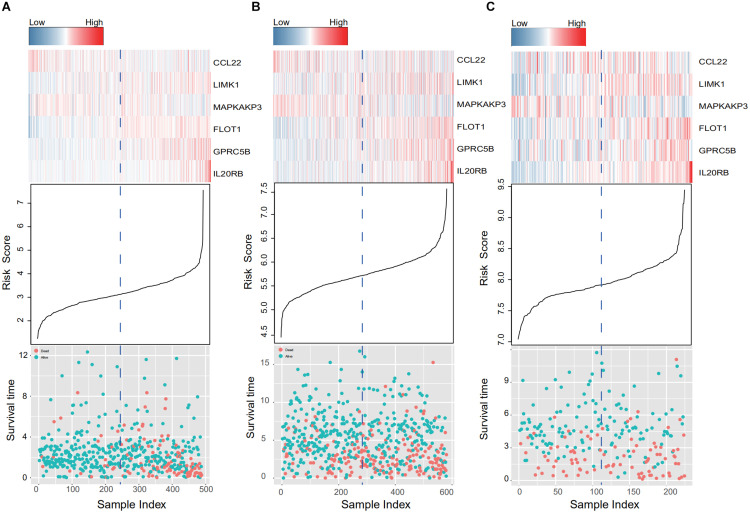
IRG expression and survival information in the training and validation data sets. **(A–C)** Present the TCGA, GSE39582, and GSE17538 database results, respectively. Patients were queued according to their risk scores. Clusters in gene expression level and survival information in the training and validation groups are given. In high-risk populations, genes with adverse prognostic effects, namely LIMK1, FLOT1, GPRC5B, and IL20RB, showed consistent elevation in expression.

When the patients in the training and two validation datasets are queued by risk score, clusters in gene expression level and survival information can be observed in Figure 4. In the training dataset (Figure 4A), patients with shorter survival times had higher risk scores, and genes with adverse prognostic effects, namely LIMK1, FLOT1, GPRC5B, and IL20RB, showed consistent elevation in expression in high-risk populations (Figure 4A). In addition, consistent trends were confirmed in the two external validation sets (GSE39582 and GSE17538, Figures 4B,C, respectively).

### The Relationship Between the Signature and Clinical Characteristics

We further explored the potential relationship between gene signature and clinical characteristics in TCGA and GEO databases (Table 3). Neither patient age (stratified by 68 years) nor gender showed a correlation with gene signature via Pearson’s χ^2^-test. Tumors’ TNM staging was significantly advanced in the high-risk population in TCGA and GSE39582 (*p* < 0.001). In univariate Cox regression analysis, old age (>68 years), more advanced tumor stage (stage III and IV), and immune-related gene signature showed statistically significant effects, confirming them as independent adverse predictors via the following multivariate COX regression. In all three groups, the gene signature suggested great predictive potential regarding the clinical outcomes of CRC patients (High- vs. Low-risk, HR_training–TCGA_ = 4.56, 95% CI 2.81–7.40, *p* < 0.001, *n* = 487; HR_test__1__–__ GSE__39582_ = 1.55, 95% CI 1.16–2.07, *p* < 0.001, *n* = 579; HR_test__2__–__ GSE__17538_ = 1.72, 95% CI 1.10–2.69, *p* = 0.02, *n* = 224, Table 4).

**TABLE 3:** Association of the IRG signature with clinical characteristics in CRC patients.

**Variables**	**TCGA**		***P***	**GSE39582**		***P***	**GSE17538**		***P***
	**Low risk**	**High risk**		**Low risk**	**High risk**		**Low risk**	**High risk**	
**Age (years)**			0.3			0.46			0.58
Unknown				1					
≤68	115	128		150	161		62	66	
>68	128	116		138	129		51	45	
**Gender**			0.06			0.96			0.78
Female	125	104		129	131		55	51	
Male	118	140		160	159		58	60	
**Tumor stage**			<0.001***			<0.001***			0.27
Unknown	3	9		3	1				
Stage I	60	20		27	10		16	11	
Stage II	112	81		152	117		40	30	
Stage III	55	78		85	124		35	40	
Stage IV	13	56		22	38		22	30	
**Pathologic T**			<0.001***			<0.001***			
Unknown		1		5	18				
T1	9	2		7	5				
T2	59	24		31	17				
T3	157	177		198	178				
T4	18	40		47	72				
**Pathologic N**			<0.001***			<0.001***			
Unknown				7	25				
N0	177	114		184	127				
N1	48	63		62	74				
N2	18	67		36	64				
**Pathologic M**			<0.001***			<0.001***			
Unknown	26	37		3	19				
M0	204	151		263	233				
M1	13	56		23	38				

The links between clinical factors and the IRG signature were investigated. The patients were divided into low- and high-risk groups according to median risk factor. Age and sex were verified via χ^2^-test, while tumors’ T, N, and M stages were checked by the rank-sum test. Tumor pathological state and disease stage were significantly correlated with the IRG signature. IRG, Immune-related gene; CRC, Colorectal Cancer. *** P < 0.001.

**TABLE 4:** Cox regression analysis of the IRG signature with survival.

**Variables**		**Univariable analysis**				**Multivariable analysis**			
		**HR**	**95% CI of HR**		***P***	**HR**	**95% CI of HR**		***P***
			**lower**	**upper**			**lower**	**upper**	
**The TCGA group**									
Age	>68 vs.≤68	1.59	1.08	2.34	0.02*	1.78	1.2	2.64	<0.001***
Gender	Male vs. Female	1.15	0.79	1.69	0.47	0.91	0.61	1.34	0.62
Tumor stage	III, IV Vs. I, II	1.04	1.01	1.06	<0.001***	1.03	1	1.05	0.02*
Signature	High risk vs. Low risk	4.49	2.79	7.23	<0.001***	4.56	2.81	7.4	<0.001***
**The GSE39582 group**									
Age	>68 vs. ≤68	1.89	1.42	2.51	<0.001***	2.31	1.72	3.10	<0.001***
Gender	Male vs. Female	1.31	0.98	1.74	0.07	1.54	1.15	2.06	0.004**
Tumor stage	III, IV Vs. I, II	1.94	1.59	2.36	<0.001***	2.08	1.69	2.56	<0.001***
Signature	High risk vs. Low risk	1.7	1.27	2.26	<0.001***	1.55	1.16	2.07	0.003**
**The GSE17538 group**									
Age	>68 vs. ≤68	1.22	0.80	1.84	0.36	1.89	1.22	2.93	<0.001***
Gender	Male vs. Female	1.03	0.68	1.56	0.88	1.11	0.71	1.73	0.65
Tumor stage	III, IV vs. I, II	2.90	2.20	3.83	<0.001***	2.99	2.25	3.99	<0.001***
Signature	High risk vs. Low risk	1.65	1.08	2.51	0.02*	1.72	1.10	2.69	0.02*

In both the training and validation groups, patients’ disease stage, age, sex, and IRG signature were tested in univariate and multivariate COX regression, in which age, tumor stage, and IRG signature were independent prognostic factors. The hazard ratios, 95% CIs, and corresponding p-values are given. IRG, Immune-related gene. * P < 0.05; ** P < 0.01; *** P < 0.001.

### Comparing Predictive Performance of the Signature With Tumor Stage

The model’s performance was compared against tumor TNM staging in predicting clinical outcome. To this end, ROC curves in the TCGA and GSE39582 datasets were constructed to compare both models (Supplementary Figure S1). In the TCGA set (Supplementary Figure S1A), the C-index of the signature was 0.746 (95% CI: 0.695–0.796), higher than that of tumor stage (0.704, 95% CI: 0.651–0.758), while in the GSE39582 set (Supplementary Figure S1B), the C-index was 0.622 (95% CI: 0.574–0.670) vs. 0.609 (95% CI: 0.563–0.655), respectively. As indicated in Supplementary Figure S1, in both wings, the six-gene signature yielded superior accuracy against traditional staging.

### Development of a Predictive Gene-Clinical Nomogram for Clinical Outcome

**FIGURE 5:**
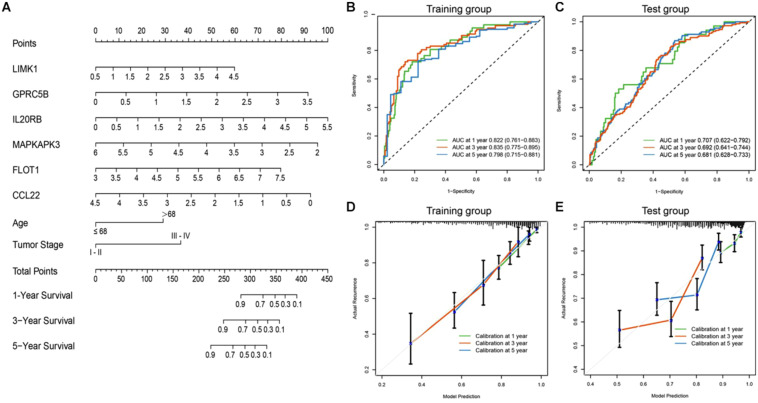
Nomogram incorporating clinical factors and IRG signatures. **(A)** A predictive nomogram was constructed using clinical risk factors and the designated IRG panel, via which patients’ 1, 3, and 5 years survival could be predicted. **(B,C)** Time-ROC curves of the nomogram in large (*n* > 400) independent database (TCGA and GSE39582). The AUC values were consistent at different time points. **(D,E)** Calibration curves were plotted to reveal the concordance between prediction and reality. In both the training and validation sets, the model showed good calibration.

To achieve comprehensive outcome prediction, the six-gene prediction model was combined with clinical independent risk factors, namely tumor stage and age, and transformed into a predictive nomogram (Figure 5A) to provide a straightforward estimation of survival at 1, 3, and 5-year intervals. For instance, old-aged (>68 years) advanced-staged (stages III–IV) patients with a gene signature value of 4 would have a total risk score of roughly 60, and the odds of survival would be 80, 55, and 35%. Via time-ROC (Figure 5B), the AUC values in the training group at 1, 3, and 5 years were 0.822 (95% CI: 0.761–0.883), 0.835 (95% CI: 0.775–0.895), and 0.798 (95% CI: 0.715–0.881), respectively. An external group (GSE39582) was used for model validation and yielded overall comparable precision: The AUCs at 1, 3, and 5 years were 0.707 (95% CI: 0.622–0.792), 0.692 (95% CI: 0.641–0.744), and 0.681 (95% CI: 0.628–0.733) (Figure 5C). It could be judged from the nomogram that the six-gene signature was the most prominent predictor of patient survival, and the performance of the gene-clinical nomogram was consistent over various time points.

To assess how this nomogram mimics a real situation, calibration curves using a 1,000-time bootstrap test were plotted. As shown in Figure 5D, in the training set, the nomogram presents good agreement between prediction and real situation. Furthermore, in the external calibration (Figure 5E), the calibration curve showed a minor wobble, but still in the near proximity of the 45-degree-dashed–dashed line. These results suggest that our nomogram closely predicts real-life situations, and via internal and external validation in two independent large-scale databases, the nomogram showed great utility.

### Exploring the Function of the Signature

**FIGURE 6:**
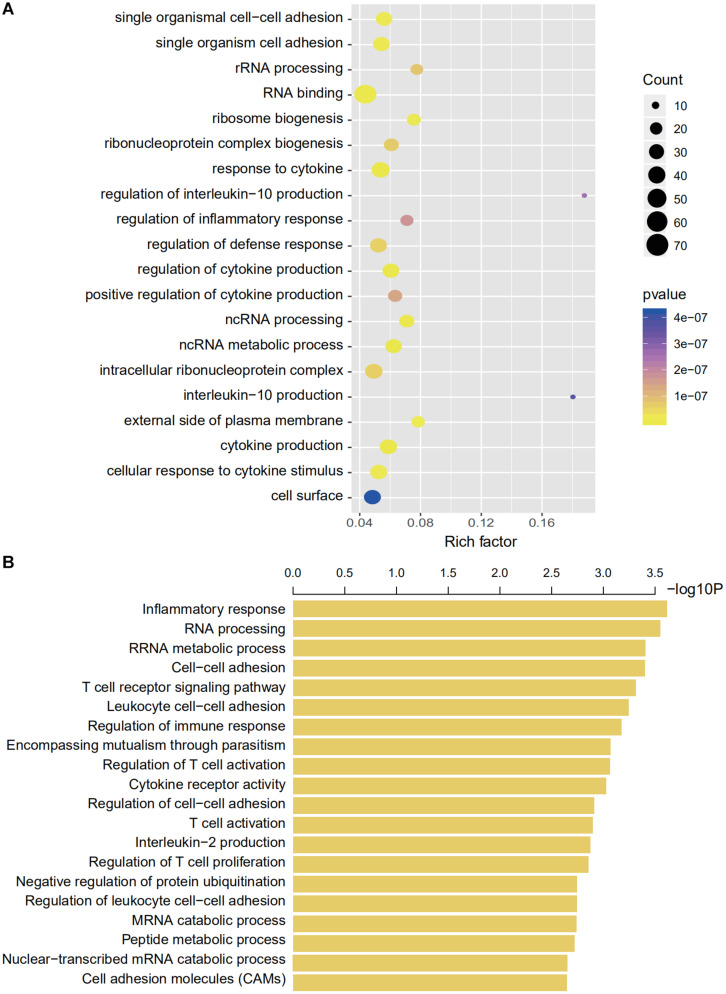
Gene enrichment and functional analyses using co-expressing genes. **(A)** Gene enrichment analyses using co-expressing genes. The colors of the dots represent the statistical correlation with the selected IRGs. The size of the dots indicates the number of associated genes. **(B)** Functional enrichment analyses of co-expressing genes.

We then explored the potential genetic functions of the IRG panel. In the TCGA dataset (*n* = 487), the co-expressed relationships of the six genes with the protein-coding genes were computed using Pearson correlation test. The expressions of 446 protein-coding genes were highly associated with at least one of the genes in the signature (|Pearson correlation coefficient| > 0.60, *p* < 0.001). Next, we performed GO and KEGG pathway function enrichment analysis for these co-expressing protein-coding genes (ClueGo plug-in, Cytoscape). Clusters including 104 GO terms and 3 KEGG functionally pathways were identified (Supplementary Table S3, *p* < 0.05). The results of these analyses implied that the signature might be involved in tumorigenesis by interacting with protein-coding genes that affect important biological processes such as regulation of immune and inflammatory responses and metabolic processes (Figure 6B).

## Discussion

Recent advances in immune checkpoint blockade therapy warrant further understanding of immune gene variation, and there is an imminent need for robust prognostic biomarkers to guide selective management strategies. In this study, we used three independent, large-scale international genome databases for the exploration and validation of a prognostic IRG panel. We performed dimension-reduction of acquired IRG data and ruled out overfitting, which is commonly seen in other studies. We developed a full-scale recombination of nine figures (511 combinations). Finally, the most accurate six-gene prediction signature was selected. The signature alone showed improved prognostic performance compared to tumor stage (C-index 0.746 and 0.622, against 0.704 and 0.609 with tumor stage in the training and validation groups, respectively).

There have been a few recently published studies using IRG signatures to predict prognosis in CRC patients. However, not all of these were conducted with a reasonable sample size, and only moderate performance was achieved. Zuo et al. (2019) developed a six-gene signature model to forecast patient prognosis without external validation. In their study, gene selection was based on multivariate regression, and no recombination was performed to rule out overfitting. Indeed, the AUC was only 0.711 and 0.683 for the 3 and 5 years survival, respectively, and inconsistency was seen in the subgroup analysis. Bai et al. (2020) also reported a 14-IRG panel using the TCGA cohort with the absence of any external validation. In addition, they conducted GO and KEGG analyses not with CEG of the selected IRG, but with the CEG of the whole set of 676 IRGs. From this perspective, the accuracy of the analysis could be biased. Our proposed link between IRG signature and disease characteristics was further confirmed by Wang et al. earlier this year (Wang et al., 2020b). Regrettably, none of the abovementioned studies incorporated IRG signatures with clinical risk factors for outcome predictions, so their clinical utilities were largely limited. In contrast, the present study enrolled a large number of patients. The 1,290 CRC patients’ IRG sequencing data and clinical characteristics were downloaded from three independent international databases that include patients of various regions and ethnicities, which adds to the utility and credibility of the IRGs signature.

The included immune-related genes for signature were CCL22, LIMK1, MAPKAPK3, FLOT1, GPRC5B, and IL20RB. Through a literature search, CCL22 was identified as an upstream regulator of the PI3K/AKT pathway. Secreted by M2 macrophages, CCL22 regulates the epithelial-mesenchymal transition (EMT) of CRC cells and promotes tumor resistance to chemotherapy (Wang et al., 2019; Wei et al., 2019). FLOT1 also induces EMT and alters the cell cycle by modulating the Erk/Akt signaling axis (Zhang L. et al., 2019). In addition, the prognostic value of IL20RB has been actively discussed in multiple tumors including glaucoma, anal cancer, and lung adenocarcinoma (Wirtz and Keller, 2016; Jeannot et al., 2018; Zhang M. et al., 2019). Moreover, MAPKAPK3 is a member of stress-responsive kinases that induce autophagy in terms of stress (inflammation, infection, and starvation) and thus determines cell fate (Wei et al., 2015; Menon et al., 2017).

The clinical application of gene mutations as prognostic biomarkers is largely limited thus far. The Ras family (KRas and NRas) has been recognized as an indicator of epithelial growth factor receptor status (Amodio et al., 2020). The BRAF V600E mutation was identified as an indication for anti-vascular endothelial growth factor (VEGF) treatment (Apte et al., 2019). Additionally, microsatellite instability has gained increasing attention with the introduction of immune therapy (Hause et al., 2016). However, using individual gene status as prognostic biomarkers did not yield very reliable efficiency (Sjoquist et al., 2018). Independent studies of single gene mutations tend to result in conflicting conclusions. The selected genes in this study involve multiple pathways that play a critical role in cancer development. This is understandable because oncogenesis is the result of several altered pathways that cannot be concluded with one single biomarker. By proposing a multi-gene prediction panel, this study provided insight regarding immunology in cancer development and progression.

While gene status only represents part of the bigger picture, patients’ clinical features are also closely linked to their oncological outcomes. When the IRG signature was incorporated with independent clinical risk factors, the model presented good performance. The calibration curve also showed good agreement between model prediction and reality in the training set. Compared to traditional clinical risk scoring, incorporating our IRG signature with clinical risk factors would benefit prognostic prediction. For those anticipated to have significantly inferior survival, a more close-up surveillance strategy should be made to identify early onset of tumor recurrence after resection or tumor progression during non-surgical intervention. In addition, surgeons would be more informed when making treatment decisions.

We used bioinformatics tools to explore the high-dimensional connections and functions of the selected IRGs. We introduced 446 CEGs of the selected IRGs via a co-expression network and conducted a comprehensive interpretation of these genes regarding cellular functions and pathway enrichment. Immune cell adhesion, immune cell function regulation, and cytokine regulation were the most enriched functions based on GO analyses (Figure 6). The CEG showing the highest correlation was enriched in interleukin regulation (regulation of interleukin-10 and interleukin-10 regulation) and the most enriched cell functions were closely linked with RNA processing (GO terms, RNA binding, and ncRNA metabolic process) and immune regulation (GO terms, response to cytokine, regulation of cytokine production, and regulation of defense response).

Our study also has several limitations. First, the gene levels in different cohorts were not measured via universal sequencing protocols, which might have led to some inconsistencies, and the minor drift of calibration in the validation group to some extent might explain the slight decrease in C-index in the test group. It is important to recognize that microarray protocols among databases were not consistent and that different ethnic and geographical variations could result in reasonable inter-cohort bias. Second, in contrast to the volume of gene data, the clinical information in these databases was relatively limited, and it is best to combine the gene signature with more comprehensive clinical factors for optimal prognostic prediction.

## Conclusion

Taken together, we developed a predictive IRG panel that can legitimately forecast CRC patients with CRC, and the gene signature was more robust when incorporated with clinical risk factors. Our model could potentially benefit individualized clinical management for patients with CRC. For instance, a shorter check-up interval should be considered for patients with adverse survival, as timely medical intervention would be ideal for tumor progression or recurrence.

## Data Availability Statement

The original contributions presented in the study are included in the article/Supplementary Material, further inquiries can be directed to the corresponding author/s.

## Author Contributions

The data gathering was the joint effort of SD and SX. The bioinformatics analysis was performed by SD and YY. The construction of this article was accomplished by SD and SX. The study was conducted under the supervision of KD as the corresponding author. All authors contributed to the article and approved the submitted version.

## Conflict of Interest

The authors declare that the research was conducted in the absence of any commercial or financial relationships that could be construed as a potential conflict of interest.

## Footnotes

1 http://cancergenome.nih.gov/

2 https://xenabrowser.net/datapages/

3 https://immport.niaid.nih.gov

## Supplementary Material

Supplementary Figure 1 ROC curves of IRG signature and TNM staging in training and validation group. **(A)** In the training group, the IRG signature yielded an AUC of 0.746, and the AUC of TNM staging was 0.704. **(B)** The AUC values with IRG signature and TNM staging were 0.622 and 0.609, respectively, in the validation set. In both datasets, the IRG signature showed higher prediction accuracy than traditional TNM staging.

Supplementary Table 1 Univariate Cox regression analysis of IRGs in the Training set. Univariate Cox regression was performed for each IRG regarding survival status to screen for prognostic immune-related genes. The coefficient values, HRs, 95% CIs, and corresponding *p-*values are given. IRGs with a *p* < 0.05 were enrolled for further model construction.

Supplementary Table 2 The combination of the IRGs in the training datasets for signature selection. The full-size recombination of the nine IRGs selected by RSFA (2^9^ − 1 = 511). The AUC of each combination regarding patient survival was calculated. The six-IRG model with the highest AUC value (0.746) was designated as the prediction panel.

Supplementary Table 3 Functional enrichment analyses using co-expressing genes of selected IRGs. Functional enrichment analyses were used to explore the role of co-expressing genes of the selected IRGs.

## Abbreviations

TCGA: The Cancer Genome Atlas
GEO: Gene Expression Omnibus
IRG: Immune-related gene
RSFA: random survival forest algorithm
GO: gene ontology
PPI: protein-protein interaction
TIIC: tumor infiltrating immune cells
KM: Kaplan-Meier
ROC: receiver operating characteristic
AUC: area under curve
PD-1: programmed death ligand
TMB: tumor mutation burden
dMMR: microsatellite instability/mismatch repair deficiency
CEG: co-expressing gene
IL: Interleukin.
