# Supplementary figures and images for: Identification of an Immune-Related Gene Signature to Improve Prognosis Prediction in Colorectal Cancer Patients
Source: Front Genet. 2020 Dec 4;11:607009. doi: 10.3389/fgene.2020.607009 (PMC7746810; doi:10.3389/fgene.2020.607009)

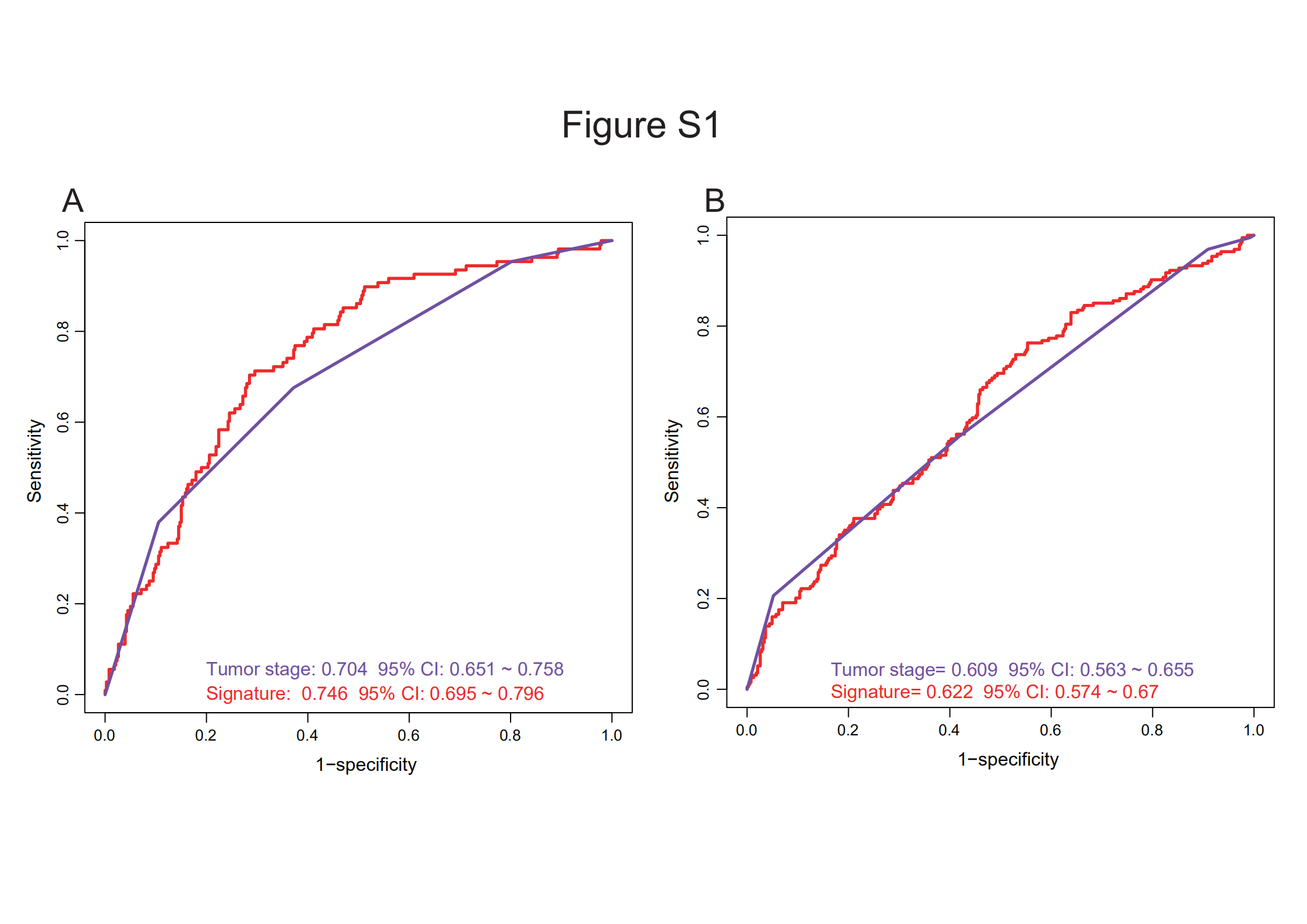

Supplement: Supplementary Figure 1 — ROC curves of IRG signature and TNM staging in training and validation group. (A) In the training group, the IRG signature yielded an AUC of 0.746, and the AUC of TNM staging was 0.704. (B) The AUC values with IRG signature and TNM staging were 0.622 and 0.609, respectively, in the validation set. In both datasets, the IRG signature showed higher prediction accuracy than traditional TNM staging. [file Image_1.TIF]
