# Supplementary material for: Identification of an Immune-Related Gene Signature to Improve Prognosis Prediction in Colorectal Cancer Patients
Source: Front Genet. 2020 Dec 4;11:607009. doi: 10.3389/fgene.2020.607009 (PMC7746810; doi:10.3389/fgene.2020.607009)
Supplement: Supplementary Table 1 — Univariate Cox regression analysis of IRGs in the Training set. Univariate Cox regression was performed for each IRG regarding survival status to screen for prognostic immune-related genes. The coefficient values, HRs, 95% CIs, and corresponding p-values are given. IRGs with a p < 0.05 were enrolled for further model construction. [file Table_1.DOC]

| **Supplementary table S1: Univariate Cox regression analysis of IRGs in the Training set** | | | | | |
| --- | --- | --- | --- | --- | --- |
| IRGs | coefficient | *P* | HR | right | left |
| ENSG00000167578 | 0.224816088 | 0.352680921 | 1.25209242 | 0.77935694 | 2.011575631 |
| ENSG00000064601 | 0.080759592 | 0.52620813 | 1.084110237 | 0.844526186 | 1.391662005 |
| ENSG00000008517 | -0.026551201 | 0.798957272 | 0.973798183 | 0.793842502 | 1.194547908 |
| ENSG00000167768 | -0.221352342 | 0.571567527 | 0.801434251 | 0.372244272 | 1.725471441 |
| ENSG00000114861 | -0.006885552 | 0.967957993 | 0.9931381 | 0.709746277 | 1.389684337 |
| ENSG00000142507 | 0.087394105 | 0.575706145 | 1.091326692 | 0.803593871 | 1.482084411 |
| ENSG00000127329 | -0.001714735 | 0.99373397 | 0.998286735 | 0.650730322 | 1.531473747 |
| ENSG00000108774 | -0.221222658 | 0.309741081 | 0.801538191 | 0.523048299 | 1.228306207 |
| ENSG00000076984 | 0.576128204 | 0.0259013 | 1.779136624 | 1.07169264 | 2.953577366 |
| ENSG00000009724 | -0.451655838 | 0.294523542 | 0.636573216 | 0.273586101 | 1.48116245 |
| ENSG00000072694 | 0.190255811 | 0.286428005 | 1.209558977 | 0.852523406 | 1.71612053 |
| ENSG00000110395 | -0.088458138 | 0.637835747 | 0.915341428 | 0.633325382 | 1.322937551 |
| ENSG00000104998 | 0.126684755 | 0.324945871 | 1.13505914 | 0.882001343 | 1.460722552 |
| ENSG00000143184 | 0.654016306 | 0.001286833 | 1.923249697 | 1.291482418 | 2.864064848 |
| ENSG00000182220 | -0.075857107 | 0.666283177 | 0.926948651 | 0.65664479 | 1.308521465 |
| ENSG00000158481 | -0.208712122 | 0.245255851 | 0.811628852 | 0.570772028 | 1.154123472 |
| ENSG00000168811 | 0.361290376 | 0.22955609 | 1.435180142 | 0.796070002 | 2.587388086 |
| ENSG00000007312 | 0.062840642 | 0.578999079 | 1.064857133 | 0.852876909 | 1.329524461 |
| ENSG00000149925 | 0.220287453 | 0.130214528 | 1.246434971 | 0.937041212 | 1.657984853 |
| ENSG00000186265 | 0.056596621 | 0.887493668 | 1.058228857 | 0.483128125 | 2.317911657 |
| ENSG00000135905 | -0.073063841 | 0.677060704 | 0.929541485 | 0.659082854 | 1.310984449 |
| ENSG00000184979 | 0.116559354 | 0.357570685 | 1.123624201 | 0.876553872 | 1.440335141 |
| ENSG00000111530 | -0.289490973 | 0.172200192 | 0.748644551 | 0.494053501 | 1.134429091 |
| ENSG00000056972 | -0.02823176 | 0.894915084 | 0.972163032 | 0.639451027 | 1.477988024 |
| ENSG00000142875 | 0.014864039 | 0.861674482 | 1.014975058 | 0.858701966 | 1.199687912 |
| ENSG00000163464 | -0.167776077 | 0.351164793 | 0.84554315 | 0.594236656 | 1.203128774 |
| ENSG00000164104 | 0.008923957 | 0.947966207 | 1.008963894 | 0.771756171 | 1.319080012 |
| ENSG00000177889 | -0.339023121 | 0.075516506 | 0.712465976 | 0.490227031 | 1.035454461 |
| ENSG00000153898 | -0.015734705 | 0.870406639 | 0.984388439 | 0.814835404 | 1.189222503 |
| ENSG00000116690 | 0.480013149 | 0.002987946 | 1.616095651 | 1.17719359 | 2.218636916 |
| ENSG00000163221 | 0.063259458 | 0.598888507 | 1.065303205 | 0.84159527 | 1.348475876 |
| ENSG00000090382 | -0.001702604 | 0.970307377 | 0.998298844 | 0.912695312 | 1.091931304 |
| ENSG00000121060 | -0.272751346 | 0.148334914 | 0.76128206 | 0.52592705 | 1.101959624 |
| ENSG00000112697 | -0.013407444 | 0.936800341 | 0.986682036 | 0.708350252 | 1.374378616 |
| ENSG00000015285 | 0.136079351 | 0.265231717 | 1.145772808 | 0.901843796 | 1.455679281 |
| ENSG00000101425 | 0.569160459 | 0.584835064 | 1.766783141 | 0.229308743 | 13.61275036 |
| ENSG00000124107 | 0.096856667 | 0.19050852 | 1.101702452 | 0.95298297 | 1.273630622 |
| ENSG00000125686 | 0.139694157 | 0.377375218 | 1.14992205 | 0.843268081 | 1.568090564 |
| ENSG00000258227 | -0.162523644 | 0.284730298 | 0.849995993 | 0.631098298 | 1.144818785 |
| ENSG00000064932 | 0.173254507 | 0.253312441 | 1.189168718 | 0.883374675 | 1.600818182 |
| ENSG00000104870 | 0.035598443 | 0.707328554 | 1.036239654 | 0.860503023 | 1.247866181 |
| ENSG00000138772 | -0.194884494 | 0.047919584 | 0.822929706 | 0.678423146 | 0.99821668 |
| ENSG00000140443 | 0.176789157 | 0.281423784 | 1.193379451 | 0.86510841 | 1.646215084 |
| ENSG00000155926 | -0.082134665 | 0.55800709 | 0.921147904 | 0.699815845 | 1.212481064 |
| ENSG00000105397 | 0.321545125 | 0.13178062 | 1.379257244 | 0.907905151 | 2.095318593 |
| ENSG00000100804 | 0.019632001 | 0.905939219 | 1.019825976 | 0.73638255 | 1.412370542 |
| ENSG00000134250 | 0.122172079 | 0.429477261 | 1.129948525 | 0.834519102 | 1.529963385 |
| ENSG00000079134 | 0.0457377 | 0.836812753 | 1.046799799 | 0.677403881 | 1.617631447 |
| ENSG00000163508 | 0.261159452 | 0.341929006 | 1.298434687 | 0.75772153 | 2.225002943 |
| ENSG00000154451 | -0.01561771 | 0.8893648 | 0.984503614 | 0.790046385 | 1.226823367 |
| ENSG00000243649 | -0.070096893 | 0.563776359 | 0.932303482 | 0.734839123 | 1.182830031 |
| ENSG00000143162 | 0.017324772 | 0.92432477 | 1.017475716 | 0.711661309 | 1.454704395 |
| ENSG00000055332 | -0.047693336 | 0.76935408 | 0.953426124 | 0.693163783 | 1.311409216 |
| ENSG00000115415 | -0.00417844 | 0.96915243 | 0.995830278 | 0.805773432 | 1.230715611 |
| ENSG00000137496 | 0.22017319 | 0.136965336 | 1.246292558 | 0.932400325 | 1.665856497 |
| ENSG00000160224 | -0.108137271 | 0.766088937 | 0.897504387 | 0.440180276 | 1.82996415 |
| ENSG00000140931 | 0.080720286 | 0.514642662 | 1.084067626 | 0.850381072 | 1.381971749 |
| ENSG00000109323 | 0.042056384 | 0.849486755 | 1.042953283 | 0.675502361 | 1.61028534 |
| ENSG00000213145 | 0.186983701 | 0.107576442 | 1.205607635 | 0.960059432 | 1.513958117 |
| ENSG00000196954 | -0.111582924 | 0.49263764 | 0.89441722 | 0.650294587 | 1.230184257 |
| ENSG00000196950 | -0.189423149 | 0.16143485 | 0.827436304 | 0.63472652 | 1.078654846 |
| ENSG00000161911 | -0.25810846 | 0.65494397 | 0.772511441 | 0.249055358 | 2.396149727 |
| ENSG00000128340 | 0.040180935 | 0.728529546 | 1.04099911 | 0.829676244 | 1.306147011 |
| ENSG00000118503 | -0.13900611 | 0.309440984 | 0.870222712 | 0.66560547 | 1.137742405 |
| ENSG00000143369 | 0.050039545 | 0.630380742 | 1.051312669 | 0.857461407 | 1.288989009 |
| ENSG00000153283 | 0.002270328 | 0.990054405 | 1.002272907 | 0.701382329 | 1.432244495 |
| ENSG00000112799 | 0.155809366 | 0.243348033 | 1.168603406 | 0.899472104 | 1.518261561 |
| ENSG00000180549 | 0.255874137 | 0.43927884 | 1.291590154 | 0.675327901 | 2.470215023 |
| ENSG00000257103 | -0.263961709 | 0.304931777 | 0.768002946 | 0.463824658 | 1.271662719 |
| ENSG00000163531 | 0.277612058 | 0.164231489 | 1.319974024 | 0.892652482 | 1.951858601 |
| ENSG00000170043 | 0.200065747 | 0.231628072 | 1.221483065 | 0.880075954 | 1.695331942 |
| ENSG00000114812 | -0.048719171 | 0.661165912 | 0.952448567 | 0.765997266 | 1.184283957 |
| ENSG00000187474 | -0.017830764 | 0.838028133 | 0.982327263 | 0.827957166 | 1.165479195 |
| ENSG00000091592 | 0.354148893 | 0.025996071 | 1.424967338 | 1.043273422 | 1.946308486 |
| ENSG00000177425 | -0.347720792 | 0.047674408 | 0.706296053 | 0.500635059 | 0.996442628 |
| ENSG00000123384 | 0.045846053 | 0.682430877 | 1.046913229 | 0.840487947 | 1.304036915 |
| ENSG00000169035 | -0.071323043 | 0.364897104 | 0.931161038 | 0.798033396 | 1.086496985 |
| ENSG00000151651 | 0.187133069 | 0.069422669 | 1.205787728 | 0.98523711 | 1.475709786 |
| ENSG00000124731 | -0.098515808 | 0.447845427 | 0.906181368 | 0.702641158 | 1.16868285 |
| ENSG00000185885 | -0.013747964 | 0.859157206 | 0.986346107 | 0.847386027 | 1.148093799 |
| ENSG00000137834 | 0.226934276 | 0.218811584 | 1.254747398 | 0.873918805 | 1.801530101 |
| ENSG00000164687 | -0.061237635 | 0.605420175 | 0.940599694 | 0.745603399 | 1.186593015 |
| ENSG00000185201 | 0.030760859 | 0.724453997 | 1.031238863 | 0.869124349 | 1.223591991 |
| ENSG00000140368 | 0.082755328 | 0.697145455 | 1.086275994 | 0.716042728 | 1.647940116 |
| ENSG00000096717 | -0.035213255 | 0.856236836 | 0.965399518 | 0.659567819 | 1.413040785 |
| ENSG00000101695 | 0.033329342 | 0.819648544 | 1.033890987 | 0.776324627 | 1.376911842 |
| ENSG00000241839 | 0.173063216 | 0.182617651 | 1.188941262 | 0.921780097 | 1.533534224 |
| ENSG00000121053 | 0.448385196 | 0.492113338 | 1.565781712 | 0.43564938 | 5.627627357 |
| ENSG00000162692 | 0.006887491 | 0.955580535 | 1.006911265 | 0.790201605 | 1.283052691 |
| ENSG00000109861 | -0.043784022 | 0.795050783 | 0.957160661 | 0.687873608 | 1.33186754 |
| ENSG00000055130 | -0.016701318 | 0.95036442 | 0.983437376 | 0.581260894 | 1.663881197 |
| ENSG00000068903 | 0.216822877 | 0.376234944 | 1.242124073 | 0.76840189 | 2.007897474 |
| ENSG00000104856 | 0.133821367 | 0.380924896 | 1.143188591 | 0.847448924 | 1.54213442 |
| ENSG00000103187 | 0.255605404 | 0.048731778 | 1.291243109 | 1.001423288 | 1.664939078 |
| ENSG00000005381 | 0.582162396 | 0.173675028 | 1.789904732 | 0.773740307 | 4.14061271 |
| ENSG00000163661 | 0.203876723 | 0.315524394 | 1.226146988 | 0.823458417 | 1.825758782 |
| ENSG00000091972 | 0.028038357 | 0.801363357 | 1.028435132 | 0.826632421 | 1.27950319 |
| ENSG00000174130 | 0.09320417 | 0.733058181 | 1.097685827 | 0.642486008 | 1.87539364 |
| ENSG00000048462 | -0.086747918 | 0.357770674 | 0.916908202 | 0.762135131 | 1.103112318 |
| ENSG00000239697 | 0.256101595 | 0.045015108 | 1.29188397 | 1.005707954 | 1.65949189 |
| ENSG00000176407 | 0.036197234 | 0.917902801 | 1.03686033 | 0.520958323 | 2.063657106 |
| ENSG00000140575 | 0.041055268 | 0.817060251 | 1.041909689 | 0.735803159 | 1.4753617 |
| ENSG00000140464 | 0.238222415 | 0.130165212 | 1.268991404 | 0.932130228 | 1.727590346 |
| ENSG00000157873 | 0.232988799 | 0.181186378 | 1.26236734 | 0.897151254 | 1.776257117 |
| ENSG00000158477 | -1.12300666 | 0.003245118 | 0.325300255 | 0.154004893 | 0.687122691 |
| ENSG00000174004 | 0.187631927 | 0.281263065 | 1.206389395 | 0.857552049 | 1.697127741 |
| ENSG00000151882 | -0.098898723 | 0.214515771 | 0.905834443 | 0.774867664 | 1.058937 |
| ENSG00000105401 | 0.139816695 | 0.40583486 | 1.150062967 | 0.827081521 | 1.599171055 |
| ENSG00000058063 | -0.095796027 | 0.560025563 | 0.908649337 | 0.658390303 | 1.254033684 |
| ENSG00000181481 | 0.312160192 | 0.123575385 | 1.366373558 | 0.918380758 | 2.032900498 |
| ENSG00000152778 | -0.1500228 | 0.312476451 | 0.860688353 | 0.643302979 | 1.151532738 |
| ENSG00000149295 | 0.005870785 | 0.958740782 | 1.005888052 | 0.805294621 | 1.256447947 |
| ENSG00000113558 | -0.210648097 | 0.307482495 | 0.810059079 | 0.540527548 | 1.213991248 |
| ENSG00000138755 | -0.032364662 | 0.589005207 | 0.968153469 | 0.860902726 | 1.088765444 |
| ENSG00000204642 | 0.045325241 | 0.596357477 | 1.046368126 | 0.884792949 | 1.237449119 |
| ENSG00000164406 | 0.0358545 | 0.883546533 | 1.036505024 | 0.641522766 | 1.674675819 |
| ENSG00000130508 | 0.111022203 | 0.291399056 | 1.117419717 | 0.909171043 | 1.373368447 |
| ENSG00000163565 | 0.131119161 | 0.179469899 | 1.140103629 | 0.941461107 | 1.380658504 |
| ENSG00000124102 | -0.030253856 | 0.478219017 | 0.970199212 | 0.892375957 | 1.054809358 |
| ENSG00000140968 | 0.006399344 | 0.956527848 | 1.006419864 | 0.799562562 | 1.266793858 |
| ENSG00000253729 | -0.114313817 | 0.364795603 | 0.891977994 | 0.696603865 | 1.142148045 |
| ENSG00000167770 | -0.038624657 | 0.814334812 | 0.962111763 | 0.696987615 | 1.328085356 |
| ENSG00000145349 | 0.013618978 | 0.932398629 | 1.013712139 | 0.740039348 | 1.388591435 |
| ENSG00000139725 | 0.016270405 | 0.922053571 | 1.016403489 | 0.733711314 | 1.408014341 |
| ENSG00000115085 | 0.167892182 | 0.345229315 | 1.182809075 | 0.834657155 | 1.676182011 |
| ENSG00000116586 | 0.050902087 | 0.770223201 | 1.052219862 | 0.747765283 | 1.480633915 |
| ENSG00000010810 | 0.059615288 | 0.658713962 | 1.061428123 | 0.814709951 | 1.382859827 |
| ENSG00000116584 | 0.369127523 | 0.069346136 | 1.446472051 | 0.971194968 | 2.154337145 |
| ENSG00000162645 | 0.008255668 | 0.940804133 | 1.00828984 | 0.810875538 | 1.253766275 |
| ENSG00000276070 | -0.123463005 | 0.24252615 | 0.883854339 | 0.718552223 | 1.087184018 |
| ENSG00000135046 | 0.021191487 | 0.816456806 | 1.021417621 | 0.854058056 | 1.221572643 |
| ENSG00000012223 | 0.017834786 | 0.890616545 | 1.017994775 | 0.789510017 | 1.312603184 |
| ENSG00000096696 | -0.023181954 | 0.871299741 | 0.977084684 | 0.738129217 | 1.293397492 |
| ENSG00000159842 | 0.110757667 | 0.541420741 | 1.117124158 | 0.782919976 | 1.593989709 |
| ENSG00000196262 | -0.005191787 | 0.976044661 | 0.994821667 | 0.708882725 | 1.396098556 |
| ENSG00000137575 | -0.045585296 | 0.749281018 | 0.955438104 | 0.722418663 | 1.26361903 |
| ENSG00000161940 | 0.250719631 | 0.113813228 | 1.284949773 | 0.941725454 | 1.753266743 |
| ENSG00000205213 | -0.085125149 | 0.527474839 | 0.918397341 | 0.705271636 | 1.195927403 |
| ENSG00000187116 | 0.068291768 | 0.62040304 | 1.070677652 | 0.817128732 | 1.402900901 |
| ENSG00000005206 | 0.281252797 | 0.061733951 | 1.324788464 | 0.986280427 | 1.779478155 |
| ENSG00000166949 | 0.067578287 | 0.729633395 | 1.069914016 | 0.729309942 | 1.569587822 |
| ENSG00000085514 | 0.025008593 | 0.832964738 | 1.025323931 | 0.812691422 | 1.293589591 |
| ENSG00000172156 | -0.074820528 | 0.365075387 | 0.927910005 | 0.78920588 | 1.090991589 |
| ENSG00000084072 | 0.079536175 | 0.736346536 | 1.082784728 | 0.681500636 | 1.720354621 |
| ENSG00000023318 | -0.198048822 | 0.375802159 | 0.820329802 | 0.529230198 | 1.271546836 |
| ENSG00000004139 | 0.141866986 | 0.708964046 | 1.15242335 | 0.547117473 | 2.427412105 |
| ENSG00000227507 | 0.053079564 | 0.605917968 | 1.054513543 | 0.861937651 | 1.290115138 |
| ENSG00000244509 | 0.154187551 | 0.096393033 | 1.166709684 | 0.972799329 | 1.39927264 |
| ENSG00000135334 | -0.191132379 | 0.36603446 | 0.826023233 | 0.545768387 | 1.250190369 |
| ENSG00000167984 | -0.014530352 | 0.955105755 | 0.985574705 | 0.594278895 | 1.634514546 |
| ENSG00000082074 | -0.03265521 | 0.781386427 | 0.967872215 | 0.768521919 | 1.218932863 |
| ENSG00000198001 | -0.319509609 | 0.170128315 | 0.726505221 | 0.460237392 | 1.146820848 |
| ENSG00000107201 | 0.029682443 | 0.835765111 | 1.030127358 | 0.778070077 | 1.363839075 |
| ENSG00000173585 | 0.622285244 | 0.332002315 | 1.863181003 | 0.52994721 | 6.550545765 |
| ENSG00000092020 | -0.076756112 | 0.687135428 | 0.926115695 | 0.637443097 | 1.345516618 |
| ENSG00000167613 | 0.108247242 | 0.435011153 | 1.114323219 | 0.84914203 | 1.462318661 |
| ENSG00000235568 | 0.134754611 | 0.419783062 | 1.144255962 | 0.824806469 | 1.587428998 |
| ENSG00000149636 | -0.242331248 | 0.096079144 | 0.784796172 | 0.58993927 | 1.044014297 |
| ENSG00000164308 | -0.032917744 | 0.655029327 | 0.967618149 | 0.837510548 | 1.117938018 |
| ENSG00000015479 | -0.207913982 | 0.400529079 | 0.812276904 | 0.500250055 | 1.318927929 |
| ENSG00000096968 | -0.050356055 | 0.72486241 | 0.950890795 | 0.718371651 | 1.258670637 |
| ENSG00000240505 | 0.135443168 | 0.623757067 | 1.145044119 | 0.666489212 | 1.967212686 |
| ENSG00000137078 | 0.027609599 | 0.823776911 | 1.027994276 | 0.806225945 | 1.310764355 |
| ENSG00000111537 | -0.045784878 | 0.850999581 | 0.955247435 | 0.592439635 | 1.540237362 |
| ENSG00000061938 | 0.415449716 | 0.00879004 | 1.515051931 | 1.110349129 | 2.067261812 |
| ENSG00000186407 | 0.031825149 | 0.847680769 | 1.032336984 | 0.746083355 | 1.428419011 |
| ENSG00000073008 | 0.039518102 | 0.817484601 | 1.040309331 | 0.74371425 | 1.455187262 |
| ENSG00000030582 | 0.243502999 | 0.079196035 | 1.275710144 | 0.972017911 | 1.674286404 |
| ENSG00000105246 | 0.180799114 | 0.216281674 | 1.198174458 | 0.89961414 | 1.595819772 |
| ENSG00000186470 | 0.140225823 | 0.338036159 | 1.150533587 | 0.863600338 | 1.532801084 |
| ENSG00000197879 | 0.195262488 | 0.220355349 | 1.215630033 | 0.889582575 | 1.661179544 |
| ENSG00000160072 | 0.074631577 | 0.627454365 | 1.077487107 | 0.797102267 | 1.456498763 |
| ENSG00000070831 | -0.336658942 | 0.034451433 | 0.714152366 | 0.522736939 | 0.975660152 |
| ENSG00000033327 | -0.023262641 | 0.904880035 | 0.977005849 | 0.667106835 | 1.43086591 |
| ENSG00000125730 | 0.083419174 | 0.159272741 | 1.086997354 | 0.967788766 | 1.220889608 |
| ENSG00000132274 | -0.009476376 | 0.927028977 | 0.990568383 | 0.808739153 | 1.213278371 |
| ENSG00000105649 | 0.311493375 | 0.053848217 | 1.36546274 | 0.994858192 | 1.874124885 |
| ENSG00000054967 | 0.068368544 | 0.746726405 | 1.070759858 | 0.707127257 | 1.62138662 |
| ENSG00000160959 | 0.211739226 | 0.243531455 | 1.235825572 | 0.865786561 | 1.764020041 |
| ENSG00000185303 | -0.116413798 | 0.441208568 | 0.890106823 | 0.661877262 | 1.197034861 |
| ENSG00000130159 | 0.174754538 | 0.266613409 | 1.190953847 | 0.874969123 | 1.621052706 |
| ENSG00000110169 | 0.3157656 | 0.435720093 | 1.371308782 | 0.619866382 | 3.033698604 |
| ENSG00000105383 | 0.22246477 | 0.499121035 | 1.249151811 | 0.655301133 | 2.38116519 |
| ENSG00000123737 | -0.253604097 | 0.213866872 | 0.775998962 | 0.520228822 | 1.157518312 |
| ENSG00000167207 | -0.301405077 | 0.095777313 | 0.739778045 | 0.518891203 | 1.054694226 |
| ENSG00000115318 | 0.465685203 | 0.043676741 | 1.593105415 | 1.013296465 | 2.504681454 |
| ENSG00000145824 | -0.088628046 | 0.059624116 | 0.915185917 | 0.834559682 | 1.003601397 |
| ENSG00000183018 | -0.032531569 | 0.719484768 | 0.96799189 | 0.810531512 | 1.15604179 |
| ENSG00000243678 | -0.005097897 | 0.964586483 | 0.994915075 | 0.794420857 | 1.246009592 |
| ENSG00000108700 | 0.11195521 | 0.242723218 | 1.118462764 | 0.926929221 | 1.34957333 |
| ENSG00000254126 | -0.045991233 | 0.857899924 | 0.955050335 | 0.577274252 | 1.580048198 |
| ENSG00000173535 | -0.425120737 | 0.034501349 | 0.653690856 | 0.440768808 | 0.969469088 |
| ENSG00000173801 | 0.078999037 | 0.590064023 | 1.08220328 | 0.81188075 | 1.442531972 |
| ENSG00000118640 | 0.110193259 | 0.478601276 | 1.116493822 | 0.823149535 | 1.514376672 |
| ENSG00000111796 | -0.09111508 | 0.46972186 | 0.912912647 | 0.713095314 | 1.168721045 |
| ENSG00000132155 | 0.07396952 | 0.828751655 | 1.076773985 | 0.550855274 | 2.104803691 |
| ENSG00000127666 | 0.168665466 | 0.31432597 | 1.183724076 | 0.8522458 | 1.644129767 |
| ENSG00000104972 | 0.101928295 | 0.576148395 | 1.107304069 | 0.774575703 | 1.58295993 |
| ENSG00000134516 | -0.035085038 | 0.8266843 | 0.965523306 | 0.70529057 | 1.321774734 |
| ENSG00000136689 | -0.082806404 | 0.282842114 | 0.920529341 | 0.791419145 | 1.070702261 |
| ENSG00000173692 | -0.243050655 | 0.317572081 | 0.784231787 | 0.486908657 | 1.263110622 |
| ENSG00000147813 | 0.176925351 | 0.107606699 | 1.193541993 | 0.962148928 | 1.480584189 |
| ENSG00000173193 | -0.009641404 | 0.939813359 | 0.990404925 | 0.771116773 | 1.272053664 |
| ENSG00000108828 | 0.325945911 | 0.010266735 | 1.385340435 | 1.080097045 | 1.776847858 |
| ENSG00000161955 | 0.030936943 | 0.795128269 | 1.031420464 | 0.81661814 | 1.302724151 |
| ENSG00000106683 | 0.402868681 | 0.02622233 | 1.496110411 | 1.048807581 | 2.134182095 |
| ENSG00000178789 | 0.30087331 | 0.44708726 | 1.351038168 | 0.622032369 | 2.934419849 |
| ENSG00000174775 | 0.202048421 | 0.151702921 | 1.223907269 | 0.928489973 | 1.613317374 |
| ENSG00000101182 | -0.092485655 | 0.410209097 | 0.911662288 | 0.7315436 | 1.136129314 |
| ENSG00000184584 | -0.051818406 | 0.662044311 | 0.949501275 | 0.752633374 | 1.197864329 |
| ENSG00000117586 | -0.039980362 | 0.796774339 | 0.960808307 | 0.708742484 | 1.302521894 |
| ENSG00000161057 | -0.035865984 | 0.86434978 | 0.964769579 | 0.639326787 | 1.455875712 |
| ENSG00000137462 | -0.116709711 | 0.389145582 | 0.889843468 | 0.682267359 | 1.160573471 |
| ENSG00000105697 | 0.844894606 | 0.034237145 | 2.327732473 | 1.064783958 | 5.088674023 |
| ENSG00000213658 | 1.327614945 | 0.044904929 | 3.772036138 | 1.030632478 | 13.80536412 |
| ENSG00000110934 | 0.112383464 | 0.510358217 | 1.118941852 | 0.800733383 | 1.563605183 |
| ENSG00000124406 | -0.0337261 | 0.783789942 | 0.966836285 | 0.759847975 | 1.230209768 |
| ENSG00000081041 | -0.169529423 | 0.018894582 | 0.844061919 | 0.732665616 | 0.972395193 |
| ENSG00000244038 | -0.161917568 | 0.294494817 | 0.850511311 | 0.628358277 | 1.151205477 |
| ENSG00000124469 | -0.098153997 | 0.681352745 | 0.906509293 | 0.567415581 | 1.44824909 |
| ENSG00000198053 | 0.052304048 | 0.595441934 | 1.053696068 | 0.868692061 | 1.278100092 |
| ENSG00000169224 | -1.355481422 | 0.163763993 | 0.257823143 | 0.038261569 | 1.737324806 |
| ENSG00000019582 | 0.000974012 | 0.990018461 | 1.000974486 | 0.859312942 | 1.165989564 |
| ENSG00000108344 | 0.029808875 | 0.846850819 | 1.030257607 | 0.761327718 | 1.394183756 |
| ENSG00000148175 | 0.044294106 | 0.666557365 | 1.045289736 | 0.854541953 | 1.278615553 |
| ENSG00000122223 | -0.157326387 | 0.540251932 | 0.85442514 | 0.516429023 | 1.413635345 |
| ENSG00000173432 | 0.019667682 | 0.69583165 | 1.019862365 | 0.924102611 | 1.125545186 |
| ENSG00000067560 | -0.399051859 | 0.111693493 | 0.670955905 | 0.410343021 | 1.097086593 |
| ENSG00000176208 | -0.290304022 | 0.139820824 | 0.748036113 | 0.508812464 | 1.099733333 |
| ENSG00000175354 | -0.157882656 | 0.478352317 | 0.853949982 | 0.55191454 | 1.321274435 |
| ENSG00000090104 | 0.02142604 | 0.80230076 | 1.021657226 | 0.863896437 | 1.208227562 |
| ENSG00000157837 | -0.05322374 | 0.862755114 | 0.948167846 | 0.518575286 | 1.733638853 |
| ENSG00000158485 | -1.129402953 | 0.013516102 | 0.32322618 | 0.131909532 | 0.792021331 |
| ENSG00000213886 | -0.042907292 | 0.490980882 | 0.9580002 | 0.847886998 | 1.08241356 |
| ENSG00000184588 | -0.146664507 | 0.31514258 | 0.863583655 | 0.648666346 | 1.149707756 |
| ENSG00000184304 | 0.11043918 | 0.540359222 | 1.116768425 | 0.784198945 | 1.590376681 |
| ENSG00000129048 | -0.191986481 | 0.399259761 | 0.825318026 | 0.528146173 | 1.289699479 |
| ENSG00000000971 | 0.078966948 | 0.467014409 | 1.082168554 | 0.874744171 | 1.338778603 |
| ENSG00000186105 | -0.080147231 | 0.859860701 | 0.922980445 | 0.379124151 | 2.247002465 |
| ENSG00000144230 | 0.056339584 | 0.852265693 | 1.057956888 | 0.58472859 | 1.914174874 |
| ENSG00000188211 | -0.023798299 | 0.841595215 | 0.976482648 | 0.773222537 | 1.233174558 |
| ENSG00000100554 | 0.070256261 | 0.725379073 | 1.072783059 | 0.724887254 | 1.587644816 |
| ENSG00000167657 | 0.302438873 | 0.074543733 | 1.35315496 | 0.970476101 | 1.886732031 |
| ENSG00000121879 | 0.172427066 | 0.357938162 | 1.188185159 | 0.822678238 | 1.716082797 |
| ENSG00000118271 | 0.051531028 | 0.763654963 | 1.052881854 | 0.752491501 | 1.473186339 |
| ENSG00000175104 | 0.068160709 | 0.780087734 | 1.07053734 | 0.663441551 | 1.727432044 |
| ENSG00000116266 | -0.14189936 | 0.4054933 | 0.867708578 | 0.621117245 | 1.212199762 |
| ENSG00000089692 | 0.31507453 | 0.016112177 | 1.37036144 | 1.060190849 | 1.771275876 |
| ENSG00000213853 | -0.269244916 | 0.093253913 | 0.763956127 | 0.557862294 | 1.046188226 |
| ENSG00000167601 | 0.170588141 | 0.121499596 | 1.186002183 | 0.955684987 | 1.471825127 |
| ENSG00000197746 | 0.257395982 | 0.144400407 | 1.293557251 | 0.915542979 | 1.827648073 |
| ENSG00000100413 | -0.003463998 | 0.987031654 | 0.996541995 | 0.656283977 | 1.513210717 |
| ENSG00000076242 | -0.037184477 | 0.763915199 | 0.963498376 | 0.755904074 | 1.228104401 |
| ENSG00000108622 | 0.276228765 | 0.092654963 | 1.318149376 | 0.955296036 | 1.818826534 |
| ENSG00000197046 | 0.085950772 | 0.557402814 | 1.089752681 | 0.81776554 | 1.452202186 |
| ENSG00000241258 | 0.132907274 | 0.536479173 | 1.142144087 | 0.749384023 | 1.74075384 |
| ENSG00000132639 | 0.606014059 | 0.001344555 | 1.833110148 | 1.265625107 | 2.655045952 |
| ENSG00000131473 | -0.083742601 | 0.68170296 | 0.919667947 | 0.616357153 | 1.372238693 |
| ENSG00000110917 | -0.154933166 | 0.372376618 | 0.856472417 | 0.609356743 | 1.203802223 |
| ENSG00000096384 | 0.039387418 | 0.813356504 | 1.040173388 | 0.750071741 | 1.442476257 |
| ENSG00000154370 | 0.386228604 | 0.15823932 | 1.471421005 | 0.860484769 | 2.516116322 |
| ENSG00000099866 | 0.072133928 | 0.658072529 | 1.074799281 | 0.780895539 | 1.479318853 |
| ENSG00000147465 | -0.543220826 | 0.50710165 | 0.580874341 | 0.116690788 | 2.891530729 |
| ENSG00000168310 | 0.136722496 | 0.583678981 | 1.146509943 | 0.70309786 | 1.869562012 |
| ENSG00000171236 | -0.104421522 | 0.239521339 | 0.900845491 | 0.756973552 | 1.072062023 |
| ENSG00000087245 | 0.034082361 | 0.633222949 | 1.03466982 | 0.899512232 | 1.190135718 |
| ENSG00000100393 | 0.158709506 | 0.362211594 | 1.171997438 | 0.833030306 | 1.648893186 |
| ENSG00000149131 | 0.161519146 | 0.047106786 | 1.17529496 | 1.002066528 | 1.378469596 |
| ENSG00000028277 | 0.395396119 | 0.063492465 | 1.4849723 | 0.97803527 | 2.254665857 |
| ENSG00000130429 | 0.198218525 | 0.22350521 | 1.219228796 | 0.886085535 | 1.677624562 |
| ENSG00000058262 | 0.200515562 | 0.414502344 | 1.222032629 | 0.754946508 | 1.978105378 |
| ENSG00000105609 | 0.182047563 | 0.305906084 | 1.199671252 | 0.846667296 | 1.699854382 |
| ENSG00000005893 | 0.040719839 | 0.801983016 | 1.04156026 | 0.757658169 | 1.431843304 |
| ENSG00000131759 | 0.300391812 | 0.014569295 | 1.350387803 | 1.061184269 | 1.718407699 |
| ENSG00000185432 | 0.025466381 | 0.768288206 | 1.02579342 | 0.865929198 | 1.215171104 |
| ENSG00000108688 | -0.053307729 | 0.804748144 | 0.948088214 | 0.621291621 | 1.446778341 |
| ENSG00000163600 | -0.515732764 | 0.031382341 | 0.597062928 | 0.373289151 | 0.954981249 |
| ENSG00000234745 | 0.019936332 | 0.84347865 | 1.020136388 | 0.836973006 | 1.24338329 |
| ENSG00000115267 | 0.097073031 | 0.49897385 | 1.101940846 | 0.831659355 | 1.460061287 |
| ENSG00000012817 | 0.008161917 | 0.926773666 | 1.008195317 | 0.847131534 | 1.199881902 |
| ENSG00000115073 | 0.391517666 | 0.061577612 | 1.479224059 | 0.981195325 | 2.230038975 |
| ENSG00000140262 | -0.04570766 | 0.738525127 | 0.9553212 | 0.730458891 | 1.249404458 |
| ENSG00000182985 | 0.196630382 | 0.27629675 | 1.217294024 | 0.854390454 | 1.734341404 |
| ENSG00000140030 | -0.271523761 | 0.230779305 | 0.762217172 | 0.488892019 | 1.188350381 |
| ENSG00000169385 | 0.178225858 | 0.293015581 | 1.195095213 | 0.85729562 | 1.665997742 |
| ENSG00000196187 | -0.007786039 | 0.953712031 | 0.992244194 | 0.762854964 | 1.290610388 |
| ENSG00000204435 | 0.199051854 | 0.352923932 | 1.220245239 | 0.801773181 | 1.857131766 |
| ENSG00000179295 | -0.048151789 | 0.765684013 | 0.952989123 | 0.694319987 | 1.308025529 |
| ENSG00000280987 | -0.091405266 | 0.797986131 | 0.912647771 | 0.453237529 | 1.837725035 |
| ENSG00000198019 | 0.875466516 | 0.163118273 | 2.399994669 | 0.70127404 | 8.213585676 |
| ENSG00000156261 | -0.102975042 | 0.511420553 | 0.902149489 | 0.663421482 | 1.226782254 |
| ENSG00000167286 | -0.062453805 | 0.514899771 | 0.93945646 | 0.77847556 | 1.133726588 |
| ENSG00000131378 | 0.127133901 | 0.333409423 | 1.135569062 | 0.877677814 | 1.469237428 |
| ENSG00000133710 | 0.070084141 | 0.587536984 | 1.072598427 | 0.832634055 | 1.381720312 |
| ENSG00000196419 | -0.00527349 | 0.978070519 | 0.994740391 | 0.682981982 | 1.448806075 |
| ENSG00000239264 | -0.040829224 | 0.807293999 | 0.95999306 | 0.691490866 | 1.332753215 |
| ENSG00000035862 | 0.077757029 | 0.37433587 | 1.080860009 | 0.910472032 | 1.283134811 |
| ENSG00000101966 | -0.042892011 | 0.808419063 | 0.95801484 | 0.677322498 | 1.355030191 |
| ENSG00000203747 | 0.032427886 | 0.642643266 | 1.0329594 | 0.900731908 | 1.184597895 |
| ENSG00000127743 | 0.280737054 | 0.487094117 | 1.324105389 | 0.599872682 | 2.922711995 |
| ENSG00000108984 | -0.2192403 | 0.156939347 | 0.803128703 | 0.592844664 | 1.088001213 |
| ENSG00000167191 | 0.613146478 | 0.000111792 | 1.846231395 | 1.35268624 | 2.519852913 |
| ENSG00000165916 | 0.259625514 | 0.152354428 | 1.296444496 | 0.908551303 | 1.849943229 |
| ENSG00000175866 | 0.134648466 | 0.347381801 | 1.144134511 | 0.86398627 | 1.515121044 |
| ENSG00000169896 | 0.044675613 | 0.691189518 | 1.045688597 | 0.838826752 | 1.30356434 |
| ENSG00000127314 | -0.236597561 | 0.346082217 | 0.789308873 | 0.482507173 | 1.291190124 |
| ENSG00000069493 | -0.016989016 | 0.93764837 | 0.983154483 | 0.642330736 | 1.504820933 |
| ENSG00000100078 | -0.185354651 | 0.151912724 | 0.830809584 | 0.644742261 | 1.070574409 |
| ENSG00000123609 | -0.010276029 | 0.949190724 | 0.989776589 | 0.72155896 | 1.357695976 |
| ENSG00000078142 | -0.185098586 | 0.473793849 | 0.831022353 | 0.500795494 | 1.379002326 |
| ENSG00000135114 | 0.077082993 | 0.433608322 | 1.080131717 | 0.890600634 | 1.309997411 |
| ENSG00000126264 | 0.173549439 | 0.090930215 | 1.189519494 | 0.972716815 | 1.454643946 |
| ENSG00000135605 | -0.370061455 | 0.258859318 | 0.690691883 | 0.363330374 | 1.313006872 |
| ENSG00000162704 | -0.102860031 | 0.621365643 | 0.902253252 | 0.599876953 | 1.35704652 |
| ENSG00000043462 | -0.08975115 | 0.463839862 | 0.914158645 | 0.719004749 | 1.162281653 |
| ENSG00000065559 | -0.011116764 | 0.956670999 | 0.988944799 | 0.662228677 | 1.476849083 |
| ENSG00000174405 | 0.031738637 | 0.84422131 | 1.032247679 | 0.752135024 | 1.41668083 |
| ENSG00000095585 | -0.200409309 | 0.135102143 | 0.818395708 | 0.629219669 | 1.064447867 |
| ENSG00000007171 | -0.138060126 | 0.026246228 | 0.871046318 | 0.771198857 | 0.983821074 |
| ENSG00000205277 | -0.051401098 | 0.379298293 | 0.949897592 | 0.847055773 | 1.06522553 |
| ENSG00000065675 | -0.049690858 | 0.742251179 | 0.951523535 | 0.707632932 | 1.279472727 |
| ENSG00000128218 | 0.052431038 | 0.635041789 | 1.053829885 | 0.848678418 | 1.308572721 |
| ENSG00000051523 | 0.031845201 | 0.712132499 | 1.032357685 | 0.871704063 | 1.222619505 |
| ENSG00000174564 | 0.596343225 | 6.52E-05 | 1.81546789 | 1.354767812 | 2.432832866 |
| ENSG00000174516 | 0.151233661 | 0.479602194 | 1.163268437 | 0.764866207 | 1.769189752 |
| ENSG00000134061 | 0.148066969 | 0.464495017 | 1.15959055 | 0.779830223 | 1.724285883 |
| ENSG00000075785 | 0.493175336 | 0.117299438 | 1.63750761 | 0.88339132 | 3.035383201 |
| ENSG00000257017 | 0.185851011 | 0.483912403 | 1.204242828 | 0.71569261 | 2.026290014 |
| ENSG00000196126 | 0.015823053 | 0.818772862 | 1.015948901 | 0.887337242 | 1.163201677 |
| ENSG00000164307 | -0.20604703 | 0.119997075 | 0.813794802 | 0.627639051 | 1.05516376 |
| ENSG00000059804 | 0.140734182 | 0.114061762 | 1.151118619 | 0.966743095 | 1.370657916 |
| ENSG00000189171 | 0.208600098 | 0.117389142 | 1.23195224 | 0.948847585 | 1.599525936 |
| ENSG00000205403 | -0.086624831 | 0.408212188 | 0.917021069 | 0.74683421 | 1.125989719 |
| ENSG00000116329 | -0.024077511 | 0.934946501 | 0.97621004 | 0.547585755 | 1.740341187 |
| ENSG00000068878 | -0.226239407 | 0.249938853 | 0.797527145 | 0.542451776 | 1.172545793 |
| ENSG00000103381 | -0.201225312 | 0.216975736 | 0.817728167 | 0.594120042 | 1.125495367 |
| ENSG00000160185 | -0.114163331 | 0.690656134 | 0.892112235 | 0.508436928 | 1.565315568 |
| ENSG00000196776 | -0.192793103 | 0.257459434 | 0.824652575 | 0.590683097 | 1.151297324 |
| ENSG00000077238 | 0.333336124 | 0.114057841 | 1.39561632 | 0.923018665 | 2.110190168 |
| ENSG00000069424 | 0.22831446 | 0.106970893 | 1.256480377 | 0.951904358 | 1.658510042 |
| ENSG00000213186 | 0.000618101 | 0.997701116 | 1.000618292 | 0.65714732 | 1.523611125 |
| ENSG00000006210 | 0.269449365 | 0.00415155 | 1.309243337 | 1.0889437 | 1.57411087 |
| ENSG00000144648 | -0.02685858 | 0.941201716 | 0.973498904 | 0.476849672 | 1.987419038 |
| ENSG00000156886 | -0.724189036 | 0.460292752 | 0.484717498 | 0.070897242 | 3.313966031 |
| ENSG00000117983 | -0.017800605 | 0.714545597 | 0.98235689 | 0.892983169 | 1.080675529 |
| ENSG00000168995 | 0.091922972 | 0.665289633 | 1.096280375 | 0.722868478 | 1.662585515 |
| ENSG00000131508 | -0.669140858 | 0.006952755 | 0.512148397 | 0.315044674 | 0.832567576 |
| ENSG00000104312 | -0.175968746 | 0.237879739 | 0.838644194 | 0.626143675 | 1.123263097 |
| ENSG00000244482 | 0.029651302 | 0.912739005 | 1.030095279 | 0.606121335 | 1.750633452 |
| ENSG00000008394 | 0.085361628 | 0.490239656 | 1.089110848 | 0.854587969 | 1.387993375 |
| ENSG00000008438 | 0.075413526 | 0.818817526 | 1.078329977 | 0.565610808 | 2.055822702 |
| ENSG00000087086 | -0.00976849 | 0.941971949 | 0.990279067 | 0.76125127 | 1.288211486 |
| ENSG00000204257 | 0.087493957 | 0.31310198 | 1.091435669 | 0.920805747 | 1.293684171 |
| ENSG00000172322 | -0.060636315 | 0.809444022 | 0.941165465 | 0.574947849 | 1.540648311 |
| ENSG00000155093 | -0.003633989 | 0.967277822 | 0.996372606 | 0.837564328 | 1.185292085 |
| ENSG00000085449 | -0.049934935 | 0.77829949 | 0.951291319 | 0.671953376 | 1.346752923 |
| ENSG00000133048 | -0.060294134 | 0.38701724 | 0.941487569 | 0.821268109 | 1.07930508 |
| ENSG00000134242 | -0.075655179 | 0.677130668 | 0.927135847 | 0.649356047 | 1.323743549 |
| ENSG00000148218 | 0.690970466 | 0.002153604 | 1.995651305 | 1.283482948 | 3.102981726 |
| ENSG00000184009 | -0.294854737 | 0.089298431 | 0.744639748 | 0.529947841 | 1.046307414 |
| ENSG00000135926 | 0.157957966 | 0.34988298 | 1.171116967 | 0.840951357 | 1.630908777 |
| ENSG00000180871 | -0.314743983 | 0.113894201 | 0.729975736 | 0.494133674 | 1.078381426 |
| ENSG00000093072 | 0.016101277 | 0.859096978 | 1.016231601 | 0.850725288 | 1.213936722 |
| ENSG00000167925 | 0.242952795 | 0.152593848 | 1.275008436 | 0.913986581 | 1.7786328 |
| ENSG00000102245 | 0.095664165 | 0.69473684 | 1.100389452 | 0.682414731 | 1.774371056 |
| ENSG00000027075 | 0.122670572 | 0.555690489 | 1.130511937 | 0.751748671 | 1.700112403 |
| ENSG00000233276 | 0.070723121 | 0.562190086 | 1.073284015 | 0.844985104 | 1.363264951 |
| ENSG00000100448 | -0.000748772 | 0.994563215 | 0.999251508 | 0.805636806 | 1.239396673 |
| ENSG00000135148 | 0.281460017 | 0.230938353 | 1.325063015 | 0.836074052 | 2.100043641 |
| ENSG00000010438 | 0.046938276 | 0.655518028 | 1.048057317 | 0.852754947 | 1.28808885 |
| ENSG00000079246 | -0.198422339 | 0.353509714 | 0.820023452 | 0.539245015 | 1.246999867 |
| ENSG00000176903 | 0.283758134 | 0.015885137 | 1.328111667 | 1.054572921 | 1.672601834 |
| ENSG00000143226 | -0.033156875 | 0.740427726 | 0.967386789 | 0.795074087 | 1.177044019 |
| ENSG00000154188 | 0.041005874 | 0.88695696 | 1.041858225 | 0.591934041 | 1.833766073 |
| ENSG00000172057 | 0.153920934 | 0.302790418 | 1.166398661 | 0.870368336 | 1.56311504 |
| ENSG00000124635 | 0.013381659 | 0.883425511 | 1.013471594 | 0.847478657 | 1.211977039 |
| ENSG00000131355 | -0.955932046 | 0.024995122 | 0.384453649 | 0.16665897 | 0.886868602 |
| ENSG00000150991 | 0.113006613 | 0.546029079 | 1.119639337 | 0.775795557 | 1.615879639 |
| ENSG00000084207 | -0.074707732 | 0.410479264 | 0.928014675 | 0.776768961 | 1.108709644 |
| ENSG00000028137 | -0.040496136 | 0.766915867 | 0.960312875 | 0.734717602 | 1.255177248 |
| ENSG00000115919 | -0.13858582 | 0.637942425 | 0.870588534 | 0.488801779 | 1.550576181 |
| ENSG00000125931 | -0.064611114 | 0.661347407 | 0.937431947 | 0.702087131 | 1.25166609 |
| ENSG00000131408 | 0.463793523 | 0.021721017 | 1.590094618 | 1.070090744 | 2.36279111 |
| ENSG00000189403 | -0.104987892 | 0.380237493 | 0.900335423 | 0.712128101 | 1.138283792 |
| ENSG00000135018 | -0.117645216 | 0.620009992 | 0.889011404 | 0.558400622 | 1.415366038 |
| ENSG00000172724 | 0.026937253 | 0.684681243 | 1.027303341 | 0.902058841 | 1.169937154 |
| ENSG00000088888 | 0.200305166 | 0.196296662 | 1.221775546 | 0.90166097 | 1.655539649 |
| ENSG00000102001 | 1.18946176 | 0.014528726 | 3.285312446 | 1.265594285 | 8.528228992 |
| ENSG00000136436 | 0.074907806 | 0.717753854 | 1.077784781 | 0.718014017 | 1.617823618 |
| ENSG00000064012 | -0.347619492 | 0.097955675 | 0.706367603 | 0.467978719 | 1.066192053 |
| ENSG00000065427 | 0.05901489 | 0.782079318 | 1.060791036 | 0.698273838 | 1.611513364 |
| ENSG00000213809 | -0.169856682 | 0.797246993 | 0.843785737 | 0.230920103 | 3.083206536 |
| ENSG00000163599 | -0.332472828 | 0.062256239 | 0.717148155 | 0.505617487 | 1.017175017 |
| ENSG00000072786 | -0.142889507 | 0.531058952 | 0.866849845 | 0.554333835 | 1.355552568 |
| ENSG00000126804 | -0.059870595 | 0.774784291 | 0.941886411 | 0.62500892 | 1.419419759 |
| ENSG00000131196 | 0.549514609 | 0.00234712 | 1.732411917 | 1.215926647 | 2.468282982 |
| ENSG00000104921 | 0.165781995 | 0.334154939 | 1.180315759 | 0.843109723 | 1.652389068 |
| ENSG00000160856 | 0.249488112 | 0.437432326 | 1.283368307 | 0.683714118 | 2.408951591 |
| ENSG00000102158 | -0.182461084 | 0.298830666 | 0.833217068 | 0.590566427 | 1.175567476 |
| ENSG00000145782 | -0.099760069 | 0.608825995 | 0.905054543 | 0.61764981 | 1.32619441 |
| ENSG00000165240 | -0.056062064 | 0.781924646 | 0.945480454 | 0.635712311 | 1.406191564 |
| ENSG00000131507 | -0.370412914 | 0.083128176 | 0.690449176 | 0.454125579 | 1.049753827 |
| ENSG00000110651 | 0.339968539 | 0.054793363 | 1.40490339 | 0.993040402 | 1.987586337 |
| ENSG00000137077 | 0.029467728 | 0.5820127 | 1.029906198 | 0.927319325 | 1.143841985 |
| ENSG00000069869 | -0.175747697 | 0.282975467 | 0.838829596 | 0.608611601 | 1.15613158 |
| ENSG00000135829 | -0.186034609 | 0.462701803 | 0.830244861 | 0.505342154 | 1.364039243 |
| ENSG00000074603 | 0.052436802 | 0.783415324 | 1.053835959 | 0.725087409 | 1.531636345 |
| ENSG00000175582 | -0.151870446 | 0.373213036 | 0.859099573 | 0.614993449 | 1.200097461 |
| ENSG00000101347 | 0.005338469 | 0.96736385 | 1.005352744 | 0.778494496 | 1.298318927 |
| ENSG00000197766 | 0.085799774 | 0.106430817 | 1.089588143 | 0.981804974 | 1.209203815 |
| ENSG00000103415 | -0.13281776 | 0.46625037 | 0.875624652 | 0.612561338 | 1.25166001 |
| ENSG00000105647 | 0.253261777 | 0.667522883 | 1.288220459 | 0.405624113 | 4.091255671 |
| ENSG00000198746 | -0.020069864 | 0.928520453 | 0.980130195 | 0.632187735 | 1.519572661 |
| ENSG00000189013 | -0.133699786 | 0.635105353 | 0.874852669 | 0.503638562 | 1.519675515 |
| ENSG00000185499 | 0.019088756 | 0.762082457 | 1.019272111 | 0.900783388 | 1.153346798 |
| ENSG00000103569 | -0.101296257 | 0.307819672 | 0.903665276 | 0.743804466 | 1.097883877 |
| ENSG00000196843 | 0.156701458 | 0.303192769 | 1.169646373 | 0.867972735 | 1.57617006 |
| ENSG00000163251 | 0.012751271 | 0.940166431 | 1.012832915 | 0.725999157 | 1.412991329 |
| ENSG00000077150 | 0.359542252 | 0.02157965 | 1.432673461 | 1.054268152 | 1.946898655 |
| ENSG00000100324 | 0.404950126 | 0.11353854 | 1.499227725 | 0.907924073 | 2.475629668 |
| ENSG00000166825 | -0.004278254 | 0.926708759 | 0.995730885 | 0.908977119 | 1.090764524 |
| ENSG00000180900 | 0.030512194 | 0.799723138 | 1.030982462 | 0.814480108 | 1.30503474 |
| ENSG00000069399 | 0.085920622 | 0.540275 | 1.089719825 | 0.827731483 | 1.434631063 |
| ENSG00000108671 | -0.223757817 | 0.358906718 | 0.799508739 | 0.495704513 | 1.289506563 |
| ENSG00000120868 | -0.323003542 | 0.106675398 | 0.72397129 | 0.488991937 | 1.071867222 |
| ENSG00000100473 | 0.055250223 | 0.677641811 | 1.056805018 | 0.814438055 | 1.371297473 |
| ENSG00000062598 | 0.073740674 | 0.713662788 | 1.076527598 | 0.726051399 | 1.596184059 |
| ENSG00000115935 | 0.064326952 | 0.602413677 | 1.066441017 | 0.837195531 | 1.358459764 |
| ENSG00000150455 | 0.026407794 | 0.928212519 | 1.026759569 | 0.578055905 | 1.823759958 |
| ENSG00000102034 | 0.034265982 | 0.838357641 | 1.034859824 | 0.744562335 | 1.438341433 |
| ENSG00000067225 | 0.155559684 | 0.258348974 | 1.168311663 | 0.892094158 | 1.530053896 |
| ENSG00000204936 | -0.045849728 | 0.33035087 | 0.955185489 | 0.870951887 | 1.047565694 |
| ENSG00000131042 | 0.01413578 | 0.919781137 | 1.014236163 | 0.770304958 | 1.335412661 |
| ENSG00000117091 | -0.044094831 | 0.68017499 | 0.956863213 | 0.77588704 | 1.1800522 |
| ENSG00000145649 | -0.001618993 | 0.983578863 | 0.998382317 | 0.855739903 | 1.164801649 |
| ENSG00000179163 | -0.102066921 | 0.42698389 | 0.902969122 | 0.701944716 | 1.161563322 |
| ENSG00000112561 | 0.197016529 | 0.301040444 | 1.217764169 | 0.838318151 | 1.768957966 |
| ENSG00000169429 | -0.097274348 | 0.050187194 | 0.907307055 | 0.823140713 | 1.000079425 |
| ENSG00000035720 | 0.043979217 | 0.852987201 | 1.044960637 | 0.656272665 | 1.663855272 |
| ENSG00000138246 | 0.011754088 | 0.952300916 | 1.011823438 | 0.68840594 | 1.487184539 |
| ENSG00000275896 | -0.034625617 | 0.457530113 | 0.96596699 | 0.881637268 | 1.058362956 |
| ENSG00000178562 | -0.0532186 | 0.84569431 | 0.948172719 | 0.554780941 | 1.620516204 |
| ENSG00000112343 | 0.063640525 | 0.747381573 | 1.065709234 | 0.723527621 | 1.569720545 |
| ENSG00000182199 | -0.03557247 | 0.770562047 | 0.965052795 | 0.759847823 | 1.225675548 |
| ENSG00000067066 | 0.106109662 | 0.582589884 | 1.111943808 | 0.761632629 | 1.623379809 |
| ENSG00000197548 | -0.431614134 | 0.205204165 | 0.649459933 | 0.333082767 | 1.266346526 |
| ENSG00000101843 | -0.025923941 | 0.896242565 | 0.974409199 | 0.659981711 | 1.438635756 |
| ENSG00000215182 | -0.008059631 | 0.90965361 | 0.99197276 | 0.86306377 | 1.140135864 |
| ENSG00000205220 | 0.030569208 | 0.788437251 | 1.031041244 | 0.824722037 | 1.28897495 |
| ENSG00000163606 | -0.081626506 | 0.766912357 | 0.921616112 | 0.537214539 | 1.581074593 |
| ENSG00000112130 | 0.033558512 | 0.906217 | 1.034127951 | 0.591711813 | 1.807333563 |
| ENSG00000095261 | -0.036323238 | 0.859335063 | 0.964328536 | 0.645300969 | 1.441078766 |
| ENSG00000081237 | -0.075991446 | 0.416350328 | 0.926824134 | 0.771636198 | 1.113222757 |
| ENSG00000117595 | 0.043094213 | 0.839017737 | 1.044036252 | 0.688890578 | 1.582271161 |
| ENSG00000183134 | -0.012262779 | 0.94232382 | 0.987812103 | 0.708599745 | 1.377043609 |
| ENSG00000112851 | -0.022104574 | 0.87548805 | 0.978137942 | 0.741857475 | 1.289673375 |
| ENSG00000186074 | -0.053292307 | 0.72382034 | 0.948102835 | 0.705472125 | 1.274180727 |
| ENSG00000170581 | 0.146260501 | 0.348804203 | 1.157497678 | 0.852392671 | 1.571811818 |
| ENSG00000173762 | 0.046427702 | 0.627478946 | 1.047522343 | 0.8684141 | 1.263571213 |
| ENSG00000089041 | 0.249652936 | 0.227526615 | 1.283579855 | 0.855710099 | 1.925391842 |
| ENSG00000106927 | -0.225152049 | 0.20121173 | 0.798394814 | 0.565287724 | 1.127628024 |
| ENSG00000008516 | -0.123270883 | 0.527277298 | 0.884024163 | 0.6032291 | 1.295525564 |
| ENSG00000092098 | 0.262511976 | 0.305043789 | 1.300192039 | 0.787319198 | 2.147158794 |
| ENSG00000135077 | 0.071784334 | 0.54843832 | 1.074423602 | 0.849874127 | 1.358302414 |
| ENSG00000134070 | -0.074284641 | 0.54681945 | 0.928407393 | 0.729115405 | 1.182172647 |
| ENSG00000105939 | -0.032850048 | 0.873582967 | 0.967683654 | 0.645642656 | 1.45035593 |
| ENSG00000106952 | 0.159608999 | 0.543663675 | 1.173052117 | 0.700809806 | 1.963516003 |
| ENSG00000179344 | 0.043347776 | 0.530525964 | 1.044301015 | 0.912004078 | 1.19578918 |
| ENSG00000165699 | 0.503309703 | 0.034199344 | 1.654187088 | 1.038203456 | 2.635644205 |
| ENSG00000161643 | 0.896949629 | 0.06084554 | 2.45211184 | 0.959986137 | 6.263478446 |
| ENSG00000187889 | -0.291118526 | 0.661631959 | 0.747427083 | 0.202943951 | 2.752716907 |
| ENSG00000138496 | -0.094290727 | 0.492673003 | 0.910018157 | 0.695125497 | 1.191343218 |
| ENSG00000179954 | 0.164160145 | 0.103402546 | 1.178403015 | 0.967148049 | 1.435802583 |
| ENSG00000101871 | -0.170759874 | 0.367938476 | 0.843023981 | 0.581299598 | 1.222587176 |
| ENSG00000112679 | 0.602366813 | 0.010289509 | 1.826436522 | 1.15287258 | 2.893529108 |
| ENSG00000133246 | 0.213292351 | 0.418025272 | 1.237746454 | 0.738667538 | 2.074026821 |
| ENSG00000165799 | -0.302892995 | 0.316770983 | 0.738678134 | 0.408243741 | 1.336567673 |
| ENSG00000161905 | 0.142302918 | 0.37636801 | 1.152925837 | 0.841148014 | 1.58026645 |
| ENSG00000078902 | 0.402331786 | 0.074470032 | 1.495307373 | 0.961025346 | 2.326623483 |
| ENSG00000159128 | 0.10083587 | 0.631274576 | 1.106095084 | 0.732743214 | 1.669679517 |
| ENSG00000188822 | 0.546779925 | 0.351612285 | 1.727680789 | 0.546764003 | 5.459175975 |
| ENSG00000141012 | 0.594320795 | 0.005661276 | 1.811799944 | 1.189233573 | 2.76028117 |
| ENSG00000123843 | -0.00014833 | 0.998876105 | 0.999851681 | 0.81339376 | 1.229052192 |
| ENSG00000101096 | -0.027212417 | 0.850846825 | 0.973154505 | 0.732821063 | 1.292306865 |
| ENSG00000117090 | -0.235074816 | 0.360618132 | 0.790511704 | 0.477562077 | 1.308539318 |
| ENSG00000116260 | 0.065943699 | 0.553951534 | 1.068166577 | 0.858614696 | 1.328861293 |
| ENSG00000169299 | -0.458866606 | 0.005248157 | 0.631999544 | 0.457921445 | 0.872253152 |
| ENSG00000271605 | 0.259771258 | 0.132944862 | 1.296633458 | 0.92397346 | 1.819595905 |
| ENSG00000011422 | -0.03935288 | 0.737596655 | 0.961411386 | 0.763710518 | 1.210290852 |
| ENSG00000116815 | -0.433386147 | 0.012939737 | 0.6483101 | 0.460635921 | 0.912447264 |
| ENSG00000204389 | 0.248713262 | 0.000812156 | 1.282374274 | 1.108646692 | 1.483325383 |
| ENSG00000100911 | -0.03082537 | 0.815909553 | 0.969644887 | 0.74801172 | 1.256947161 |
| ENSG00000154016 | 0.37891685 | 0.220256557 | 1.460701574 | 0.796984163 | 2.677153685 |
| ENSG00000213413 | -0.187749593 | 0.714079919 | 0.828822224 | 0.303577804 | 2.262834337 |
| ENSG00000119922 | 0.102765944 | 0.376451622 | 1.10823199 | 0.882527602 | 1.391659752 |
| ENSG00000005339 | 0.249117649 | 0.241180315 | 1.282892956 | 0.845799448 | 1.945868303 |
| ENSG00000186141 | -0.094821322 | 0.682211185 | 0.909535434 | 0.577690705 | 1.432002796 |
| ENSG00000027697 | -0.243735468 | 0.103887271 | 0.783694919 | 0.58421936 | 1.051279311 |
| ENSG00000091317 | -0.157151676 | 0.338577066 | 0.854574431 | 0.619396637 | 1.179046533 |
| ENSG00000170315 | 0.232400978 | 0.197142912 | 1.261625512 | 0.886237215 | 1.796019062 |
| ENSG00000100365 | 0.010170606 | 0.923625993 | 1.010222502 | 0.820564352 | 1.243716597 |
| ENSG00000131966 | 0.066584203 | 0.735728824 | 1.068850961 | 0.726095827 | 1.573404409 |
| ENSG00000013503 | -0.077500745 | 0.789722322 | 0.925426335 | 0.523556123 | 1.635763318 |
| ENSG00000134827 | -0.061753093 | 0.215539824 | 0.940114979 | 0.85258601 | 1.036629927 |
| ENSG00000156127 | 0.151702336 | 0.089410762 | 1.163813759 | 0.976919212 | 1.386463128 |
| ENSG00000090432 | 0.04906586 | 0.80721502 | 1.05028952 | 0.708199432 | 1.557623498 |
| ENSG00000269335 | 0.405795158 | 0.120036198 | 1.500495157 | 0.899600516 | 2.502761697 |
| ENSG00000164342 | 0.015522472 | 0.916475166 | 1.015643571 | 0.759898085 | 1.357460801 |
| ENSG00000164713 | 0.125704836 | 0.45749312 | 1.133947418 | 0.813913562 | 1.579819784 |
| ENSG00000197971 | -0.027657682 | 0.903290755 | 0.97272129 | 0.622634996 | 1.519649093 |
| ENSG00000172794 | -0.005803901 | 0.975827369 | 0.994212909 | 0.683024722 | 1.447179402 |
| ENSG00000130038 | 0.081474945 | 0.648211204 | 1.084886036 | 0.764504693 | 1.539529739 |
| ENSG00000257335 | -0.483687762 | 0.272217747 | 0.616505668 | 0.25999074 | 1.461895292 |
| ENSG00000103196 | 0.152876733 | 0.118706156 | 1.165181341 | 0.961589494 | 1.411878526 |
| ENSG00000149534 | -0.533848009 | 0.068408775 | 0.586344365 | 0.33021094 | 1.041151797 |
| ENSG00000105298 | 0.386214743 | 0.104074259 | 1.47140061 | 0.923585139 | 2.344147457 |
| ENSG00000172543 | 0.050721951 | 0.652168233 | 1.052030337 | 0.843810229 | 1.311631207 |
| ENSG00000117228 | -0.044770183 | 0.646479207 | 0.956217212 | 0.789714025 | 1.157825906 |
| ENSG00000180448 | 0.334308213 | 0.034916452 | 1.396973643 | 1.023954883 | 1.905880222 |
| ENSG00000170515 | -0.081277645 | 0.634294298 | 0.921937685 | 0.659573866 | 1.288663998 |
| ENSG00000220205 | 0.219199305 | 0.143135041 | 1.245079403 | 0.92847146 | 1.669650372 |
| ENSG00000128394 | 0.456209746 | 0.035672006 | 1.578081306 | 1.031024385 | 2.415404178 |
| ENSG00000078401 | -0.02474324 | 0.786572952 | 0.975560364 | 0.815583823 | 1.16691626 |
| ENSG00000136026 | -0.010825378 | 0.937753012 | 0.989233005 | 0.753886002 | 1.298050284 |
| ENSG00000088986 | 0.204095869 | 0.476568038 | 1.226415723 | 0.699169304 | 2.151260815 |
| ENSG00000221887 | 0.549648499 | 0.123937732 | 1.732643884 | 0.860196515 | 3.489963954 |
| ENSG00000204713 | 0.122988799 | 0.600988096 | 1.130871754 | 0.713242141 | 1.793038926 |
| ENSG00000255690 | -0.04516047 | 0.731458203 | 0.955844086 | 0.738543437 | 1.237080814 |
| ENSG00000074966 | 0.469080201 | 0.157798219 | 1.598523197 | 0.833766955 | 3.064736971 |
| ENSG00000110944 | -0.140105645 | 0.241068055 | 0.869266397 | 0.687740646 | 1.098704973 |
| ENSG00000182511 | 0.481523017 | 0.002231421 | 1.618537587 | 1.188695762 | 2.203813628 |
| ENSG00000112146 | -0.085545366 | 0.731517188 | 0.918011495 | 0.563147816 | 1.496490053 |
| ENSG00000105851 | -0.006534582 | 0.973594724 | 0.993486722 | 0.674713066 | 1.462867574 |
| ENSG00000116473 | -0.063247235 | 0.717955964 | 0.938711362 | 0.666009971 | 1.323071815 |
| ENSG00000204616 | 0.006450037 | 0.936313858 | 1.006470884 | 0.859191496 | 1.178996352 |
| ENSG00000102103 | 0.095466482 | 0.47435963 | 1.100171946 | 0.84697901 | 1.429053491 |
| ENSG00000167535 | 0.323398284 | 0.154144196 | 1.381815597 | 0.885684948 | 2.155861796 |
| ENSG00000163563 | -0.070630107 | 0.508457886 | 0.931806497 | 0.755796834 | 1.14880522 |
| ENSG00000174885 | -0.173196864 | 0.255545909 | 0.840972042 | 0.623903905 | 1.133562347 |
| ENSG00000129226 | 0.114060706 | 0.607791014 | 1.120820163 | 0.725043098 | 1.732638849 |
| ENSG00000119801 | 0.089086275 | 0.630543482 | 1.093174966 | 0.760371318 | 1.57164201 |
| ENSG00000117984 | 0.336785194 | 0.014835388 | 1.400438209 | 1.068071876 | 1.836231457 |
| ENSG00000173110 | -0.02534551 | 0.810477999 | 0.974972991 | 0.792554085 | 1.199378503 |
| ENSG00000139572 | 0.057942241 | 0.746864624 | 1.05965379 | 0.745354831 | 1.506485377 |
| ENSG00000183813 | -0.41099384 | 0.088119432 | 0.662991015 | 0.413401562 | 1.063269051 |
| ENSG00000103855 | 0.177250862 | 0.286247818 | 1.193930567 | 0.861977245 | 1.653721379 |
| ENSG00000119401 | 0.649332068 | 0.032534374 | 1.914261806 | 1.055496056 | 3.471730889 |
| ENSG00000013374 | 0.201953488 | 0.390924848 | 1.223791086 | 0.771507766 | 1.941217818 |
| ENSG00000087088 | -0.0710403 | 0.613009392 | 0.931424355 | 0.707279417 | 1.226603388 |
| ENSG00000167618 | -0.159233192 | 0.332882867 | 0.852797471 | 0.617834972 | 1.177116154 |
| ENSG00000146094 | 0.166883933 | 0.348064818 | 1.18161711 | 0.83385774 | 1.674409108 |
| ENSG00000148444 | -0.09405551 | 0.657153111 | 0.910232234 | 0.600861465 | 1.378891423 |
| ENSG00000231389 | 0.038183535 | 0.582826471 | 1.038921894 | 0.906586059 | 1.190575005 |
| ENSG00000150337 | 0.274379208 | 0.113077181 | 1.315713637 | 0.937053962 | 1.847388139 |
| ENSG00000117115 | -0.006619749 | 0.912581052 | 0.993402114 | 0.882671621 | 1.118023663 |
| ENSG00000172578 | -0.048724089 | 0.806171013 | 0.952443883 | 0.645374807 | 1.405616304 |
| ENSG00000186716 | 0.123637214 | 0.539607299 | 1.131605266 | 0.762304156 | 1.679815683 |
| ENSG00000111640 | 0.045221673 | 0.733676356 | 1.046259762 | 0.806315747 | 1.357606489 |
| ENSG00000111203 | 0.588258046 | 0.003911656 | 1.800848685 | 1.207611677 | 2.685512279 |
| ENSG00000125148 | 0.070059048 | 0.306431209 | 1.072571513 | 0.937816289 | 1.226689773 |
| ENSG00000075651 | -0.163964035 | 0.349819582 | 0.848772547 | 0.601883882 | 1.19693326 |
| ENSG00000188372 | 0.094258193 | 0.402316288 | 1.098843423 | 0.881321961 | 1.370051946 |
| ENSG00000157404 | 0.089446337 | 0.448659244 | 1.093568647 | 0.867671895 | 1.378277196 |
| ENSG00000164761 | -0.05177994 | 0.552607787 | 0.949537799 | 0.800375471 | 1.126498829 |
| ENSG00000198898 | -0.174575266 | 0.244728905 | 0.839813643 | 0.625803878 | 1.127009562 |
| ENSG00000131871 | 0.164971017 | 0.434152035 | 1.179358936 | 0.780010834 | 1.783164335 |
| ENSG00000162676 | -0.157143638 | 0.280760381 | 0.8545813 | 0.642305151 | 1.137012831 |
| ENSG00000000938 | -0.004076925 | 0.972886174 | 0.995931374 | 0.787278201 | 1.259884118 |
| ENSG00000091106 | 0.032996064 | 0.91169027 | 1.033546471 | 0.576882807 | 1.851707652 |
| ENSG00000163636 | -0.644575509 | 0.025628642 | 0.524885304 | 0.298005698 | 0.924494346 |
| ENSG00000186832 | 0.107097025 | 0.205288046 | 1.113042242 | 0.943061824 | 1.313660462 |
| ENSG00000132256 | -0.017788189 | 0.938411889 | 0.982369087 | 0.625620697 | 1.54254651 |
| ENSG00000104974 | -0.306263684 | 0.542377097 | 0.736192471 | 0.27484111 | 1.971973388 |
| ENSG00000179914 | -0.071179652 | 0.042544633 | 0.931294568 | 0.869387659 | 0.997609713 |
| ENSG00000072062 | 0.438215628 | 0.098981943 | 1.549939081 | 0.920920083 | 2.608598945 |
| ENSG00000198821 | 0.027032672 | 0.878399237 | 1.027401369 | 0.72668323 | 1.45256355 |
| ENSG00000132170 | -0.093526292 | 0.441372 | 0.910714072 | 0.717755404 | 1.155547024 |
| ENSG00000125995 | -0.015915533 | 0.855786741 | 0.98421045 | 0.828983029 | 1.16850427 |
| ENSG00000060140 | 0.036394524 | 0.790149316 | 1.037064913 | 0.793221242 | 1.355868421 |
| ENSG00000121594 | -0.220200574 | 0.498360063 | 0.802357849 | 0.424166562 | 1.517748394 |
| ENSG00000092820 | 0.24817869 | 0.147135757 | 1.281688937 | 0.916357827 | 1.792669285 |
| ENSG00000114098 | 0.239409747 | 0.407121893 | 1.270499013 | 0.721346223 | 2.237715665 |
| ENSG00000101782 | 0.090160214 | 0.505435702 | 1.094349599 | 0.839300385 | 1.426903964 |
| ENSG00000226979 | -0.064510643 | 0.850931511 | 0.937526136 | 0.478400755 | 1.837278154 |
| ENSG00000213047 | -0.27750817 | 0.198737798 | 0.757669375 | 0.49622294 | 1.156864858 |
| ENSG00000096063 | -0.167151525 | 0.21996711 | 0.8460714 | 0.647758843 | 1.105097711 |
| ENSG00000163993 | -0.107506022 | 0.060284434 | 0.898071114 | 0.802790246 | 1.004660595 |
| ENSG00000114738 | -0.464513359 | 0.010326748 | 0.628440856 | 0.44065225 | 0.896257558 |
| ENSG00000104518 | 0.083301813 | 0.517056602 | 1.086869791 | 0.844763213 | 1.39836338 |
| ENSG00000080815 | 0.075149048 | 0.74518932 | 1.07804482 | 0.68518638 | 1.696152561 |
| ENSG00000163519 | 0.132465832 | 0.660074764 | 1.141640007 | 0.632640229 | 2.060162864 |
| ENSG00000118004 | 0.484609041 | 0.040031625 | 1.623540148 | 1.022301295 | 2.578381368 |
| ENSG00000100994 | 0.005874869 | 0.942223693 | 1.00589216 | 0.858129327 | 1.179098541 |
| ENSG00000119917 | 0.014542779 | 0.870436813 | 1.01464904 | 0.851962226 | 1.208401784 |
| ENSG00000116288 | -0.076001637 | 0.625285029 | 0.926814689 | 0.683167772 | 1.257356544 |
| ENSG00000188389 | 0.149328777 | 0.377216792 | 1.161054655 | 0.833503386 | 1.617327456 |
| ENSG00000109320 | -0.164008657 | 0.497129274 | 0.848734674 | 0.528655885 | 1.362607639 |
| ENSG00000185627 | 0.046762121 | 0.810893785 | 1.047872713 | 0.714421908 | 1.536959057 |
| ENSG00000183486 | 0.190331418 | 0.188807065 | 1.209650432 | 0.910699743 | 1.606736115 |
| ENSG00000169919 | 0.137031977 | 0.43030652 | 1.146864822 | 0.815858488 | 1.612165514 |
| ENSG00000166888 | 0.196163734 | 0.46184027 | 1.216726108 | 0.721554741 | 2.051711863 |
| ENSG00000276085 | -0.11498274 | 0.310154905 | 0.891381529 | 0.713882555 | 1.113013653 |
| ENSG00000115607 | -0.229960395 | 0.454094481 | 0.794565071 | 0.435164458 | 1.450793235 |
| ENSG00000097007 | 0.423425652 | 0.052608137 | 1.527184206 | 0.995239869 | 2.343446714 |
| ENSG00000197249 | -0.163478227 | 0.008679287 | 0.849184988 | 0.751588729 | 0.959454441 |
| ENSG00000168961 | 0.006342813 | 0.959041228 | 1.006362971 | 0.790000006 | 1.281982812 |
| ENSG00000125810 | 0.044133703 | 0.68753344 | 1.045122081 | 0.842878787 | 1.295892342 |
| ENSG00000167193 | -0.125311094 | 0.565208104 | 0.882222406 | 0.575590237 | 1.352205654 |
| ENSG00000182718 | -0.012175127 | 0.936150701 | 0.98789869 | 0.733406586 | 1.330699559 |
| ENSG00000080345 | -0.08456317 | 0.592813131 | 0.918913606 | 0.67401981 | 1.252785454 |
| ENSG00000129988 | 0.218864671 | 0.123556546 | 1.244662826 | 0.942063666 | 1.644459507 |
| ENSG00000204482 | -0.046013624 | 0.720348958 | 0.95502895 | 0.74235147 | 1.228636749 |
| ENSG00000213341 | -0.27434391 | 0.195660244 | 0.760070634 | 0.501638489 | 1.151640837 |
| ENSG00000115523 | 0.066951 | 0.480453867 | 1.069243084 | 0.887783155 | 1.287792819 |
| ENSG00000225697 | -0.11726763 | 0.441027414 | 0.889347145 | 0.659955036 | 1.198473081 |
| ENSG00000204632 | 0.071750689 | 0.462318851 | 1.074387455 | 0.887299993 | 1.300922362 |
| ENSG00000150782 | -0.12896478 | 0.274566154 | 0.879004924 | 0.697462127 | 1.107801594 |
| ENSG00000055208 | -0.092122745 | 0.636376846 | 0.9119932 | 0.622484973 | 1.33614727 |
| ENSG00000104432 | -0.410480883 | 0.010145721 | 0.663331189 | 0.485087252 | 0.907070354 |
| ENSG00000080561 | 0.566493126 | 0.001527551 | 1.762076823 | 1.241316219 | 2.501308436 |
| ENSG00000149177 | 0.015939995 | 0.925449869 | 1.016067714 | 0.727644128 | 1.418816644 |
| ENSG00000111405 | -0.176275814 | 0.858710207 | 0.838386713 | 0.120382941 | 5.838803046 |
| ENSG00000140992 | -0.334606932 | 0.141682213 | 0.715619318 | 0.458004491 | 1.118135344 |
| ENSG00000196664 | 0.085856861 | 0.721661946 | 1.089650346 | 0.679421865 | 1.747570894 |
| ENSG00000108405 | 0.149846174 | 0.436156581 | 1.161655536 | 0.796672066 | 1.693850759 |
| ENSG00000264522 | 0.068253719 | 0.784408693 | 1.070636915 | 0.656568589 | 1.745839539 |
| ENSG00000133789 | 0.237368368 | 0.222314037 | 1.267908089 | 0.866020962 | 1.856295623 |
| ENSG00000213281 | -0.170658487 | 0.237645988 | 0.843109457 | 0.635142422 | 1.119171909 |
| ENSG00000125538 | -0.078159553 | 0.285668203 | 0.924816858 | 0.801203926 | 1.067501285 |
| ENSG00000123610 | 0.032025854 | 0.734582345 | 1.032544201 | 0.858030939 | 1.242551378 |
| ENSG00000125818 | 0.325219229 | 0.07440187 | 1.384334098 | 0.968457211 | 1.978797695 |
| ENSG00000104689 | -0.271252447 | 0.081839762 | 0.762424 | 0.561706218 | 1.034865447 |
| ENSG00000120690 | -0.220927389 | 0.133035699 | 0.801774896 | 0.600993889 | 1.069633146 |
| ENSG00000111679 | 0.584913494 | 0.012301122 | 1.794835716 | 1.135382979 | 2.837311554 |
| ENSG00000103313 | -0.448751435 | 0.226313772 | 0.638424769 | 0.308604168 | 1.320741029 |
| ENSG00000108342 | 0.034863568 | 0.74329391 | 1.035478427 | 0.840477372 | 1.275722117 |
| ENSG00000122574 | 0.494019094 | 0.000818866 | 1.638889853 | 1.227120103 | 2.188832163 |
| ENSG00000197540 | 0.09539875 | 0.47279493 | 1.100097432 | 0.84786098 | 1.427373576 |
| ENSG00000108561 | -0.108096073 | 0.378468494 | 0.897541363 | 0.705637371 | 1.141635252 |
| ENSG00000163131 | -0.049453998 | 0.687947431 | 0.95174894 | 0.747677602 | 1.211519567 |
| ENSG00000142867 | -0.396696344 | 0.006021161 | 0.672538215 | 0.506731154 | 0.892598859 |
| ENSG00000172183 | 0.173477342 | 0.188554821 | 1.189433736 | 0.918410174 | 1.540436564 |
| ENSG00000152457 | 0.013848996 | 0.952234423 | 1.013945337 | 0.644493639 | 1.595182768 |
| ENSG00000126456 | 0.201496578 | 0.287279048 | 1.223232051 | 0.843971734 | 1.772922706 |
| ENSG00000254087 | -0.254487556 | 0.083749698 | 0.775313701 | 0.581052134 | 1.034522205 |
| ENSG00000109072 | -0.120855826 | 0.728020858 | 0.886161712 | 0.448432812 | 1.7511711 |
| ENSG00000197442 | -0.01948738 | 0.905486902 | 0.980701271 | 0.710936692 | 1.352827889 |
| ENSG00000198574 | -0.049221005 | 0.913841635 | 0.951970716 | 0.39028765 | 2.322000824 |
| ENSG00000026103 | -0.178034126 | 0.132217266 | 0.836913862 | 0.663766892 | 1.055227101 |
| ENSG00000135426 | 0.084589395 | 0.801765673 | 1.088270126 | 0.562270434 | 2.106338507 |
| ENSG00000162896 | 0.000846201 | 0.978616155 | 1.000846559 | 0.940794971 | 1.064731281 |
| ENSG00000187764 | 0.289618057 | 0.240825541 | 1.335917147 | 0.823382089 | 2.167492646 |
| ENSG00000023445 | -0.070824028 | 0.472966973 | 0.931625817 | 0.767783208 | 1.130431942 |
| ENSG00000096088 | -0.038771956 | 0.74731648 | 0.961970055 | 0.759846286 | 1.217859986 |
| ENSG00000117281 | -0.513930826 | 0.227944302 | 0.598139769 | 0.259399121 | 1.379230513 |
| ENSG00000105329 | 0.169319212 | 0.080108723 | 1.184498184 | 0.979896037 | 1.43182123 |
| ENSG00000105287 | 0.412667667 | 0.048283467 | 1.51084284 | 1.003118573 | 2.27554962 |
| ENSG00000072401 | -0.180279395 | 0.31366063 | 0.835036873 | 0.588036888 | 1.185787141 |
| ENSG00000112486 | -1.155351117 | 0.342563948 | 0.314946934 | 0.028978222 | 3.422969578 |
| ENSG00000112293 | 0.625961464 | 0.329245425 | 1.870043073 | 0.531772451 | 6.576235926 |
| ENSG00000127241 | 0.143389116 | 0.348550067 | 1.154178824 | 0.855204656 | 1.55767248 |
| ENSG00000095015 | -0.040419925 | 0.828803385 | 0.960386065 | 0.665787053 | 1.385339935 |
| ENSG00000139187 | 0.050462903 | 0.886682129 | 1.051757846 | 0.52540158 | 2.105426797 |
| ENSG00000213903 | 0.311911021 | 0.016937036 | 1.366033139 | 1.057508896 | 1.764568171 |
| ENSG00000180209 | 0.232897579 | 0.394757957 | 1.262252191 | 0.738242448 | 2.15820778 |
| ENSG00000115904 | -0.005212828 | 0.97669023 | 0.994800735 | 0.701253703 | 1.41122749 |
| ENSG00000179583 | -0.044909784 | 0.711647495 | 0.956083732 | 0.753493583 | 1.213143844 |
| ENSG00000168542 | 0.026401744 | 0.695935541 | 1.026753357 | 0.899419682 | 1.172114061 |
| ENSG00000135250 | -0.121802435 | 0.560213448 | 0.88532326 | 0.58765259 | 1.333776604 |
| ENSG00000115233 | -0.291053822 | 0.135762157 | 0.747475447 | 0.509942189 | 1.095652713 |
| ENSG00000013275 | 0.01643866 | 0.928209533 | 1.016574518 | 0.710942431 | 1.453596951 |
| ENSG00000125735 | -0.193470158 | 0.510336718 | 0.824094429 | 0.463254251 | 1.466001932 |
| ENSG00000086548 | -0.11747257 | 0.051029353 | 0.889164901 | 0.790198743 | 1.00052579 |
| ENSG00000186834 | 0.173409589 | 0.343149653 | 1.189353152 | 0.831000416 | 1.702238521 |
| ENSG00000187608 | 0.11473826 | 0.109996186 | 1.121579837 | 0.974363424 | 1.291039154 |
| ENSG00000069667 | -0.134178684 | 0.559095449 | 0.874433803 | 0.557466391 | 1.371624348 |
| ENSG00000174371 | -0.133085668 | 0.394489846 | 0.875390096 | 0.64441126 | 1.189159577 |
| ENSG00000130775 | 0.016631097 | 0.89894187 | 1.016770163 | 0.786598883 | 1.314293203 |
| ENSG00000141968 | 0.122314782 | 0.379301146 | 1.130109784 | 0.860397126 | 1.484370513 |
| ENSG00000204267 | 0.008897142 | 0.949133879 | 1.008936839 | 0.767627541 | 1.32610347 |
| ENSG00000162434 | -0.086798983 | 0.592418206 | 0.916861382 | 0.667252551 | 1.259845005 |
| ENSG00000197111 | -0.038035869 | 0.878753133 | 0.96267841 | 0.590538171 | 1.569330767 |
| ENSG00000177119 | 0.038773646 | 0.795053305 | 1.039535155 | 0.775853862 | 1.392831035 |
| ENSG00000165168 | -0.068480601 | 0.396152651 | 0.933811575 | 0.797189596 | 1.093847763 |
| ENSG00000145901 | 0.056666766 | 0.775671233 | 1.058303089 | 0.716710908 | 1.562701802 |
| ENSG00000100284 | 0.064456648 | 0.719057907 | 1.066579339 | 0.750705018 | 1.51536417 |
| ENSG00000130529 | 0.028711046 | 0.806100111 | 1.029127181 | 0.818286742 | 1.294292941 |
| ENSG00000110400 | -0.098620409 | 0.447420774 | 0.906086585 | 0.702545124 | 1.168598103 |
| ENSG00000158488 | -0.333033853 | 0.3037786 | 0.71674593 | 0.379931367 | 1.352151397 |
| ENSG00000174059 | 0.19225808 | 0.193407794 | 1.211983265 | 0.907123242 | 1.619298643 |
| ENSG00000104783 | -0.025523624 | 0.733405114 | 0.97479935 | 0.84164402 | 1.129021001 |
| ENSG00000101384 | 0.29485323 | 0.052116168 | 1.342929243 | 0.997302676 | 1.808336621 |
| ENSG00000143365 | -0.26516433 | 0.022192409 | 0.767079886 | 0.611154844 | 0.962786366 |
| ENSG00000086730 | 0.117387616 | 0.509897248 | 1.124555241 | 0.793150866 | 1.594431205 |
| ENSG00000170956 | -0.379490457 | 0.236990459 | 0.684209955 | 0.364778974 | 1.28336142 |
| ENSG00000205420 | 0.055746885 | 0.469708746 | 1.057330024 | 0.909022096 | 1.229834549 |
| ENSG00000090659 | 0.059894349 | 0.589700823 | 1.061724368 | 0.854028779 | 1.319930502 |
| ENSG00000151422 | -0.015683613 | 0.936854355 | 0.984438735 | 0.667852396 | 1.451098518 |
| ENSG00000153563 | -0.004743946 | 0.965956431 | 0.995267288 | 0.800438428 | 1.237518017 |
| ENSG00000196455 | 0.423488875 | 0.115254966 | 1.527280762 | 0.901669102 | 2.586965133 |
| ENSG00000136960 | 0.17358899 | 0.1275477 | 1.189566542 | 0.951533198 | 1.487145756 |
| ENSG00000138600 | 0.030226542 | 0.84371519 | 1.030688002 | 0.763165772 | 1.391988211 |
| ENSG00000164825 | -0.018619908 | 0.778276973 | 0.981552372 | 0.862230741 | 1.117386579 |
| ENSG00000189334 | 0.036472319 | 0.692138397 | 1.037145594 | 0.865831862 | 1.242355509 |
| ENSG00000118513 | -0.275650498 | 0.026763987 | 0.759078184 | 0.594776974 | 0.968765964 |
| ENSG00000111331 | -0.088294182 | 0.428203347 | 0.915491516 | 0.735855473 | 1.138980067 |
| ENSG00000155629 | 0.026675603 | 0.804864685 | 1.027034582 | 0.831146543 | 1.269090319 |
| ENSG00000142748 | 0.466302203 | 0.085734615 | 1.594088665 | 0.936534448 | 2.713321092 |
| ENSG00000079950 | -0.129548586 | 0.570285319 | 0.878491905 | 0.561656654 | 1.374056591 |
| ENSG00000111335 | 0.069993661 | 0.381964863 | 1.072501382 | 0.916752203 | 1.254711155 |
| ENSG00000129450 | 0.128291184 | 0.490119004 | 1.136883997 | 0.789733035 | 1.63663563 |
| ENSG00000181404 | 0.287576543 | 0.057230883 | 1.333192634 | 0.991203771 | 1.793175786 |
| ENSG00000183735 | -0.285276989 | 0.133236582 | 0.751805984 | 0.518057226 | 1.091022784 |
| ENSG00000198830 | -0.243240886 | 0.108190023 | 0.784082617 | 0.582735591 | 1.054999144 |
| ENSG00000160710 | 0.147997611 | 0.475265387 | 1.159510126 | 0.772365007 | 1.740710311 |
| ENSG00000126067 | -0.487493587 | 0.025702062 | 0.614163815 | 0.400186731 | 0.94255297 |
| ENSG00000170909 | 0.10054967 | 0.437675494 | 1.105778564 | 0.857811763 | 1.425424884 |
| ENSG00000117450 | -0.043769501 | 0.766359594 | 0.95717456 | 0.717144954 | 1.277542473 |
| ENSG00000136688 | -0.046894142 | 0.89138006 | 0.954188401 | 0.486778133 | 1.870411678 |
| ENSG00000162688 | -0.118980141 | 0.497167491 | 0.887825432 | 0.629741845 | 1.251677975 |
| ENSG00000102010 | 0.051114384 | 0.763282255 | 1.052443269 | 0.754631522 | 1.467785008 |
| ENSG00000174500 | -0.141661782 | 0.492511437 | 0.867914751 | 0.579138473 | 1.300683776 |
| ENSG00000143621 | -0.137304164 | 0.457870236 | 0.871705044 | 0.606644296 | 1.252578634 |
| ENSG00000073849 | -0.011021363 | 0.902989687 | 0.98903915 | 0.828408461 | 1.180816573 |
| ENSG00000159377 | 0.013414501 | 0.935664863 | 1.013504879 | 0.731758711 | 1.403730661 |
| ENSG00000121716 | 0.087676692 | 0.524930232 | 1.09163513 | 0.833086971 | 1.430423592 |
| ENSG00000196839 | 0.189542463 | 0.12845466 | 1.208696448 | 0.946648872 | 1.543282991 |
| ENSG00000165949 | -0.03753082 | 0.642207366 | 0.963164732 | 0.822132661 | 1.128390033 |
| ENSG00000135218 | 0.300295616 | 0.007346569 | 1.350257907 | 1.084087705 | 1.681779442 |
| ENSG00000197102 | 0.158008342 | 0.354161542 | 1.171175965 | 0.838422435 | 1.635992888 |
| ENSG00000165025 | -0.027283197 | 0.885248245 | 0.973085628 | 0.671790261 | 1.409510817 |
| ENSG00000183019 | -0.102663854 | 0.489714896 | 0.902430271 | 0.67438001 | 1.207598654 |
| ENSG00000180596 | -0.008790889 | 0.918592041 | 0.991247638 | 0.837471577 | 1.173259972 |
| ENSG00000182578 | 0.075901938 | 0.402856719 | 1.078856774 | 0.903089219 | 1.288833833 |
| ENSG00000160223 | 0.425145196 | 0.360379556 | 1.529812526 | 0.615150722 | 3.804476334 |
| ENSG00000023697 | -0.04144369 | 0.818111171 | 0.959403358 | 0.673917316 | 1.365827501 |
| ENSG00000239961 | 0.499220451 | 0.238335832 | 1.647436512 | 0.718513311 | 3.777309369 |
| ENSG00000198933 | 0.418265958 | 0.013934545 | 1.519324697 | 1.088582872 | 2.12050694 |
| ENSG00000109685 | -0.351934186 | 0.091931989 | 0.703326409 | 0.467092987 | 1.059035463 |
| ENSG00000109381 | -0.202193804 | 0.470694215 | 0.816936587 | 0.471625475 | 1.415074932 |
| ENSG00000167664 | -0.067495643 | 0.750514842 | 0.934731793 | 0.61659032 | 1.417024395 |
| ENSG00000127951 | -0.015110414 | 0.849527367 | 0.985003176 | 0.842642741 | 1.151414721 |
| ENSG00000174840 | -0.646660872 | 0.011726637 | 0.523791868 | 0.316777033 | 0.86609158 |
| ENSG00000203805 | 0.054021667 | 0.61345525 | 1.055507471 | 0.855917303 | 1.301639793 |
| ENSG00000084733 | -0.104242788 | 0.646985776 | 0.901006517 | 0.576728133 | 1.407617727 |
| ENSG00000241186 | -0.133183516 | 0.07846234 | 0.875304445 | 0.754631373 | 1.015274345 |
| ENSG00000047621 | -0.021327801 | 0.923021324 | 0.978898029 | 0.635127416 | 1.508738761 |
| ENSG00000103811 | -0.060108806 | 0.628872139 | 0.94166207 | 0.737960353 | 1.201592268 |
| ENSG00000100243 | 0.232864897 | 0.211300284 | 1.262210939 | 0.876112176 | 1.818461721 |
| ENSG00000140319 | 0.321189054 | 0.174059413 | 1.378766218 | 0.867673851 | 2.190911113 |
| ENSG00000100299 | 0.200252568 | 0.159566191 | 1.221711285 | 0.924230377 | 1.61494201 |
| ENSG00000106538 | 0.119671837 | 0.151795554 | 1.127126909 | 0.956970602 | 1.32753824 |
| ENSG00000100504 | 0.188104107 | 0.042931998 | 1.206959162 | 1.006002626 | 1.448058266 |
| ENSG00000102760 | 0.119413729 | 0.279226593 | 1.126836026 | 0.907661423 | 1.398935107 |
| ENSG00000101665 | -0.041186034 | 0.787384937 | 0.959650586 | 0.711425648 | 1.294484181 |
| ENSG00000126368 | 0.280417256 | 0.028610584 | 1.323682011 | 1.02975251 | 1.701509877 |
| ENSG00000171522 | 0.033987901 | 0.780577449 | 1.034572089 | 0.81452738 | 1.314061914 |
| ENSG00000109111 | 0.330564007 | 0.154924221 | 1.391752865 | 0.882545686 | 2.194760077 |
| ENSG00000076662 | -0.016059761 | 0.937171594 | 0.984068509 | 0.66008921 | 1.467060536 |
| ENSG00000184374 | -1.520857412 | 0.115391888 | 0.218524441 | 0.032904356 | 1.45126474 |
| ENSG00000131203 | 0.046712932 | 0.492622018 | 1.04782117 | 0.916931934 | 1.197394445 |
| ENSG00000175166 | 0.191402435 | 0.381575931 | 1.210946681 | 0.788728626 | 1.859184283 |
| ENSG00000140678 | -0.057569358 | 0.607019757 | 0.94405641 | 0.75809337 | 1.175636855 |
| ENSG00000243811 | 0.259783638 | 0.110346651 | 1.29664951 | 0.942597646 | 1.783687834 |
| ENSG00000070010 | -0.456128846 | 0.12084128 | 0.633732178 | 0.356138711 | 1.127696767 |
| ENSG00000138448 | -0.15290856 | 0.199130551 | 0.858208193 | 0.679559717 | 1.08382131 |
| ENSG00000203722 | -0.08183812 | 0.804933172 | 0.921421106 | 0.481275863 | 1.764096062 |
| ENSG00000196352 | -0.051294882 | 0.556911974 | 0.949998492 | 0.800563604 | 1.127327211 |
| ENSG00000163932 | 0.130939761 | 0.471339501 | 1.139899112 | 0.798235419 | 1.627802971 |
| ENSG00000176105 | -0.091594947 | 0.505389325 | 0.912474676 | 0.696884977 | 1.194759625 |
| ENSG00000115009 | -0.078770232 | 0.218708847 | 0.924252264 | 0.815224968 | 1.047860751 |
| ENSG00000197646 | -0.05900552 | 0.713725943 | 0.942701566 | 0.68780652 | 1.292058473 |
| ENSG00000122188 | -0.050417264 | 0.789635097 | 0.950832593 | 0.656512402 | 1.377099073 |
| ENSG00000124256 | -0.025062674 | 0.884882082 | 0.975248788 | 0.694653554 | 1.369186399 |
| ENSG00000148516 | 0.072595912 | 0.52732238 | 1.075295935 | 0.858555447 | 1.346752096 |
| ENSG00000184557 | 0.134734964 | 0.144376598 | 1.144233482 | 0.954872181 | 1.37114714 |
| ENSG00000070808 | -0.051431368 | 0.918458047 | 0.949868839 | 0.354846376 | 2.542651896 |
| ENSG00000136634 | -0.146656514 | 0.687639279 | 0.863590558 | 0.422495302 | 1.765199868 |
| ENSG00000123131 | -0.123209671 | 0.301474361 | 0.884078277 | 0.699829127 | 1.116836054 |
| ENSG00000126759 | 0.022619481 | 0.931217195 | 1.022877242 | 0.612006516 | 1.709586132 |
| ENSG00000168918 | 0.171398804 | 0.171932594 | 1.186964021 | 0.928185237 | 1.517890536 |
| ENSG00000132109 | -0.096426905 | 0.609748529 | 0.908076271 | 0.627075726 | 1.314996704 |
| ENSG00000130755 | 0.040787611 | 0.740998792 | 1.041630851 | 0.817853158 | 1.326637698 |
| ENSG00000159339 | -0.015469236 | 0.976381581 | 0.984649798 | 0.353606654 | 2.741846665 |
| ENSG00000130675 | 0.053785692 | 0.763933587 | 1.055258427 | 0.742870115 | 1.499010831 |
| ENSG00000133805 | -0.114715728 | 0.583422601 | 0.89161957 | 0.591725843 | 1.343503022 |
| ENSG00000107643 | -0.572807075 | 0.031734955 | 0.563940192 | 0.334360503 | 0.95115463 |
| ENSG00000204610 | 0.013388477 | 0.913259714 | 1.013478504 | 0.796511687 | 1.289546274 |
| ENSG00000169621 | -0.118585513 | 0.636845696 | 0.888175862 | 0.542867864 | 1.453127755 |
| ENSG00000118520 | -0.305098977 | 0.482716896 | 0.73705042 | 0.314428037 | 1.727719086 |
| ENSG00000136930 | 0.141096799 | 0.480141658 | 1.151536111 | 0.778358525 | 1.703630618 |
| ENSG00000124882 | -0.130133881 | 0.027610416 | 0.877977878 | 0.781982427 | 0.985757644 |
| ENSG00000123496 | -0.352317212 | 0.118186374 | 0.703057068 | 0.451908668 | 1.093781279 |
| ENSG00000274736 | 0.162591153 | 0.265500541 | 1.176555561 | 0.883730498 | 1.566408526 |
| ENSG00000164300 | -0.233781701 | 0.1542375 | 0.791534588 | 0.573847929 | 1.091799713 |
| ENSG00000173198 | -0.210124755 | 0.538274674 | 0.810483127 | 0.415066364 | 1.582597283 |
| ENSG00000178035 | -0.070313278 | 0.630487408 | 0.932101767 | 0.69991297 | 1.241316765 |
| ENSG00000118432 | 0.508361508 | 0.013195589 | 1.662564863 | 1.112208289 | 2.48525564 |
| ENSG00000068323 | 0.463579589 | 0.035245004 | 1.589754479 | 1.032569213 | 2.447602808 |
| ENSG00000166226 | -0.280334476 | 0.085450883 | 0.755530992 | 0.548922308 | 1.039905049 |
| ENSG00000171608 | 0.208069491 | 0.162027276 | 1.23129873 | 0.919817905 | 1.648257285 |
| ENSG00000163466 | -0.321676932 | 0.301042828 | 0.724932355 | 0.394039895 | 1.333689623 |
| ENSG00000124333 | 0.002364689 | 0.988975688 | 1.002367487 | 0.716726189 | 1.401847168 |
| ENSG00000049768 | -0.240025751 | 0.142397004 | 0.786607605 | 0.570794805 | 1.08401744 |
| ENSG00000115271 | 0.051340752 | 0.799800306 | 1.052681535 | 0.707908186 | 1.565370251 |
| ENSG00000215301 | -0.042150932 | 0.80567118 | 0.958725067 | 0.685256561 | 1.341327915 |
| ENSG00000129084 | -0.157544798 | 0.441581052 | 0.854238545 | 0.571890634 | 1.275984337 |
| ENSG00000050820 | 0.322621016 | 0.079395496 | 1.380741973 | 0.962865184 | 1.97997438 |
| ENSG00000092531 | -0.067751384 | 0.742597737 | 0.934492775 | 0.623698674 | 1.400158092 |
| ENSG00000151366 | -0.108837892 | 0.478592708 | 0.896875796 | 0.663720904 | 1.211934398 |
| ENSG00000104228 | -0.255878872 | 0.283121953 | 0.774235745 | 0.485232213 | 1.23536932 |
| ENSG00000105514 | -0.138601166 | 0.402367104 | 0.870575174 | 0.629391635 | 1.204180501 |
| ENSG00000116560 | -0.507110701 | 0.030826258 | 0.602233099 | 0.380067786 | 0.954263209 |
| ENSG00000150045 | -0.045106982 | 0.910919356 | 0.955895212 | 0.433736335 | 2.106661545 |
| ENSG00000142910 | -0.027302716 | 0.850690303 | 0.973066634 | 0.732286565 | 1.293016585 |
| ENSG00000204475 | 0.034762904 | 0.906536161 | 1.035374197 | 0.579521796 | 1.849800533 |
| ENSG00000261371 | 0.079266757 | 0.479237233 | 1.082493046 | 0.869085256 | 1.348304077 |
| ENSG00000118308 | 0.154148423 | 0.320846698 | 1.166664033 | 0.860542128 | 1.58168313 |
| ENSG00000006062 | 0.57293646 | 0.009543816 | 1.773467128 | 1.149922269 | 2.735128919 |
| ENSG00000078747 | -0.219479497 | 0.159783712 | 0.80293662 | 0.591272916 | 1.090371633 |
| ENSG00000125571 | -0.037125698 | 0.837483011 | 0.96355501 | 0.675785413 | 1.37386549 |
| ENSG00000101444 | -0.103837175 | 0.308765975 | 0.901372051 | 0.738014253 | 1.10088873 |
| ENSG00000105438 | -0.161802671 | 0.426968378 | 0.850609038 | 0.570631667 | 1.267955804 |
| ENSG00000171132 | 0.009750404 | 0.973165281 | 1.009798094 | 0.572147299 | 1.782219707 |
| ENSG00000136560 | -0.32574496 | 0.132728498 | 0.7219893 | 0.472174578 | 1.103974195 |
| ENSG00000173039 | 0.383655753 | 0.170807347 | 1.467640124 | 0.847581304 | 2.541310815 |
| ENSG00000148606 | 0.325957798 | 0.274448028 | 1.385356903 | 0.772120464 | 2.485640309 |
| ENSG00000244731 | 0.288915723 | 0.083032671 | 1.334979215 | 0.962933153 | 1.850771781 |
| ENSG00000204388 | 0.247004437 | 0.02266133 | 1.280184793 | 1.035192102 | 1.583158431 |
| ENSG00000103496 | 0.368127597 | 0.085473082 | 1.445026408 | 0.949885852 | 2.198265523 |
| ENSG00000101336 | -0.002479664 | 0.980340622 | 0.997523408 | 0.818970637 | 1.215004427 |
| ENSG00000135047 | 0.165638385 | 0.091501324 | 1.180146266 | 0.973613881 | 1.430490296 |
| ENSG00000076641 | -0.017446279 | 0.887609668 | 0.982705026 | 0.771520429 | 1.251696172 |
| ENSG00000137161 | 0.417427248 | 0.026199458 | 1.518050959 | 1.050674273 | 2.193333149 |
| ENSG00000171860 | 0.044801039 | 0.637621818 | 1.045819762 | 0.867952534 | 1.260136854 |
| ENSG00000204386 | 0.109962576 | 0.376330699 | 1.116236296 | 0.874892099 | 1.424156727 |
| ENSG00000137275 | 0.236053691 | 0.348555945 | 1.266242294 | 0.772979579 | 2.074271548 |
| ENSG00000100030 | -0.263818458 | 0.271835005 | 0.768112972 | 0.479802954 | 1.22966633 |
| ENSG00000104043 | -0.185992078 | 0.606768901 | 0.830280172 | 0.408911203 | 1.68585541 |
| ENSG00000185883 | 0.147977337 | 0.228309567 | 1.159486619 | 0.911406148 | 1.475093428 |
| ENSG00000164949 | 0.131020891 | 0.212467334 | 1.139991596 | 0.927797501 | 1.400716038 |
| ENSG00000204520 | 0.169212214 | 0.399358599 | 1.184371453 | 0.799065308 | 1.755470704 |
| ENSG00000010704 | 0.002825338 | 0.990829781 | 1.002829333 | 0.619416452 | 1.623571134 |
| ENSG00000158825 | -0.068246708 | 0.335165198 | 0.934030013 | 0.812989349 | 1.07309163 |
| ENSG00000160293 | 0.281193527 | 0.024028632 | 1.324709947 | 1.037663268 | 1.691161764 |
| ENSG00000130768 | -0.028803101 | 0.809097344 | 0.971607754 | 0.769145315 | 1.227364465 |
| ENSG00000125508 | 0.202657051 | 0.026772625 | 1.224652403 | 1.023593222 | 1.46520461 |
| ENSG00000113273 | -0.299143865 | 0.147284554 | 0.741452733 | 0.494740227 | 1.111193561 |
| ENSG00000139626 | 0.032273873 | 0.837023272 | 1.032800322 | 0.759392644 | 1.404644244 |
| ENSG00000158195 | -0.138917732 | 0.468470446 | 0.870299624 | 0.597809414 | 1.266994828 |
| ENSG00000173083 | -0.052333636 | 0.712145464 | 0.949012189 | 0.718690422 | 1.253146149 |
| ENSG00000133703 | -0.115349483 | 0.483902827 | 0.891054681 | 0.645129622 | 1.230727 |
| ENSG00000100519 | -0.058073154 | 0.75558312 | 0.943580918 | 0.654606088 | 1.360123233 |
| ENSG00000156508 | -0.000732237 | 0.996582729 | 0.999268031 | 0.714751418 | 1.397040386 |
| ENSG00000134760 | -0.582242614 | 0.447465509 | 0.558644137 | 0.124371096 | 2.509291006 |
| ENSG00000014216 | 0.067582264 | 0.674066267 | 1.069918272 | 0.780855241 | 1.465988891 |
| ENSG00000110801 | 0.061318275 | 0.852204248 | 1.063237263 | 0.55779949 | 2.026666386 |
| ENSG00000130202 | 0.030660484 | 0.841534847 | 1.031135357 | 0.763447428 | 1.392682831 |
| ENSG00000007264 | 0.271876221 | 0.138370945 | 1.312424541 | 0.916025101 | 1.880361328 |
| ENSG00000169228 | 0.145417272 | 0.416143887 | 1.156522053 | 0.814569136 | 1.64202546 |
| ENSG00000111540 | 0.131088906 | 0.57833352 | 1.140069136 | 0.718087574 | 1.810026632 |
| ENSG00000110442 | 0.178528073 | 0.496525683 | 1.195456443 | 0.714573976 | 1.999955435 |
| ENSG00000068079 | 0.176591769 | 0.182951566 | 1.193143915 | 0.920068224 | 1.547268305 |
| ENSG00000197081 | -0.10049633 | 0.550096138 | 0.904388431 | 0.650451952 | 1.257461727 |
| ENSG00000134321 | 0.072842121 | 0.469007449 | 1.075560715 | 0.883092522 | 1.309976955 |
| ENSG00000185896 | 0.067189088 | 0.680541988 | 1.069497688 | 0.776734267 | 1.472608269 |
| ENSG00000232629 | 0.022178082 | 0.803438174 | 1.022425844 | 0.858590367 | 1.217524265 |
| ENSG00000004468 | -0.017152368 | 0.916958627 | 0.982993896 | 0.712069782 | 1.356997621 |
| ENSG00000108861 | 0.196760164 | 0.409015462 | 1.217452016 | 0.763127273 | 1.942257163 |
| ENSG00000106100 | 0.308634117 | 0.163340793 | 1.361564106 | 0.882206298 | 2.101386964 |
| ENSG00000173757 | 0.025882995 | 0.909608242 | 1.026220868 | 0.656425337 | 1.604339763 |
| ENSG00000197471 | -0.054675828 | 0.742054961 | 0.946792022 | 0.683679326 | 1.311163141 |
| ENSG00000120889 | -0.300818579 | 0.032645669 | 0.74021205 | 0.561697391 | 0.975460966 |
| ENSG00000013563 | 0.265527374 | 0.168700587 | 1.304118553 | 0.893525556 | 1.903387304 |
| ENSG00000130150 | -0.188427424 | 0.335000443 | 0.828260613 | 0.564681156 | 1.214872564 |
| ENSG00000154079 | -0.222187255 | 0.128315767 | 0.800765403 | 0.601372822 | 1.066269053 |
| ENSG00000110031 | -0.135262385 | 0.369085229 | 0.873486692 | 0.65023426 | 1.173390958 |
| ENSG00000120509 | 0.007897244 | 0.965144352 | 1.007928509 | 0.707296148 | 1.436343011 |
| ENSG00000112299 | -0.103701083 | 0.209469272 | 0.901494729 | 0.766707266 | 1.059977885 |
| ENSG00000160796 | -0.057477572 | 0.653900982 | 0.944143066 | 0.734370721 | 1.213836694 |
| ENSG00000170458 | 0.133418247 | 0.109136145 | 1.142727841 | 0.970636637 | 1.345330342 |
| ENSG00000136573 | 0.199712867 | 0.405919963 | 1.221052104 | 0.762411976 | 1.955593941 |
| ENSG00000139370 | 0.037060043 | 0.886654275 | 1.037755329 | 0.62342348 | 1.727455183 |
| ENSG00000139946 | 0.165847052 | 0.241501694 | 1.180392549 | 0.894328083 | 1.557959094 |
| ENSG00000087191 | 0.142216995 | 0.490720645 | 1.152826779 | 0.769322614 | 1.727506196 |
| ENSG00000142541 | 0.102173334 | 0.490251195 | 1.107575436 | 0.828539335 | 1.480585524 |
| ENSG00000182149 | 0.548869064 | 0.083644523 | 1.731293928 | 0.929611735 | 3.224333936 |
| ENSG00000148180 | 0.141057833 | 0.122813059 | 1.151491241 | 0.962607533 | 1.377437877 |
| ENSG00000164163 | -0.178857286 | 0.164580198 | 0.836225232 | 0.649803704 | 1.076129044 |
| ENSG00000139200 | 1.014861535 | 0.000319163 | 2.75898135 | 1.587590611 | 4.794673159 |
| ENSG00000168214 | 0.021455339 | 0.906287877 | 1.02168716 | 0.714797297 | 1.460336597 |
| ENSG00000179639 | -0.110560109 | 0.51455266 | 0.895332511 | 0.642086438 | 1.248461668 |
| ENSG00000123570 | 0.227204332 | 0.334372253 | 1.255086296 | 0.79128705 | 1.990733464 |
| ENSG00000162711 | -0.027217457 | 0.907733381 | 0.973149601 | 0.61416301 | 1.541968712 |
| ENSG00000014257 | -0.018296202 | 0.897387445 | 0.981870157 | 0.743518918 | 1.296630096 |
| ENSG00000105369 | -0.024593419 | 0.704443816 | 0.975706535 | 0.85927536 | 1.107914048 |
| ENSG00000111012 | -0.121775715 | 0.56843962 | 0.885346917 | 0.582603845 | 1.345406779 |
| ENSG00000198851 | 0.007961507 | 0.939897479 | 1.007993284 | 0.819553868 | 1.239760435 |
| ENSG00000137312 | 0.598919395 | 0.006042724 | 1.820150873 | 1.186911135 | 2.791236094 |
| ENSG00000204472 | 0.079439072 | 0.420108194 | 1.082679592 | 0.892545025 | 1.31331761 |
| ENSG00000157764 | 0.076424852 | 0.700195638 | 1.079421071 | 0.73155491 | 1.592703202 |
| ENSG00000119655 | 0.348684253 | 0.064695972 | 1.417201642 | 0.978969228 | 2.051607382 |
| ENSG00000165102 | -0.071774677 | 0.689327334 | 0.93074059 | 0.654641091 | 1.323287 |
| ENSG00000159403 | 0.149108071 | 0.090141744 | 1.160798432 | 0.976926096 | 1.379278335 |
| ENSG00000111816 | -0.14629147 | 0.383530031 | 0.863905864 | 0.621681033 | 1.200508464 |
| ENSG00000118137 | 0.020600461 | 0.849207001 | 1.020814115 | 0.825504983 | 1.262332121 |
| ENSG00000001561 | -0.12598615 | 0.430531399 | 0.881627057 | 0.644529555 | 1.205943564 |
| ENSG00000099246 | -0.103504592 | 0.612351959 | 0.901671882 | 0.604196876 | 1.345608057 |
| ENSG00000172673 | -0.222520199 | 0.45810183 | 0.800498837 | 0.444716822 | 1.440913312 |
| ENSG00000134539 | -0.57522946 | 0.16630715 | 0.562575765 | 0.249138282 | 1.270344681 |
| ENSG00000154269 | -0.038082019 | 0.56826376 | 0.962633984 | 0.8446033 | 1.097159088 |
| ENSG00000057608 | -0.207946801 | 0.330087826 | 0.812250246 | 0.534500956 | 1.234329807 |
| ENSG00000168092 | -0.485806333 | 0.032303536 | 0.61520094 | 0.39431294 | 0.959826976 |
| ENSG00000213672 | 0.042010667 | 0.836459576 | 1.042905603 | 0.699860845 | 1.554097653 |
| ENSG00000113448 | -0.090044586 | 0.62413992 | 0.913890438 | 0.637486254 | 1.310139203 |
| ENSG00000123838 | -0.049855224 | 0.544646916 | 0.95136715 | 0.809650059 | 1.117889691 |
| ENSG00000137509 | 0.073258569 | 0.681609633 | 1.076008723 | 0.758268117 | 1.526893649 |
| ENSG00000120337 | -0.114259758 | 0.795125441 | 0.892026214 | 0.37654718 | 2.113176808 |
| ENSG00000104320 | -0.265199623 | 0.062006822 | 0.767052813 | 0.580586352 | 1.01340656 |
| ENSG00000175463 | 0.07368454 | 0.667260672 | 1.07646717 | 0.769323638 | 1.506234191 |
| ENSG00000186431 | -0.496013208 | 0.142525959 | 0.608953598 | 0.313814463 | 1.181667923 |
| ENSG00000110719 | 0.113295651 | 0.403667538 | 1.119963002 | 0.858463962 | 1.461117976 |
| ENSG00000101213 | 0.074334725 | 0.51939705 | 1.0771673 | 0.859159551 | 1.350493505 |
| ENSG00000183918 | -0.026592768 | 0.899223759 | 0.973757706 | 0.645226752 | 1.469567199 |
| ENSG00000198719 | 0.20678266 | 0.150567176 | 1.229715276 | 0.927604572 | 1.630220144 |
| ENSG00000137801 | 0.070061628 | 0.430868324 | 1.07257428 | 0.900986475 | 1.276840017 |
| ENSG00000187554 | 0.025240667 | 0.922272367 | 1.02556191 | 0.617683502 | 1.702776952 |
| ENSG00000188257 | -0.025250951 | 0.472929861 | 0.975065187 | 0.910094594 | 1.044673956 |
| ENSG00000123329 | 0.099942258 | 0.52906215 | 1.105107105 | 0.809562774 | 1.508544799 |
| ENSG00000089127 | -0.101635296 | 0.418848346 | 0.903358951 | 0.706068174 | 1.155777053 |
| ENSG00000112936 | 0.16516702 | 0.010054608 | 1.179590117 | 1.040184881 | 1.337678397 |
| ENSG00000057657 | -0.142284494 | 0.270039756 | 0.867374458 | 0.673597828 | 1.116895602 |
| ENSG00000243646 | 0.202007598 | 0.23980598 | 1.223857308 | 0.873875985 | 1.714003743 |
| ENSG00000149269 | -0.388230795 | 0.114673633 | 0.678255787 | 0.418709145 | 1.098688477 |
| ENSG00000136279 | 0.311547853 | 0.169823515 | 1.36553713 | 0.875235459 | 2.130502864 |
| ENSG00000008018 | -0.150419369 | 0.388556559 | 0.860347098 | 0.611194819 | 1.211065776 |
| ENSG00000171560 | -0.413188324 | 0.232367119 | 0.661537688 | 0.335786248 | 1.303305646 |
| ENSG00000140749 | -0.149541027 | 0.212614829 | 0.861103109 | 0.680659651 | 1.089382281 |
| ENSG00000119508 | 0.056727068 | 0.676303348 | 1.058366909 | 0.810931764 | 1.381300578 |
| ENSG00000160255 | 0.030043934 | 0.712521274 | 1.030499806 | 0.878302045 | 1.209071364 |
| ENSG00000099940 | -0.095170231 | 0.751376892 | 0.909218144 | 0.504634993 | 1.638169457 |
| ENSG00000109943 | -0.290973312 | 0.404065903 | 0.747535628 | 0.377392293 | 1.480712577 |
| ENSG00000112062 | -0.167379443 | 0.473316766 | 0.845878587 | 0.535335165 | 1.336565634 |
| ENSG00000154096 | 0.101035417 | 0.285874876 | 1.106315823 | 0.918954739 | 1.331877023 |
| ENSG00000113356 | -0.176094497 | 0.335010364 | 0.83853874 | 0.586198958 | 1.199502677 |
| ENSG00000107862 | 0.352199791 | 0.107377644 | 1.422192637 | 0.926322682 | 2.183506824 |
| ENSG00000079112 | -0.082707265 | 0.367884328 | 0.920620606 | 0.768946945 | 1.102211674 |
| ENSG00000143322 | 0.096617553 | 0.594018492 | 1.101439051 | 0.772090927 | 1.57127605 |
| ENSG00000077264 | 1.164575463 | 0.044679893 | 3.204562138 | 1.02805061 | 9.989020379 |
| ENSG00000140835 | 0.041482523 | 0.630898468 | 1.042354944 | 0.880083724 | 1.234545987 |
| ENSG00000142515 | -0.030036882 | 0.903525121 | 0.970409742 | 0.597061626 | 1.577215863 |
| ENSG00000136167 | -0.004594616 | 0.954374081 | 0.995415923 | 0.850450669 | 1.165091516 |
| ENSG00000158270 | 0.18178807 | 0.158904934 | 1.199359987 | 0.93134334 | 1.544504928 |
| ENSG00000100528 | -0.184528095 | 0.267254924 | 0.831496579 | 0.600177489 | 1.151970164 |
| ENSG00000010671 | 0.086073916 | 0.617950516 | 1.089886885 | 0.777112272 | 1.528548017 |
| ENSG00000116030 | 0.065796839 | 0.698613917 | 1.068009717 | 0.765469234 | 1.490124887 |
| ENSG00000170542 | 0.024843362 | 0.860029217 | 1.02515453 | 0.777798656 | 1.351174629 |
| ENSG00000163823 | -0.022578511 | 0.840000076 | 0.977674476 | 0.785237705 | 1.217271375 |
| ENSG00000160209 | 0.056299907 | 0.746770786 | 1.057914913 | 0.751688355 | 1.488893575 |
| ENSG00000068650 | -0.079723584 | 0.485041007 | 0.923371546 | 0.738218596 | 1.154962792 |
| ENSG00000168495 | -0.295839555 | 0.18440024 | 0.743906774 | 0.480617623 | 1.151429459 |
| ENSG00000177606 | 0.039807064 | 0.740860885 | 1.040609984 | 0.821923189 | 1.31748216 |
| ENSG00000078081 | 0.035476219 | 0.792503713 | 1.036113008 | 0.795453153 | 1.349583142 |
| ENSG00000137033 | -0.094308037 | 0.153225892 | 0.910002404 | 0.799533146 | 1.03573489 |
| ENSG00000137251 | -0.127373001 | 0.15738326 | 0.880405219 | 0.737908478 | 1.050419357 |
| ENSG00000126262 | -0.068046789 | 0.64926583 | 0.934216761 | 0.696765536 | 1.252589159 |
| ENSG00000183087 | 0.077684547 | 0.505394392 | 1.080781669 | 0.859913506 | 1.358379661 |
| ENSG00000101160 | 0.190210887 | 0.213942949 | 1.209504639 | 0.896046411 | 1.63261797 |
| ENSG00000106299 | -0.049538716 | 0.766083104 | 0.951668312 | 0.686668944 | 1.318936271 |
| ENSG00000120899 | -0.086655199 | 0.561343732 | 0.916993221 | 0.68450609 | 1.228442786 |
| ENSG00000135677 | 0.007276163 | 0.969212369 | 1.007302698 | 0.696131252 | 1.457568129 |
| ENSG00000143537 | 0.003461417 | 0.980132397 | 1.003467415 | 0.764168789 | 1.317702146 |
| ENSG00000171791 | 0.151983965 | 0.332678958 | 1.16414157 | 0.855975092 | 1.58325354 |
| ENSG00000134830 | 0.431268048 | 0.289615164 | 1.539208076 | 0.692854825 | 3.419419795 |
| ENSG00000180644 | 0.000232625 | 0.998161721 | 1.000232652 | 0.820647621 | 1.21911687 |
| ENSG00000075711 | -0.056108244 | 0.767665542 | 0.945436792 | 0.651585931 | 1.3718079 |
| ENSG00000276231 | 0.296004083 | 0.33427421 | 1.344475646 | 0.7372275 | 2.451909029 |
| ENSG00000115594 | -0.007954868 | 0.942390934 | 0.992076688 | 0.799550419 | 1.230961965 |
| ENSG00000083799 | -0.053550772 | 0.784271361 | 0.947857815 | 0.646005248 | 1.390754085 |
| ENSG00000164520 | -0.708329481 | 0.074515357 | 0.492466185 | 0.226101606 | 1.072628134 |
| ENSG00000136238 | -0.080875854 | 0.742747407 | 0.922308185 | 0.569025723 | 1.494927827 |
| ENSG00000197272 | 0.870202368 | 0.032217454 | 2.387393936 | 1.076637413 | 5.293936227 |
| ENSG00000067900 | 0.041364867 | 0.792738303 | 1.042232312 | 0.765530909 | 1.418947529 |
| ENSG00000173372 | 0.147422754 | 0.045398003 | 1.158843765 | 1.003024114 | 1.338869975 |
| ENSG00000012504 | 0.057159262 | 0.650207581 | 1.058824427 | 0.827050618 | 1.355550848 |
| ENSG00000169230 | -0.144043287 | 0.29544035 | 0.865850267 | 0.661080332 | 1.134047782 |
| ENSG00000165280 | 0.069504007 | 0.74300076 | 1.071976357 | 0.707536228 | 1.624133528 |
| ENSG00000159674 | 0.12280741 | 0.229762556 | 1.130666645 | 0.925323185 | 1.381578979 |
| ENSG00000154822 | 0.038854601 | 0.751843995 | 1.039619313 | 0.817112336 | 1.322716923 |
| ENSG00000067606 | -0.072042972 | 0.729361535 | 0.93049091 | 0.618678388 | 1.399456245 |
| ENSG00000177189 | -0.126616661 | 0.421558635 | 0.881071357 | 0.647014897 | 1.199797314 |
| ENSG00000091831 | 0.133314775 | 0.81571477 | 1.142609607 | 0.372386937 | 3.505914373 |
| ENSG00000101307 | -0.106404139 | 0.730448516 | 0.899061229 | 0.490800105 | 1.646925265 |
| ENSG00000100097 | 0.093504947 | 0.279115202 | 1.098016035 | 0.926979283 | 1.300610741 |
| ENSG00000104774 | 0.270597166 | 0.128553766 | 1.310746951 | 0.924628328 | 1.858106136 |
| ENSG00000166523 | -0.107071297 | 0.504145163 | 0.898461613 | 0.656235645 | 1.230096652 |
| ENSG00000106003 | 0.001198172 | 0.991673686 | 1.00119889 | 0.799447031 | 1.253865706 |
| ENSG00000144381 | -0.117646237 | 0.362203716 | 0.889010496 | 0.690246149 | 1.145011332 |
| ENSG00000001036 | -0.248813241 | 0.257914359 | 0.77972558 | 0.50668481 | 1.199901732 |
| ENSG00000139178 | 0.509743452 | 0.017130488 | 1.664864022 | 1.094883885 | 2.531567275 |
| ENSG00000169248 | -0.089675647 | 0.161233039 | 0.91422767 | 0.806432311 | 1.036431975 |
| ENSG00000197694 | 0.185741057 | 0.332327453 | 1.204110424 | 0.827139407 | 1.752886998 |
| ENSG00000147234 | -0.336885346 | 0.411941425 | 0.713990697 | 0.319296314 | 1.5965819 |
| ENSG00000140853 | -0.091907518 | 0.471917114 | 0.912189507 | 0.710123022 | 1.171754288 |
| ENSG00000067369 | 0.231500116 | 0.214968472 | 1.260489473 | 0.874234025 | 1.817400909 |
| ENSG00000166349 | 0.545364535 | 0.100566595 | 1.725237178 | 0.899818714 | 3.307825536 |
| ENSG00000174125 | -0.117445229 | 0.502920007 | 0.889189212 | 0.630614784 | 1.253788326 |
| ENSG00000110852 | 0.168989334 | 0.197277361 | 1.184107509 | 0.915857124 | 1.530927211 |
| ENSG00000204264 | -0.028540822 | 0.816691697 | 0.97186262 | 0.76348413 | 1.237114061 |
| ENSG00000041357 | -0.047482724 | 0.78423589 | 0.953626948 | 0.678834383 | 1.339655707 |
| ENSG00000033800 | 0.092783814 | 0.681684327 | 1.097224505 | 0.704279043 | 1.709409964 |
| ENSG00000090674 | 0.32691964 | 0.081016502 | 1.386690038 | 0.960490692 | 2.00200718 |
| ENSG00000160307 | 0.033562477 | 0.767419217 | 1.034132051 | 0.827904429 | 1.291730135 |
| ENSG00000137265 | -0.091191506 | 0.501885564 | 0.912842879 | 0.699527885 | 1.19120644 |
| ENSG00000111729 | -0.258812224 | 0.077853195 | 0.771967967 | 0.578976483 | 1.029289719 |
| ENSG00000026751 | -0.033060819 | 0.715520495 | 0.967479717 | 0.809890236 | 1.155733161 |
| ENSG00000184371 | 0.155535246 | 0.231028484 | 1.168283112 | 0.905755471 | 1.506902772 |
| ENSG00000206503 | -0.011194437 | 0.917075987 | 0.988867987 | 0.800976021 | 1.220835419 |
| ENSG00000185475 | 0.094553435 | 0.601244728 | 1.099167896 | 0.77101076 | 1.566995076 |
| ENSG00000169403 | -0.07319743 | 0.522605694 | 0.929417317 | 0.742602803 | 1.163228237 |
| ENSG00000234127 | 0.186686831 | 0.457950517 | 1.205249779 | 0.736173769 | 1.973212156 |
| ENSG00000205809 | -0.368452034 | 0.33102727 | 0.691804392 | 0.329107203 | 1.454217086 |
| ENSG00000141574 | 0.023442466 | 0.719825752 | 1.023719401 | 0.900637965 | 1.163621181 |
| ENSG00000143546 | 0.035876197 | 0.603608808 | 1.036527513 | 0.905244075 | 1.186850392 |
| ENSG00000129353 | 0.175079089 | 0.412261142 | 1.191340434 | 0.783930223 | 1.810482603 |
| ENSG00000105373 | 0.26878598 | 0.073819277 | 1.308375093 | 0.974437258 | 1.756752802 |
| ENSG00000168040 | 0.110745499 | 0.570678966 | 1.117110565 | 0.761826515 | 1.638084248 |
| ENSG00000221823 | -0.001843291 | 0.993776949 | 0.998158406 | 0.628104009 | 1.586234429 |
| ENSG00000272398 | -0.170443717 | 0.030969082 | 0.843290552 | 0.722322078 | 0.984517816 |
| ENSG00000100784 | -0.517424846 | 0.144608691 | 0.596053503 | 0.297426321 | 1.194513577 |
| ENSG00000121807 | -0.065438456 | 0.707005941 | 0.936656691 | 0.665873055 | 1.317557077 |
| ENSG00000163739 | -0.154483644 | 0.009514397 | 0.856857507 | 0.762423595 | 0.962988019 |
| ENSG00000163357 | 0.290297426 | 0.624662574 | 1.336825035 | 0.417845399 | 4.27694353 |
| ENSG00000163347 | -0.009848383 | 0.88356703 | 0.990199954 | 0.867923352 | 1.129703384 |
| ENSG00000153395 | 0.115184819 | 0.340984653 | 1.1220808 | 0.885236115 | 1.422293218 |
| ENSG00000075223 | -0.017930308 | 0.869957326 | 0.982229484 | 0.792477252 | 1.217416343 |
| ENSG00000204287 | -0.036728143 | 0.573989296 | 0.963938153 | 0.848084976 | 1.095617526 |
| ENSG00000157212 | -0.241900573 | 0.402157641 | 0.785134237 | 0.445826878 | 1.38267969 |
| ENSG00000116062 | -0.14099957 | 0.484005306 | 0.868489685 | 0.585164915 | 1.288994458 |
| ENSG00000159958 | 0.257736707 | 0.142460332 | 1.293998073 | 0.916969209 | 1.826049333 |
| ENSG00000132465 | -0.02752796 | 0.400068895 | 0.972847481 | 0.912429615 | 1.037266005 |
| ENSG00000131142 | -0.084884451 | 0.347633928 | 0.918618424 | 0.769490078 | 1.096648069 |
| ENSG00000141293 | 0.050612262 | 0.624169423 | 1.051914947 | 0.859113867 | 1.287984164 |
| ENSG00000161921 | -0.052404541 | 0.671959478 | 0.948944902 | 0.744564161 | 1.209427574 |
| ENSG00000133063 | -0.001697598 | 0.990523035 | 0.998303842 | 0.754411559 | 1.321043599 |
| ENSG00000147140 | -0.052547133 | 0.787008507 | 0.9488096 | 0.648096989 | 1.389050824 |
| ENSG00000125726 | 0.01682617 | 0.88974611 | 1.016968527 | 0.801659307 | 1.29010538 |
| ENSG00000026950 | 0.004692154 | 0.974502703 | 1.004703179 | 0.753487706 | 1.339674781 |
| ENSG00000164251 | -0.199175852 | 0.166975003 | 0.819405786 | 0.617762677 | 1.08686696 |
| ENSG00000260314 | -0.04394803 | 0.60602802 | 0.957003691 | 0.809806627 | 1.130956495 |
| ENSG00000185745 | 0.116171661 | 0.231888268 | 1.123188663 | 0.928407339 | 1.358835417 |
| ENSG00000172575 | -0.128869699 | 0.536733862 | 0.879088504 | 0.584070117 | 1.323122988 |
| ENSG00000166747 | -0.016108441 | 0.939209496 | 0.984020606 | 0.650448003 | 1.488660966 |
| ENSG00000169413 | 0.054109389 | 0.667518653 | 1.055600066 | 0.82466408 | 1.351206543 |
| ENSG00000130303 | 0.134327598 | 0.028946276 | 1.143767454 | 1.013886944 | 1.29028586 |
| ENSG00000181143 | 0.495382703 | 0.082413089 | 1.641126183 | 0.938347121 | 2.870254608 |
| ENSG00000160213 | 0.125188998 | 0.373983018 | 1.133362636 | 0.860018326 | 1.493585458 |
| ENSG00000128829 | 0.187402749 | 0.467901863 | 1.206112948 | 0.727169646 | 2.000507656 |
| ENSG00000141959 | 0.207771826 | 0.150004442 | 1.230932271 | 0.927633421 | 1.633397655 |
| ENSG00000081277 | 0.04900444 | 0.61091572 | 1.050225014 | 0.869550047 | 1.268440596 |
| ENSG00000112149 | 0.046213272 | 0.757786928 | 1.047297746 | 0.780753691 | 1.404838148 |
| ENSG00000168067 | 0.307304733 | 0.143969703 | 1.359755267 | 0.900410625 | 2.053434661 |
| ENSG00000169871 | 0.37612176 | 0.038849901 | 1.456624482 | 1.019451779 | 2.081270468 |
| ENSG00000149357 | 0.057966312 | 0.770245455 | 1.059679297 | 0.71817375 | 1.563577355 |
| ENSG00000146425 | -0.21116786 | 0.300335628 | 0.80963815 | 0.542928507 | 1.207366945 |
| ENSG00000145555 | -0.05905195 | 0.728346313 | 0.942657797 | 0.675513227 | 1.315449774 |
| ENSG00000176919 | 0.227672741 | 0.010178607 | 1.255674328 | 1.055507931 | 1.493800256 |
| ENSG00000145335 | 0.278224824 | 0.198160612 | 1.320783107 | 0.864551322 | 2.017772655 |
| ENSG00000021355 | -0.099848952 | 0.439155318 | 0.904974102 | 0.702706582 | 1.165462437 |
| ENSG00000276600 | 0.202561114 | 0.378942361 | 1.224534919 | 0.779838801 | 1.922815028 |
| ENSG00000254709 | -0.008887711 | 0.855531899 | 0.991151668 | 0.900715602 | 1.090667938 |
| ENSG00000086062 | -0.087832147 | 0.640484345 | 0.915914603 | 0.633535054 | 1.324156499 |
| ENSG00000137845 | 0.041728145 | 0.818682623 | 1.042611001 | 0.729758914 | 1.489584682 |
| ENSG00000170345 | -0.012046273 | 0.87020652 | 0.988025993 | 0.855094484 | 1.141622804 |
| ENSG00000131015 | 0.144310183 | 0.16740417 | 1.15524239 | 0.941236991 | 1.417905364 |
| ENSG00000106348 | 0.099732676 | 0.416587499 | 1.104875519 | 0.86858354 | 1.405449053 |
| ENSG00000055163 | -0.153463436 | 0.338273755 | 0.857732125 | 0.626522788 | 1.174265985 |
| ENSG00000115604 | -0.167096099 | 0.477759434 | 0.846118297 | 0.53343133 | 1.34209622 |
| ENSG00000152207 | -0.300385385 | 0.576734664 | 0.740532775 | 0.25790103 | 2.126353634 |
| ENSG00000162747 | -0.112878419 | 0.354051676 | 0.893259257 | 0.703561291 | 1.134104604 |
| ENSG00000092969 | 0.360100331 | 0.039854627 | 1.43347323 | 1.01683562 | 2.020823679 |
| ENSG00000135932 | -0.004154456 | 0.984100789 | 0.995854162 | 0.661825008 | 1.498470894 |
| ENSG00000125657 | -0.132442341 | 0.079288648 | 0.875953439 | 0.7555065 | 1.015602681 |
| ENSG00000204592 | 0.123985037 | 0.401160501 | 1.131998933 | 0.847501237 | 1.511999663 |
| ENSG00000169398 | -0.235585036 | 0.271374991 | 0.790108473 | 0.519242339 | 1.202273681 |
| ENSG00000162594 | -0.317496576 | 0.409156213 | 0.727969173 | 0.342518067 | 1.54718588 |
| ENSG00000169876 | -0.014549078 | 0.798507315 | 0.985556248 | 0.881391964 | 1.10203083 |
| ENSG00000164816 | -0.048122573 | 0.177663413 | 0.953016966 | 0.888613758 | 1.022087863 |
| ENSG00000109270 | -0.038057899 | 0.845446447 | 0.962657202 | 0.656576772 | 1.411425029 |
| ENSG00000141480 | 0.05466256 | 0.763099458 | 1.056184155 | 0.740236722 | 1.506984099 |
| ENSG00000232810 | -0.147420175 | 0.533998712 | 0.862931319 | 0.542256758 | 1.373243303 |
| ENSG00000140284 | 0.017172842 | 0.895649127 | 1.017321143 | 0.78706287 | 1.31494236 |
| ENSG00000172243 | -0.121652848 | 0.381396507 | 0.885455703 | 0.674321211 | 1.16269782 |
| ENSG00000091181 | -1.186238224 | 0.39633813 | 0.305367832 | 0.019695102 | 4.734655074 |
| ENSG00000125826 | 0.293880664 | 0.044467323 | 1.34162379 | 1.007293533 | 1.786921425 |
| ENSG00000169508 | -0.063392968 | 0.511035602 | 0.938574571 | 0.776901279 | 1.133892103 |
| ENSG00000136854 | 0.153602291 | 0.166585031 | 1.166027055 | 0.937968163 | 1.449536505 |
| ENSG00000123728 | -0.018193487 | 0.915106036 | 0.981971015 | 0.702787563 | 1.372060528 |
| ENSG00000164692 | 0.039818009 | 0.555854558 | 1.040621373 | 0.911486353 | 1.188051624 |
| ENSG00000120738 | 0.010232117 | 0.895636592 | 1.010284644 | 0.867056069 | 1.177173078 |
| ENSG00000120949 | 0.113185064 | 0.774979988 | 1.119839155 | 0.515387922 | 2.433195814 |
| ENSG00000196735 | 0.018717522 | 0.80664453 | 1.018893793 | 0.87706996 | 1.183650802 |
| ENSG00000167850 | 0.122045785 | 0.539750488 | 1.129805829 | 0.764866576 | 1.668867815 |
| ENSG00000072274 | 0.05420185 | 0.629517569 | 1.055697673 | 0.847033972 | 1.315764909 |
| ENSG00000160654 | -0.180808844 | 0.361188829 | 0.834594882 | 0.566140444 | 1.230345974 |
| ENSG00000108691 | 0.041925455 | 0.63747121 | 1.042816739 | 0.875946462 | 1.241476275 |
| ENSG00000128383 | -0.057029984 | 0.770824643 | 0.944565747 | 0.643552568 | 1.38637385 |
| ENSG00000078596 | 0.004486049 | 0.963793841 | 1.004496126 | 0.827612765 | 1.219184274 |
| ENSG00000185507 | 0.334469773 | 0.005221 | 1.397199355 | 1.104910725 | 1.766808842 |
| ENSG00000006534 | 0.263861867 | 0.062913166 | 1.301948341 | 0.985890274 | 1.71932874 |
| ENSG00000203896 | 0.283590394 | 0.047468432 | 1.327888908 | 1.003171663 | 1.757714075 |
| ENSG00000177885 | 0.028739827 | 0.9176853 | 1.029156801 | 0.596728779 | 1.774949958 |
| ENSG00000188229 | 0.030722332 | 0.808761639 | 1.031199133 | 0.804065927 | 1.322493113 |
| ENSG00000100342 | -0.009711747 | 0.901035341 | 0.990335259 | 0.849777487 | 1.154142045 |
| ENSG00000136286 | 0.04091547 | 0.828042337 | 1.041764042 | 0.720165378 | 1.506976524 |
| ENSG00000056586 | -0.072043142 | 0.718647546 | 0.930490752 | 0.628775983 | 1.376981729 |
| ENSG00000174123 | 0.004253984 | 0.988906432 | 1.004263045 | 0.551343431 | 1.829248718 |
| ENSG00000173702 | -0.106393564 | 0.366965135 | 0.899070736 | 0.713528534 | 1.132860356 |
| ENSG00000167085 | 0.002493436 | 0.985809844 | 1.002496547 | 0.761640201 | 1.319519802 |
| ENSG00000214078 | 0.034650818 | 0.703332203 | 1.035258152 | 0.86616191 | 1.237366165 |
| ENSG00000196092 | 0.225466816 | 0.24914677 | 1.252907457 | 0.853856916 | 1.838454507 |
| ENSG00000171862 | 0.045604085 | 0.756965327 | 1.04665994 | 0.784097811 | 1.397143335 |
| ENSG00000163734 | -0.162063003 | 0.017758876 | 0.850387626 | 0.743748024 | 0.972317359 |
| ENSG00000155307 | -0.158605774 | 0.214373732 | 0.853332698 | 0.664332934 | 1.096102055 |
| ENSG00000139193 | 0.036311314 | 0.72666915 | 1.036978622 | 0.845958968 | 1.271130992 |
| ENSG00000067955 | -0.165446723 | 0.244638837 | 0.847515015 | 0.641365515 | 1.119925664 |
| ENSG00000140297 | 0.087675531 | 0.14027817 | 1.091633864 | 0.971566033 | 1.226539887 |
| ENSG00000150093 | 0.085823167 | 0.576880856 | 1.089613632 | 0.806011016 | 1.473004516 |
| ENSG00000167851 | 0.134056445 | 0.261397071 | 1.143457361 | 0.904935603 | 1.444848376 |
| ENSG00000163154 | 0.149249153 | 0.283779212 | 1.16096221 | 0.883679987 | 1.525250399 |
| ENSG00000169245 | -0.046687203 | 0.417960532 | 0.95438588 | 0.852432227 | 1.068533519 |
| ENSG00000171302 | -0.081704002 | 0.588199612 | 0.921544694 | 0.685599684 | 1.23868876 |
| ENSG00000011600 | 0.050116922 | 0.513798641 | 1.05139402 | 0.904545234 | 1.222083036 |
| ENSG00000145287 | -0.02790174 | 0.632856254 | 0.972483919 | 0.867293557 | 1.090432375 |
| ENSG00000100906 | 0.099285185 | 0.519154205 | 1.104381207 | 0.816623584 | 1.493537383 |
| ENSG00000129219 | 0.15999976 | 0.416176674 | 1.17351059 | 0.797965283 | 1.725798268 |
| ENSG00000163737 | -0.122232796 | 0.127079928 | 0.884942333 | 0.75634693 | 1.035401747 |
| ENSG00000159216 | -0.037523599 | 0.803612922 | 0.963171688 | 0.716575405 | 1.294629558 |
| ENSG00000135404 | 0.069328134 | 0.675785091 | 1.071787841 | 0.774472658 | 1.483240454 |
| ENSG00000181374 | 0.015184071 | 0.833595597 | 1.015299935 | 0.881201441 | 1.169805121 |
| ENSG00000155849 | 0.117641063 | 0.497293045 | 1.124840293 | 0.800869179 | 1.579865622 |
| ENSG00000163191 | -0.051062599 | 0.62671207 | 0.950219186 | 0.773493561 | 1.167322582 |
| ENSG00000115602 | 0.03722216 | 0.909889059 | 1.037923581 | 0.544781117 | 1.977464575 |
| ENSG00000158517 | 0.286460039 | 0.124920442 | 1.331704951 | 0.923634512 | 1.920064759 |
| ENSG00000204590 | 0.462468646 | 0.086104589 | 1.587989334 | 0.93645613 | 2.692822488 |
| ENSG00000155659 | 0.170898947 | 0.023133508 | 1.186370856 | 1.023697183 | 1.374894678 |
| ENSG00000127191 | 0.54085684 | 0.01066677 | 1.717477835 | 1.133948166 | 2.601291844 |
| ENSG00000106244 | 0.301915904 | 0.163500769 | 1.352447487 | 0.884473647 | 2.068025668 |
| ENSG00000039537 | 0.289763696 | 0.329579564 | 1.336111722 | 0.74621003 | 2.392348618 |
| ENSG00000182566 | 0.440964276 | 0.05020196 | 1.554205179 | 0.999611664 | 2.41649215 |
| ENSG00000197170 | -0.55795657 | 0.007079568 | 0.572377483 | 0.38135473 | 0.85908462 |
| ENSG00000101152 | 0.031135655 | 0.843542674 | 1.03162544 | 0.757248846 | 1.405417854 |
| ENSG00000174292 | 0.234957261 | 0.227391002 | 1.264854709 | 0.863691315 | 1.852348642 |
| ENSG00000012061 | 0.081725749 | 0.753508906 | 1.085158163 | 0.65156757 | 1.807284914 |
| ENSG00000064300 | 0.354952909 | 0.003104307 | 1.426113496 | 1.127158165 | 1.804360529 |
| ENSG00000134545 | -0.302078254 | 0.540178955 | 0.739280211 | 0.281212689 | 1.943494204 |
| ENSG00000100902 | -0.293416472 | 0.223806706 | 0.745711508 | 0.464791263 | 1.196420195 |
| ENSG00000154639 | -0.276819745 | 0.019866862 | 0.758191152 | 0.600620849 | 0.957099349 |
| ENSG00000121390 | -0.157224792 | 0.457363804 | 0.85451195 | 0.564474361 | 1.293576331 |
| ENSG00000002726 | -0.018437622 | 0.819101045 | 0.981731311 | 0.83824278 | 1.149781891 |
| ENSG00000115008 | -0.213963189 | 0.091452427 | 0.807378105 | 0.629750936 | 1.035106687 |
| ENSG00000160703 | 0.237739916 | 0.240118735 | 1.268379265 | 0.853057913 | 1.885904737 |
| ENSG00000185000 | 0.029452997 | 0.809529286 | 1.029891026 | 0.810548398 | 1.308589997 |
| ENSG00000132005 | 0.477683451 | 0.024019326 | 1.61233502 | 1.064849194 | 2.441307399 |
| ENSG00000239713 | 0.19519469 | 0.183671923 | 1.215547618 | 0.911597958 | 1.620841731 |
| ENSG00000168685 | -0.140400163 | 0.183168156 | 0.86901042 | 0.706708806 | 1.068585964 |
| ENSG00000197381 | 0.439758691 | 0.021660758 | 1.552332582 | 1.066524341 | 2.259429393 |
| ENSG00000182866 | 0.099967105 | 0.287225336 | 1.105134564 | 0.919302585 | 1.328531459 |
| ENSG00000109743 | 0.135163819 | 0.334251683 | 1.144724297 | 0.870058939 | 1.506097641 |
| ENSG00000144635 | -0.401913806 | 0.122716502 | 0.66903841 | 0.401607231 | 1.114552627 |
| ENSG00000160593 | -0.154260087 | 0.378903212 | 0.857049085 | 0.607827168 | 1.208457226 |
| ENSG00000166501 | 0.077207621 | 0.646676949 | 1.080266339 | 0.776531518 | 1.502804891 |
| ENSG00000013364 | 0.12367733 | 0.385898103 | 1.131650662 | 0.855657419 | 1.49666583 |
| ENSG00000131981 | -0.036839339 | 0.732720716 | 0.963830973 | 0.780152964 | 1.190753848 |
| ENSG00000081181 | -0.082427343 | 0.590022129 | 0.920878344 | 0.682313068 | 1.242856048 |
| ENSG00000228278 | -0.126820087 | 0.483423986 | 0.880892142 | 0.617855092 | 1.255910936 |
| ENSG00000198492 | -0.265875729 | 0.281314046 | 0.76653438 | 0.472574925 | 1.243347721 |
| ENSG00000113163 | 0.043665549 | 0.844911981 | 1.044632917 | 0.674460194 | 1.617972332 |
| ENSG00000145675 | 0.024938165 | 0.87458773 | 1.025251722 | 0.7522088 | 1.397406003 |
| ENSG00000116489 | -0.358797353 | 0.035095658 | 0.698515889 | 0.500315623 | 0.975233282 |
| ENSG00000157625 | -0.208417302 | 0.253487012 | 0.811868172 | 0.567712846 | 1.161026975 |
| ENSG00000105220 | -0.117256692 | 0.500616896 | 0.889356873 | 0.632245458 | 1.251026223 |
| ENSG00000188313 | -0.038717371 | 0.810347922 | 0.962022566 | 0.70122011 | 1.31982441 |
| ENSG00000162746 | 0.270406108 | 0.398923376 | 1.310496546 | 0.699158416 | 2.456383499 |
| ENSG00000136869 | -0.119104296 | 0.245801987 | 0.887715211 | 0.72597432 | 1.085490593 |
| ENSG00000061676 | -0.170962401 | 0.403755004 | 0.842853263 | 0.564233093 | 1.259056998 |
| ENSG00000137563 | -0.132621959 | 0.10753171 | 0.875796116 | 0.74517716 | 1.029310717 |
| ENSG00000118804 | 0.162949452 | 0.433492429 | 1.176977195 | 0.782847391 | 1.769534308 |
| ENSG00000137752 | -0.09181331 | 0.288767698 | 0.912275446 | 0.76993779 | 1.080926928 |
| ENSG00000132664 | 0.04324585 | 0.810206511 | 1.044194578 | 0.733676298 | 1.486135398 |
| ENSG00000164822 | -0.058890831 | 0.1302963 | 0.942809689 | 0.873556698 | 1.017552853 |
| ENSG00000120659 | -0.207212298 | 0.162951223 | 0.812847066 | 0.607563112 | 1.087492542 |
| ENSG00000205038 | 0.978943442 | 0.105226614 | 2.661642577 | 0.814308307 | 8.699826772 |
| ENSG00000005844 | 0.051744141 | 0.672934395 | 1.053106261 | 0.828193054 | 1.339099372 |
| ENSG00000010610 | -0.005336052 | 0.956064503 | 0.994678159 | 0.822695091 | 1.202614007 |
| ENSG00000182446 | -0.083136891 | 0.75163108 | 0.920225168 | 0.549916263 | 1.539896923 |
| ENSG00000126353 | -0.104550896 | 0.466982588 | 0.900728952 | 0.679592369 | 1.193822478 |
| ENSG00000172349 | 0.136841574 | 0.482861814 | 1.146646475 | 0.782410573 | 1.68044526 |
| ENSG00000124875 | -0.029380588 | 0.796560896 | 0.971046825 | 0.776661562 | 1.214083436 |
| ENSG00000148660 | 0.397985417 | 0.102866367 | 1.488822319 | 0.922896445 | 2.40177748 |
| ENSG00000111801 | 0.086787825 | 0.550272147 | 1.090665243 | 0.820394122 | 1.449974642 |
| ENSG00000099994 | 0.15258095 | 0.26739424 | 1.164836752 | 0.889532712 | 1.52534543 |
| ENSG00000175899 | 0.110174282 | 0.234461014 | 1.116472635 | 0.9310458 | 1.338829029 |
| ENSG00000171051 | -0.044527414 | 0.641296036 | 0.95644938 | 0.793063579 | 1.153495685 |
| ENSG00000215788 | 0.148733798 | 0.240309627 | 1.160364057 | 0.90526414 | 1.48735014 |
| ENSG00000123338 | 0.058148987 | 0.664664544 | 1.059872891 | 0.814836142 | 1.378596857 |
| ENSG00000277791 | 0.024928643 | 0.852182381 | 1.02524196 | 0.788767428 | 1.332612174 |
| ENSG00000277632 | -0.075864487 | 0.436152414 | 0.926941811 | 0.7658175 | 1.1219659 |
| ENSG00000167996 | -0.003101218 | 0.979668155 | 0.996903586 | 0.785365748 | 1.265419026 |
| ENSG00000105372 | 0.090404586 | 0.465552334 | 1.094617061 | 0.858636767 | 1.395452135 |
| ENSG00000122224 | 0.137014498 | 0.726378765 | 1.146844775 | 0.532394967 | 2.470445853 |
| ENSG00000184678 | 0.254515445 | 0.006398841 | 1.289836473 | 1.074185451 | 1.548781102 |
| ENSG00000111321 | 0.323162409 | 0.162365515 | 1.381489699 | 0.877940604 | 2.173852969 |
| ENSG00000107371 | -0.143846639 | 0.50171811 | 0.866020552 | 0.569199964 | 1.31762411 |
| ENSG00000122986 | 0.277471174 | 0.183649892 | 1.319788074 | 0.876736295 | 1.986732579 |
| ENSG00000108219 | 0.79522085 | 0.009068866 | 2.214930111 | 1.21887458 | 4.024955051 |
| ENSG00000108771 | 0.341247894 | 0.041643509 | 1.40670191 | 1.012999718 | 1.953416402 |
| ENSG00000136630 | 0.504823688 | 0.006232215 | 1.6566934 | 1.153840554 | 2.378693498 |
| ENSG00000132530 | 0.174263497 | 0.181441451 | 1.190369183 | 0.921894293 | 1.537029573 |
| ENSG00000142512 | 0.057448791 | 0.661908237 | 1.059131032 | 0.818692685 | 1.370182686 |
| ENSG00000115828 | -0.091469814 | 0.213446382 | 0.912588863 | 0.790122839 | 1.054036654 |
| ENSG00000080824 | -0.053783368 | 0.717786282 | 0.947637373 | 0.707902479 | 1.268559748 |
| ENSG00000135316 | -0.168292014 | 0.280557675 | 0.845107016 | 0.622526429 | 1.147269954 |
| ENSG00000130726 | 0.053150608 | 0.704597932 | 1.054588462 | 0.801215671 | 1.388086709 |
| ENSG00000197122 | 0.048561976 | 0.777270338 | 1.049760429 | 0.74983121 | 1.469660031 |
| ENSG00000101558 | -0.162041623 | 0.380928194 | 0.850405807 | 0.591841556 | 1.221931833 |
| ENSG00000129465 | -0.204444213 | 0.285604152 | 0.815100213 | 0.560066526 | 1.186266856 |
| ENSG00000136754 | -0.10461258 | 0.675952782 | 0.900673394 | 0.551485051 | 1.470960202 |
| ENSG00000164062 | -0.075855504 | 0.61004257 | 0.926950137 | 0.692556119 | 1.240674271 |
| ENSG00000180210 | -0.293082845 | 0.323345035 | 0.745960339 | 0.416977596 | 1.334500541 |
| ENSG00000143753 | 0.108529721 | 0.517452717 | 1.114638036 | 0.802438673 | 1.548302685 |
| ENSG00000239306 | -0.178362967 | 0.519257436 | 0.836638696 | 0.486370938 | 1.439157344 |
| ENSG00000138623 | 0.095434589 | 0.446011264 | 1.100136859 | 0.860699893 | 1.406182477 |
| ENSG00000152292 | 0.524726853 | 0.010929302 | 1.689997168 | 1.128190118 | 2.531568378 |
| ENSG00000163754 | 0.090960757 | 0.641065166 | 1.095226024 | 0.747185664 | 1.60538418 |
| ENSG00000185338 | -0.057938789 | 0.552811322 | 0.943707711 | 0.779378994 | 1.142684433 |
| ENSG00000160323 | 0.61933803 | 0.00214047 | 1.857697894 | 1.250995222 | 2.758636807 |
| ENSG00000163735 | -0.023270544 | 0.663030551 | 0.976998127 | 0.879903134 | 1.084807297 |
| ENSG00000159110 | 0.039101527 | 0.873186487 | 1.039876054 | 0.643362577 | 1.680766407 |
| ENSG00000136628 | -0.133149574 | 0.487565915 | 0.875334155 | 0.601045745 | 1.27479462 |
| ENSG00000244687 | -0.09637065 | 0.576508932 | 0.908127357 | 0.647541193 | 1.273579664 |
| ENSG00000263528 | -0.104671119 | 0.564937099 | 0.900620671 | 0.630569544 | 1.286325357 |
| ENSG00000165283 | -0.042308 | 0.774317741 | 0.958574494 | 0.717842064 | 1.280037917 |
| ENSG00000197903 | 0.121170672 | 0.211978751 | 1.128817553 | 0.933229482 | 1.365397357 |
| ENSG00000109684 | -1.303790099 | 0.244413767 | 0.271500826 | 0.030224952 | 2.438802819 |
| ENSG00000162739 | 0.045845294 | 0.7421809 | 1.046912435 | 0.796683112 | 1.375736011 |
| ENSG00000117560 | -0.007456997 | 0.973938581 | 0.992570737 | 0.634551229 | 1.552588071 |
| ENSG00000164078 | 0.067555614 | 0.622801672 | 1.069889758 | 0.817399642 | 1.40037264 |
| ENSG00000158869 | 0.017135848 | 0.832961224 | 1.017283509 | 0.867526387 | 1.192892519 |
| ENSG00000181467 | 0.062939923 | 0.756801387 | 1.064962857 | 0.715050756 | 1.58610543 |
| ENSG00000085063 | 0.10639115 | 0.552845018 | 1.112256851 | 0.782742319 | 1.580488586 |
| ENSG00000186827 | 0.056377592 | 0.691512951 | 1.057997099 | 0.800840552 | 1.397728748 |
| ENSG00000172216 | 0.029526033 | 0.80563445 | 1.029966249 | 0.814110884 | 1.303054037 |
| ENSG00000196189 | -0.03595289 | 0.846947186 | 0.964685739 | 0.669626524 | 1.389757637 |
| ENSG00000109339 | 0.473055036 | 0.182333048 | 1.604889708 | 0.80077044 | 3.216491075 |
| ENSG00000134954 | 0.05118345 | 0.652172064 | 1.05251596 | 0.842505174 | 1.314876014 |
| ENSG00000119919 | 0.096658931 | 0.362398019 | 1.101484627 | 0.894633034 | 1.356163185 |
| ENSG00000105639 | 0.101909366 | 0.531003014 | 1.10728311 | 0.804994549 | 1.523085948 |
| ENSG00000101017 | 0.080583732 | 0.435420289 | 1.083919602 | 0.885220975 | 1.327218554 |
| ENSG00000107758 | -0.050000225 | 0.818871088 | 0.951229211 | 0.620054993 | 1.459285098 |
| ENSG00000169894 | -0.050966577 | 0.644878848 | 0.950310432 | 0.765132661 | 1.180305016 |
| ENSG00000184451 | 0.21312561 | 0.411812059 | 1.237540088 | 0.743901117 | 2.058748717 |
| ENSG00000241106 | 0.16381852 | 0.316144061 | 1.178000512 | 0.855142709 | 1.622752777 |
| ENSG00000102879 | -0.012745093 | 0.906069309 | 0.987335782 | 0.798961041 | 1.220124507 |
| ENSG00000049860 | -0.492180332 | 0.020749325 | 0.61129212 | 0.402795591 | 0.927711387 |
| ENSG00000163220 | -0.017613171 | 0.779137112 | 0.982541034 | 0.868742912 | 1.111245767 |
| ENSG00000078140 | -0.457227046 | 0.045314214 | 0.633036595 | 0.404576283 | 0.990506235 |
| ENSG00000102393 | -0.286632521 | 0.059368417 | 0.750787577 | 0.557333152 | 1.011391452 |
| ENSG00000105974 | 0.12672318 | 0.171936422 | 1.135102755 | 0.946389858 | 1.361445553 |
| ENSG00000099341 | -0.128349276 | 0.415598266 | 0.879546121 | 0.645738887 | 1.19800959 |
| ENSG00000012779 | 0.10078199 | 0.273987023 | 1.106035489 | 0.923313169 | 1.324918287 |
| ENSG00000100292 | 0.012518404 | 0.902812752 | 1.012597087 | 0.828271868 | 1.237942396 |
| ENSG00000198788 | -0.048673446 | 0.192284705 | 0.952492119 | 0.885290015 | 1.024795515 |
| ENSG00000129083 | -0.061471493 | 0.787358073 | 0.940379753 | 0.601621872 | 1.469883528 |
| ENSG00000177830 | 0.262794633 | 0.173165607 | 1.3005596 | 0.89105661 | 1.898257926 |
| ENSG00000166710 | 0.021990765 | 0.851199275 | 1.022234344 | 0.812391096 | 1.286280781 |
| ENSG00000124839 | 0.364352363 | 0.034927895 | 1.43958138 | 1.026113752 | 2.01965381 |
| ENSG00000113916 | 0.269137227 | 0.052479077 | 1.308834736 | 0.997119963 | 1.717996258 |
| ENSG00000100567 | -0.215468669 | 0.226226066 | 0.806163528 | 0.568675006 | 1.142831366 |
| ENSG00000182326 | 0.082323105 | 0.311235978 | 1.085806582 | 0.92587646 | 1.273362036 |
| ENSG00000149273 | 0.007880489 | 0.950124172 | 1.007911622 | 0.787378484 | 1.29021285 |
| ENSG00000204577 | 0.078688752 | 0.731974609 | 1.08186754 | 0.689623593 | 1.69721191 |
| ENSG00000096996 | 0.107081034 | 0.505523039 | 1.113024443 | 0.81210192 | 1.525453125 |
| ENSG00000022556 | 0.085391811 | 0.345921718 | 1.089143722 | 0.911942688 | 1.300776971 |
| ENSG00000100298 | 0.341941093 | 0.194521164 | 1.407677373 | 0.839745472 | 2.359709758 |
| ENSG00000033627 | -0.060749157 | 0.803941445 | 0.941059268 | 0.582531331 | 1.520248782 |
| ENSG00000100226 | 0.343891549 | 0.152191764 | 1.410425665 | 0.880870713 | 2.258334315 |
| ENSG00000155918 | -0.095337565 | 0.495264712 | 0.909066014 | 0.691190675 | 1.195619455 |
| ENSG00000167333 | 0.281389808 | 0.25144184 | 1.324969987 | 0.819137834 | 2.143162474 |
| ENSG00000137628 | -0.043600629 | 0.689627164 | 0.957336213 | 0.772918571 | 1.185755731 |
| ENSG00000156482 | 0.095072438 | 0.521820936 | 1.099738515 | 0.822147352 | 1.471056007 |
| ENSG00000169918 | 0.299152747 | 0.613511783 | 1.34871562 | 0.42242642 | 4.306155432 |
| ENSG00000181555 | -0.163536155 | 0.414006605 | 0.849135798 | 0.573543622 | 1.25715216 |
| ENSG00000035403 | 0.173161457 | 0.24294929 | 1.189058071 | 0.88914027 | 1.590141786 |
| ENSG00000185436 | -0.189743914 | 0.249496384 | 0.827170934 | 0.59888497 | 1.142476081 |
| ENSG00000158092 | 0.132357853 | 0.530166815 | 1.141516741 | 0.755111742 | 1.725652507 |
| ENSG00000106785 | 0.146140118 | 0.466338204 | 1.157358344 | 0.78109086 | 1.714881589 |
| ENSG00000109079 | -0.200293339 | 0.466638523 | 0.818490623 | 0.477321552 | 1.403512783 |
| ENSG00000090487 | -0.049528386 | 0.848696236 | 0.951678143 | 0.572153095 | 1.582952703 |
| ENSG00000119699 | 0.181346263 | 0.082760778 | 1.198830218 | 0.976747528 | 1.471407761 |
| ENSG00000090339 | 0.026670363 | 0.797834016 | 1.027029201 | 0.83744453 | 1.259532949 |
| ENSG00000240972 | 0.017144533 | 0.839199714 | 1.017292345 | 0.862040214 | 1.200505148 |
| ENSG00000110057 | 0.137451534 | 0.308842544 | 1.147346097 | 0.880491408 | 1.495077697 |
| ENSG00000172936 | -0.156325046 | 0.50775601 | 0.85528114 | 0.538527163 | 1.358345277 |
| ENSG00000100321 | 0.354700667 | 0.051324608 | 1.425753816 | 0.997962013 | 2.03692517 |
| ENSG00000114021 | -0.242900805 | 0.236346229 | 0.784349313 | 0.524696443 | 1.172494789 |
| ENSG00000149294 | 0.276318273 | 0.126403881 | 1.318267366 | 0.924952063 | 1.878831258 |
| ENSG00000101856 | -0.086710365 | 0.644697884 | 0.916942636 | 0.634288438 | 1.325554347 |
| ENSG00000163568 | 0.093725286 | 0.364522235 | 1.098257998 | 0.896858392 | 1.344884144 |
| ENSG00000100764 | -0.175851303 | 0.52774021 | 0.838742693 | 0.485940142 | 1.447687161 |
| ENSG00000076944 | 0.242819 | 0.230599207 | 1.274837858 | 0.857126704 | 1.89611589 |
| ENSG00000115091 | -0.333410066 | 0.163397591 | 0.716476331 | 0.44830849 | 1.145056015 |
| ENSG00000095002 | -0.060406268 | 0.69066695 | 0.941382002 | 0.699131666 | 1.267572499 |
| ENSG00000204305 | 0.20436093 | 0.292518208 | 1.226740841 | 0.838484908 | 1.794776599 |
| ENSG00000132466 | -0.183065481 | 0.339489517 | 0.832713627 | 0.571948959 | 1.212366899 |
| ENSG00000175505 | 0.119653817 | 0.458619787 | 1.127106599 | 0.821369903 | 1.546646986 |
| ENSG00000134996 | -0.041369232 | 0.859935518 | 0.959474796 | 0.606001757 | 1.519124117 |
| ENSG00000258659 | -0.05955708 | 0.938656903 | 0.942181753 | 0.206727535 | 4.294089101 |
| ENSG00000180228 | -0.089367969 | 0.708281223 | 0.914509001 | 0.57264015 | 1.460475156 |
| ENSG00000113303 | -0.046387991 | 0.547809531 | 0.954671486 | 0.82065238 | 1.110576986 |
| ENSG00000184500 | -0.03880816 | 0.709679266 | 0.961935229 | 0.78417745 | 1.179987239 |
| ENSG00000143185 | 0.043331834 | 0.838280983 | 1.044284366 | 0.68880526 | 1.583219382 |
| ENSG00000166167 | 0.161843511 | 0.600111705 | 1.175676247 | 0.641954902 | 2.15313355 |
| ENSG00000081189 | 0.113954899 | 0.481140941 | 1.120701579 | 0.816205599 | 1.538793695 |
| ENSG00000162889 | 0.196122544 | 0.414765016 | 1.216675993 | 0.759408894 | 1.949279871 |
| ENSG00000166598 | -0.0635264 | 0.66614329 | 0.938449344 | 0.703201312 | 1.252396939 |
| ENSG00000187908 | -0.020250705 | 0.614779878 | 0.979952963 | 0.905636344 | 1.060368012 |
| ENSG00000145703 | -0.032693811 | 0.74324084 | 0.967834854 | 0.795873271 | 1.176951583 |
| ENSG00000223865 | 0.03038936 | 0.679247213 | 1.03085583 | 0.892563542 | 1.190574893 |
| ENSG00000143119 | -0.020189059 | 0.800606373 | 0.980013376 | 0.837894973 | 1.146236996 |
| ENSG00000204525 | 0.011470971 | 0.91480693 | 1.011537014 | 0.819802304 | 1.248114486 |
| ENSG00000102837 | -0.019257084 | 0.509268937 | 0.980927149 | 0.926403145 | 1.038660196 |
| ENSG00000100060 | 0.357039159 | 0.005236461 | 1.42909183 | 1.112278513 | 1.836143947 |
| ENSG00000135535 | -0.156374599 | 0.332221992 | 0.855238759 | 0.623468572 | 1.173167931 |
| ENSG00000135870 | -0.227567204 | 0.229853405 | 0.796468894 | 0.54934673 | 1.154758305 |
| ENSG00000079385 | -0.05824118 | 0.494437646 | 0.943422385 | 0.798271202 | 1.114966686 |
| ENSG00000066294 | -0.101125944 | 0.497494303 | 0.903819195 | 0.674844816 | 1.210484423 |
| ENSG00000033122 | 0.020502521 | 0.985360827 | 1.020714142 | 0.114229019 | 9.120776592 |
| ENSG00000198805 | -0.272490495 | 0.106531474 | 0.761480667 | 0.546952304 | 1.060152414 |
| ENSG00000123143 | 0.302296779 | 0.018217759 | 1.352962699 | 1.052706989 | 1.738858089 |
| ENSG00000141524 | 0.195045066 | 0.20074106 | 1.215365758 | 0.901457666 | 1.638583795 |
| ENSG00000180370 | 0.054712323 | 0.791294124 | 1.056236716 | 0.704328798 | 1.583970444 |
| ENSG00000157601 | 0.112345663 | 0.156637498 | 1.118899556 | 0.957809451 | 1.307082755 |
| ENSG00000145920 | 0.166488142 | 0.157686081 | 1.18114953 | 0.937570378 | 1.488010121 |
| ENSG00000146243 | -0.142321837 | 0.562966725 | 0.867342069 | 0.535497395 | 1.404828988 |
| ENSG00000069702 | 0.190591815 | 0.121652747 | 1.209965461 | 0.950524569 | 1.540219437 |
| ENSG00000175857 | -0.27776823 | 0.376107985 | 0.757472361 | 0.409481091 | 1.401198712 |
| ENSG00000131503 | -0.186161122 | 0.563073132 | 0.83013983 | 0.441704706 | 1.560164808 |
| ENSG00000171388 | 0.001325754 | 0.990898963 | 1.001326634 | 0.797341028 | 1.25749835 |
| ENSG00000196230 | -0.236574383 | 0.249443181 | 0.789327168 | 0.527727174 | 1.180605071 |
| ENSG00000146833 | 0.084185238 | 0.700446908 | 1.087830383 | 0.708431963 | 1.670414385 |
| ENSG00000148248 | 0.166332876 | 0.467328757 | 1.180966151 | 0.754124598 | 1.849404003 |
| ENSG00000088832 | 0.262899555 | 0.132259115 | 1.300696064 | 0.923654231 | 1.831648894 |
| ENSG00000186891 | 0.041612993 | 0.747931299 | 1.042490949 | 0.808821757 | 1.34366734 |
| ENSG00000127481 | 0.057109241 | 0.768534931 | 1.058771466 | 0.723804082 | 1.548757522 |
| ENSG00000271503 | 0.01817264 | 0.802980422 | 1.018338767 | 0.882860148 | 1.174607154 |
| ENSG00000163736 | -0.021364816 | 0.670362796 | 0.978861795 | 0.887149278 | 1.080055451 |
| ENSG00000171298 | 0.223912268 | 0.03567216 | 1.250961265 | 1.015108467 | 1.541612682 |
| ENSG00000077420 | 0.078986233 | 0.568274421 | 1.082189423 | 0.825036629 | 1.419493276 |
| ENSG00000143862 | 0.490779916 | 0.024368429 | 1.633589786 | 1.065564967 | 2.504413782 |
| ENSG00000117592 | 0.11717722 | 0.489956412 | 1.124318664 | 0.806149893 | 1.56806131 |
| ENSG00000169375 | 0.334654458 | 0.229835066 | 1.397457421 | 0.809303739 | 2.413046115 |
| ENSG00000185127 | -0.064385233 | 0.759700204 | 0.937643719 | 0.620674177 | 1.416485132 |
| ENSG00000114251 | -0.289747251 | 0.019623404 | 0.748452714 | 0.586773706 | 0.954680586 |
| ENSG00000119396 | 0.046376492 | 0.844358339 | 1.047468701 | 0.659271485 | 1.664247132 |
| ENSG00000115598 | 0.321860066 | 0.028861894 | 1.379691697 | 1.033754554 | 1.841393753 |
| ENSG00000107281 | 0.266905785 | 0.011345019 | 1.305917404 | 1.062145499 | 1.605637145 |
| ENSG00000172175 | -0.349018669 | 0.085474773 | 0.705379961 | 0.473888055 | 1.049954486 |
| ENSG00000181634 | -0.116182053 | 0.478360524 | 0.890313124 | 0.64572423 | 1.227547957 |
| ENSG00000051382 | -0.134346647 | 0.495048899 | 0.874286943 | 0.594361044 | 1.286049391 |
| ENSG00000159189 | 0.108557602 | 0.128415819 | 1.114669114 | 0.969100334 | 1.282103814 |
| ENSG00000116514 | -0.060971204 | 0.703898294 | 0.940850333 | 0.687017201 | 1.288467519 |
| ENSG00000029993 | -0.032378384 | 0.831280358 | 0.968140184 | 0.718757469 | 1.304049636 |
| ENSG00000107779 | -0.02549869 | 0.91225231 | 0.974823656 | 0.61939375 | 1.534211738 |
| ENSG00000168329 | 0.050656094 | 0.877043204 | 1.051961055 | 0.553744535 | 1.998434281 |
| ENSG00000160791 | -0.070473541 | 0.62052335 | 0.931952398 | 0.70507305 | 1.231837284 |
| ENSG00000117322 | 0.105215871 | 0.276994593 | 1.110950406 | 0.918988527 | 1.343010025 |
| ENSG00000174600 | 0.105707627 | 0.431034653 | 1.111496857 | 0.8543554 | 1.446032018 |
| ENSG00000120306 | -0.106965897 | 0.449650961 | 0.898556316 | 0.680940142 | 1.185718689 |
| ENSG00000163961 | 0.076798068 | 0.707384646 | 1.079824003 | 0.723108607 | 1.612510026 |
| ENSG00000103490 | 0.130912892 | 0.219097157 | 1.139868485 | 0.925081296 | 1.404525384 |
| ENSG00000127507 | -0.053857843 | 0.785551815 | 0.9475668 | 0.642869351 | 1.396680118 |
| ENSG00000189430 | -0.058524812 | 0.93088158 | 0.943154839 | 0.25132513 | 3.539403519 |
| ENSG00000139219 | -0.083686996 | 0.70369294 | 0.919719087 | 0.597535396 | 1.415620237 |
| ENSG00000164400 | -0.076225235 | 0.622439585 | 0.926607479 | 0.684109635 | 1.255064065 |
| ENSG00000102882 | 0.138025063 | 0.310682234 | 1.148004322 | 0.879133161 | 1.499106145 |
| ENSG00000162924 | -0.246144492 | 0.284619661 | 0.781809252 | 0.498068314 | 1.227192514 |
| ENSG00000107263 | 0.393275875 | 0.076501665 | 1.481827132 | 0.958989746 | 2.289713376 |
| ENSG00000149476 | 0.221001498 | 0.186630853 | 1.247325299 | 0.898530882 | 1.731515782 |
| ENSG00000150760 | 0.128673975 | 0.477833182 | 1.137319269 | 0.797210681 | 1.622526078 |
| ENSG00000116701 | 0.009669702 | 0.917020316 | 1.009716605 | 0.841781189 | 1.211155151 |
| ENSG00000105426 | 0.088489657 | 0.468149716 | 1.092522952 | 0.860216963 | 1.387564362 |
| ENSG00000058404 | 1.308956487 | 0.000498946 | 3.702308288 | 1.771845316 | 7.736051525 |
| ENSG00000102580 | -0.297157981 | 0.057824347 | 0.742926635 | 0.546525325 | 1.009907427 |
| ENSG00000148400 | 0.156586438 | 0.251013949 | 1.169511848 | 0.895138386 | 1.527984927 |
| ENSG00000134215 | -0.126904178 | 0.075745244 | 0.88081807 | 0.765704151 | 1.013237909 |
| ENSG00000134644 | -0.362018087 | 0.166401271 | 0.696269774 | 0.416968317 | 1.162658118 |
| ENSG00000182885 | -0.298042832 | 0.156120275 | 0.742269546 | 0.491679394 | 1.120575899 |
| ENSG00000072803 | -0.286578053 | 0.24853992 | 0.750828472 | 0.461466557 | 1.221634342 |
| ENSG00000196743 | -0.080168954 | 0.670697654 | 0.922960395 | 0.637808827 | 1.335597526 |
| ENSG00000166086 | 0.278318876 | 0.038688034 | 1.320907335 | 1.014580036 | 1.719722571 |
| ENSG00000115232 | -0.110571908 | 0.485300085 | 0.895321947 | 0.656296846 | 1.221400642 |
| ENSG00000108821 | 0.036188318 | 0.548245345 | 1.036851086 | 0.921320238 | 1.166869163 |
| ENSG00000172016 | -0.045623203 | 0.19499732 | 0.955401887 | 0.891701928 | 1.02365234 |
| ENSG00000085265 | 0.120162782 | 0.409397199 | 1.127680403 | 0.847617996 | 1.500278542 |
| ENSG00000254415 | -0.115894151 | 0.484014902 | 0.890569485 | 0.643742266 | 1.232036561 |
| ENSG00000025434 | 0.153862797 | 0.370394251 | 1.166330852 | 0.832930214 | 1.633183228 |
| ENSG00000115590 | -0.048057493 | 0.562889982 | 0.95307899 | 0.80988622 | 1.121589105 |
| ENSG00000105376 | 0.456134278 | 0.145168344 | 1.577962216 | 0.854243322 | 2.914819105 |
| ENSG00000186818 | 0.028708798 | 0.802253087 | 1.029124868 | 0.822032326 | 1.288389715 |
| ENSG00000125910 | -0.049949184 | 0.716567351 | 0.951277763 | 0.72643594 | 1.245711196 |
| ENSG00000134028 | 0.00171619 | 0.977170137 | 1.001717663 | 0.89063089 | 1.126660087 |
| ENSG00000129654 | -0.002474994 | 0.974832001 | 0.997528066 | 0.855358701 | 1.163327433 |
| ENSG00000168461 | 0.007632722 | 0.927986498 | 1.007661926 | 0.853942035 | 1.189053256 |
| ENSG00000160678 | 0.270885117 | 0.148154094 | 1.311124436 | 0.908225971 | 1.892752839 |
| ENSG00000167182 | 0.151420003 | 0.522417718 | 1.163485223 | 0.731565482 | 1.850412435 |
| ENSG00000072958 | 0.350329995 | 0.164505885 | 1.419535911 | 0.866212936 | 2.326312757 |
| ENSG00000057663 | -0.158634126 | 0.456496421 | 0.853308505 | 0.562040759 | 1.295520643 |
| ENSG00000000003 | -0.109977985 | 0.274281391 | 0.895853857 | 0.735544708 | 1.09110177 |
| ENSG00000244734 | 0.072692797 | 0.17672986 | 1.07540012 | 0.967756564 | 1.195016869 |
| ENSG00000092929 | -0.001316951 | 0.989550172 | 0.998683916 | 0.820046693 | 1.216235091 |
| ENSG00000137154 | 0.009625952 | 0.942890615 | 1.00967243 | 0.775896614 | 1.313884348 |
| ENSG00000204613 | 0.111186472 | 0.655980157 | 1.117603289 | 0.685224967 | 1.822813195 |
| ENSG00000118046 | 0.156541202 | 0.384606881 | 1.169458946 | 0.821726821 | 1.664341715 |
| ENSG00000120217 | -0.038618417 | 0.804308274 | 0.962117767 | 0.708858275 | 1.305861314 |
| ENSG00000105501 | -0.10096421 | 0.833545075 | 0.903965386 | 0.352545642 | 2.31786561 |
| ENSG00000176945 | 0.086895928 | 0.359930742 | 1.090783154 | 0.905618343 | 1.313807189 |
| ENSG00000006606 | -0.059920528 | 0.65470824 | 0.94183938 | 0.724322354 | 1.224677677 |
| ENSG00000198791 | -0.495351049 | 0.002751592 | 0.609356955 | 0.440607605 | 0.842736019 |
| ENSG00000179934 | -0.484045551 | 0.113657186 | 0.616285129 | 0.338324552 | 1.122612466 |
| ENSG00000143106 | -0.423645831 | 0.022417217 | 0.654655699 | 0.455066472 | 0.941783478 |
| ENSG00000167658 | 0.057878195 | 0.680889015 | 1.059585925 | 0.804156356 | 1.396149299 |
| ENSG00000050748 | -0.717928481 | 0.025294796 | 0.487761617 | 0.260023525 | 0.914961043 |
| ENSG00000019169 | 0.065438674 | 0.345705167 | 1.067627262 | 0.931854881 | 1.223181842 |
| ENSG00000109107 | 0.189001617 | 0.068003581 | 1.208042906 | 0.986118161 | 1.479911555 |
| ENSG00000179750 | -0.021397207 | 0.836948597 | 0.978830089 | 0.798373907 | 1.200074719 |
| ENSG00000138795 | 0.083582542 | 0.476380066 | 1.087174949 | 0.863763907 | 1.368370871 |
| ENSG00000103653 | 0.252086928 | 0.172856126 | 1.286707884 | 0.895488179 | 1.848843143 |
| ENSG00000183020 | 0.366430755 | 0.162923645 | 1.442576506 | 0.86218214 | 2.413674419 |
| ENSG00000168394 | -0.026113562 | 0.797672882 | 0.974224448 | 0.797908696 | 1.189501105 |
| ENSG00000153162 | 0.127386307 | 0.414784123 | 1.135855722 | 0.836297187 | 1.542715007 |
| ENSG00000174837 | -0.063742873 | 0.834407881 | 0.938246217 | 0.516146768 | 1.705534196 |
| ENSG00000100600 | 0.058940274 | 0.69053699 | 1.060711887 | 0.793564186 | 1.417792948 |
| ENSG00000198502 | -0.015145024 | 0.79491903 | 0.984969085 | 0.878671151 | 1.104126496 |
| ENSG00000147251 | 0.056712105 | 0.664867584 | 1.058351072 | 0.818834084 | 1.367929124 |
| ENSG00000131236 | -0.258475065 | 0.230903925 | 0.772228286 | 0.505940521 | 1.178669235 |
| ENSG00000203710 | 0.059593763 | 0.837573243 | 1.061405277 | 0.600390946 | 1.876412644 |
| ENSG00000085719 | -0.249292533 | 0.124245933 | 0.779351954 | 0.567140977 | 1.070967348 |
| ENSG00000176083 | 0.150883668 | 0.297820966 | 1.162861372 | 0.875321927 | 1.544856275 |
| ENSG00000184956 | 0.048406744 | 0.483176265 | 1.049597486 | 0.91677143 | 1.201667992 |
| ENSG00000134460 | -0.034459276 | 0.81253811 | 0.966127683 | 0.726693944 | 1.284450915 |
| ENSG00000153879 | -0.221193629 | 0.298029067 | 0.801561459 | 0.528462836 | 1.215791783 |
| ENSG00000142185 | 0.105029671 | 0.318302072 | 1.110743567 | 0.903709864 | 1.365207264 |
| ENSG00000113194 | -0.404054722 | 0.1479025 | 0.667607587 | 0.386217318 | 1.154013219 |
| ENSG00000107485 | 0.077943921 | 0.75699351 | 1.081062032 | 0.659838909 | 1.771182481 |
| ENSG00000131187 | 0.077661823 | 0.466696872 | 1.080757109 | 0.87681177 | 1.332139884 |
| ENSG00000173457 | 0.076976884 | 0.629234164 | 1.080017111 | 0.790165021 | 1.476194123 |
| ENSG00000276409 | 0.249677643 | 0.439933591 | 1.283611569 | 0.681160931 | 2.418897775 |
| ENSG00000171475 | 0.109628328 | 0.507042432 | 1.115863258 | 0.807158109 | 1.542635572 |
| ENSG00000179869 | 0.413223668 | 0.463361597 | 1.511683104 | 0.500971468 | 4.561508891 |
| ENSG00000143387 | -0.039770821 | 0.631477831 | 0.961009657 | 0.816862424 | 1.130593763 |
| ENSG00000161980 | -0.150056936 | 0.332631291 | 0.860658972 | 0.6353202 | 1.165922108 |
| ENSG00000239998 | -0.249578382 | 0.566888125 | 0.779129209 | 0.331606989 | 1.830607751 |
| ENSG00000101916 | -0.192169903 | 0.313245036 | 0.825166658 | 0.567982278 | 1.198805033 |
| ENSG00000132514 | -0.048584871 | 0.672302079 | 0.952576489 | 0.760554811 | 1.193078993 |
| ENSG00000241553 | -0.186678453 | 0.394368311 | 0.829710485 | 0.539960804 | 1.274943445 |
| ENSG00000188404 | 0.010478322 | 0.919104765 | 1.010533412 | 0.825525183 | 1.237003786 |
| ENSG00000160712 | 0.05690695 | 0.612148257 | 1.058557307 | 0.849523003 | 1.319026758 |
| ENSG00000204463 | 0.409083228 | 0.080267951 | 1.50543701 | 0.951887383 | 2.380891513 |
| ENSG00000187796 | 0.194508659 | 0.291593305 | 1.214714001 | 0.846222813 | 1.743666187 |
| ENSG00000196083 | -0.103304846 | 0.667828328 | 0.901852006 | 0.562632693 | 1.445591503 |
| ENSG00000065883 | 0.16183091 | 0.49068608 | 1.175661432 | 0.742022437 | 1.862719689 |
| ENSG00000204977 | -0.376893021 | 0.060717946 | 0.685989456 | 0.462665761 | 1.017109054 |
| ENSG00000173530 | -0.021467658 | 0.885532189 | 0.978761132 | 0.730707164 | 1.311022254 |
| ENSG00000107562 | 0.132121175 | 0.114646169 | 1.141246602 | 0.968489967 | 1.344819101 |
| ENSG00000131323 | -0.203412215 | 0.411931633 | 0.815941829 | 0.501921107 | 1.326425726 |
| ENSG00000105205 | -0.114181295 | 0.154789586 | 0.892096208 | 0.762259015 | 1.044048846 |
| ENSG00000223496 | -0.005475571 | 0.982437033 | 0.994539392 | 0.610799325 | 1.619367543 |
| ENSG00000172116 | -0.036023234 | 0.797905564 | 0.964617881 | 0.732158762 | 1.270882362 |
| ENSG00000171206 | 0.557238465 | 0.009965552 | 1.745844626 | 1.142739741 | 2.667250774 |
| ENSG00000092445 | 0.067816246 | 0.688450244 | 1.070168643 | 0.768217477 | 1.490803006 |
| ENSG00000134352 | 0.008002896 | 0.949352269 | 1.008035005 | 0.787468032 | 1.290381996 |
| ENSG00000005020 | -0.041464982 | 0.760201299 | 0.959382931 | 0.735110387 | 1.252078088 |
| ENSG00000106588 | -0.053072504 | 0.745632969 | 0.948311254 | 0.688167038 | 1.306796438 |
| ENSG00000164764 | -0.024878859 | 0.753907671 | 0.975428069 | 0.834917015 | 1.139586212 |
| ENSG00000112195 | 0.386005753 | 0.022450406 | 1.471093135 | 1.05608536 | 2.049185694 |
| ENSG00000106804 | 0.293803549 | 0.184586489 | 1.341520335 | 0.869168835 | 2.07057218 |
| ENSG00000124181 | 0.06892365 | 0.600903211 | 1.071354408 | 0.827521533 | 1.387033716 |
| ENSG00000162736 | 0.175506543 | 0.506973484 | 1.191849787 | 0.709715716 | 2.001513963 |
| ENSG00000164136 | -0.226719193 | 0.398258951 | 0.797144594 | 0.471066569 | 1.348937806 |
| ENSG00000160691 | 0.230771132 | 0.347765811 | 1.259570931 | 0.778061386 | 2.039066531 |
| ENSG00000136848 | 0.195162167 | 0.208011384 | 1.215508086 | 0.897047632 | 1.647025036 |
| ENSG00000065618 | 0.031367384 | 0.652997124 | 1.031864524 | 0.899988502 | 1.183064444 |
| ENSG00000060971 | -0.072526209 | 0.690507088 | 0.930041371 | 0.650812244 | 1.329072954 |
| ENSG00000275302 | -0.125640813 | 0.226851613 | 0.881931568 | 0.719351231 | 1.081256636 |
| ENSG00000255587 | -1.945190681 | 0.11966966 | 0.142959961 | 0.01233726 | 1.656571285 |
| ENSG00000158813 | -0.15157741 | 0.289682427 | 0.859351357 | 0.64910538 | 1.137696249 |
| ENSG00000100453 | -0.054256567 | 0.446836687 | 0.947189058 | 0.823616562 | 1.089301932 |
| ENSG00000121410 | 0.744117865 | 0.776855895 | 2.104584088 | 0.012255483 | 361.4116476 |
| ENSG00000100351 | -0.058956723 | 0.840647448 | 0.942747568 | 0.530645766 | 1.674889414 |
| ENSG00000138646 | 0.289417614 | 0.121825007 | 1.335649398 | 0.925685244 | 1.927177002 |
| ENSG00000179348 | 0.370874147 | 0.022569487 | 1.449000701 | 1.053532158 | 1.992917839 |
| ENSG00000196730 | 0.320345258 | 0.001818701 | 1.37760331 | 1.126369451 | 1.684874247 |
| ENSG00000069974 | -0.058721864 | 0.671536692 | 0.942969006 | 0.718822056 | 1.237010661 |
| ENSG00000142583 | 0.225557054 | 0.102801997 | 1.253020522 | 0.955592145 | 1.643023581 |
| ENSG00000148346 | -0.043260692 | 0.327245158 | 0.957661703 | 0.87826322 | 1.044238124 |
| ENSG00000102524 | 0.069658174 | 0.569321931 | 1.072141633 | 0.843442562 | 1.362852353 |
| ENSG00000168884 | 0.067846334 | 0.727668599 | 1.070200842 | 0.730505179 | 1.56785999 |
| ENSG00000213928 | 0.202780211 | 0.26432091 | 1.224803241 | 0.857893704 | 1.748635024 |
| ENSG00000168610 | -0.159553314 | 0.471168058 | 0.852524515 | 0.552370974 | 1.315778858 |
| ENSG00000166278 | -0.033569111 | 0.726943246 | 0.96698808 | 0.80092636 | 1.167480548 |
| ENSG00000147168 | -0.087511939 | 0.339399917 | 0.916207933 | 0.765636173 | 1.09639148 |
| ENSG00000105127 | -0.050117573 | 0.87407217 | 0.951117592 | 0.511758206 | 1.767679859 |
| ENSG00000198736 | -0.118118396 | 0.358716393 | 0.888590841 | 0.690487973 | 1.143529959 |
| ENSG00000197329 | 0.21511033 | 0.248031355 | 1.239998699 | 0.860817218 | 1.786205876 |
| ENSG00000107679 | -0.176498228 | 0.432300143 | 0.838200264 | 0.539546267 | 1.302167629 |
| ENSG00000126709 | 0.029144964 | 0.645831471 | 1.029573835 | 0.909232619 | 1.165842777 |
| ENSG00000131467 | -0.318860662 | 0.152437302 | 0.726976838 | 0.469731247 | 1.125101483 |
| ENSG00000185344 | -0.366892863 | 0.24545031 | 0.692883875 | 0.373059017 | 1.28689575 |
| ENSG00000131019 | 0.085135626 | 0.634237819 | 1.088864736 | 0.766756944 | 1.546287153 |
| ENSG00000154589 | 0.07286422 | 0.46919697 | 1.075584484 | 0.882984939 | 1.310194467 |
| ENSG00000122861 | -0.114376162 | 0.225864591 | 0.891922386 | 0.74120506 | 1.073286712 |
| ENSG00000139278 | -0.15797441 | 0.191813453 | 0.853871633 | 0.673550768 | 1.082467425 |
| ENSG00000197594 | 0.014507702 | 0.929580973 | 1.014613449 | 0.735465171 | 1.399713395 |
| ENSG00000114013 | -0.008413879 | 0.946225878 | 0.991621418 | 0.776533416 | 1.266285542 |
| ENSG00000146072 | 0.14290658 | 0.470760794 | 1.153622025 | 0.78236024 | 1.701062642 |
| ENSG00000197405 | 0.108619454 | 0.294872868 | 1.11473806 | 0.909719794 | 1.365960102 |
| ENSG00000116525 | 0.238903695 | 0.403418585 | 1.269856237 | 0.725053892 | 2.224020698 |
| ENSG00000079739 | 0.02950684 | 0.812413114 | 1.02994648 | 0.807196212 | 1.31416592 |
| ENSG00000038002 | -0.102610291 | 0.585160946 | 0.902478609 | 0.624351719 | 1.304501318 |
| ENSG00000090376 | -0.212923123 | 0.264080916 | 0.808218268 | 0.55621508 | 1.174396005 |
| ENSG00000054219 | -0.020589948 | 0.89217589 | 0.979620577 | 0.727381703 | 1.319329963 |
| ENSG00000170017 | -0.047829544 | 0.681810461 | 0.953296268 | 0.758450817 | 1.198197372 |
| ENSG00000128342 | 0.012796385 | 0.912101238 | 1.012878609 | 0.807021298 | 1.271246596 |
| ENSG00000104365 | -0.025695662 | 0.902464975 | 0.974631662 | 0.646192765 | 1.470005434 |
| ENSG00000164850 | 0.162562384 | 0.24087894 | 1.176521712 | 0.896630999 | 1.543782605 |
| ENSG00000254996 | -0.721506162 | 0.173483663 | 0.48601968 | 0.171964327 | 1.37362867 |
| ENSG00000188811 | -0.130715822 | 0.381519361 | 0.877467096 | 0.654764047 | 1.175917503 |
| ENSG00000171314 | 0.093844258 | 0.600446958 | 1.098388668 | 0.773111444 | 1.560522322 |
| ENSG00000142089 | -0.000206455 | 0.998246918 | 0.999793567 | 0.831625807 | 1.20196748 |
| ENSG00000102970 | -0.296989981 | 0.11312693 | 0.743051457 | 0.514580775 | 1.072961709 |
| ENSG00000138413 | -0.178978623 | 0.320231658 | 0.836123773 | 0.587491123 | 1.189980473 |
| ENSG00000050730 | -0.434890188 | 0.111015716 | 0.647335749 | 0.379179536 | 1.105132348 |
| ENSG00000101082 | 0.063635258 | 0.755842507 | 1.065703621 | 0.71357068 | 1.591607165 |
| ENSG00000153310 | -0.153933544 | 0.341437781 | 0.857328994 | 0.624331683 | 1.17727968 |
| ENSG00000111981 | 0.17065296 | 0.56648693 | 1.186079061 | 0.661772515 | 2.125781151 |
| ENSG00000103035 | -0.00154875 | 0.994147625 | 0.998452449 | 0.660083074 | 1.510275498 |
| ENSG00000143502 | 0.902122268 | 0.017399384 | 2.464828592 | 1.171911501 | 5.184162784 |
| ENSG00000117335 | -0.19609245 | 0.125712834 | 0.821936243 | 0.639486586 | 1.056439968 |
| ENSG00000120885 | 0.151172915 | 0.026188191 | 1.163197775 | 1.018073443 | 1.32900929 |
| ENSG00000111229 | -0.118415157 | 0.46447204 | 0.88832718 | 0.646816458 | 1.22001407 |
| ENSG00000242574 | 0.016093517 | 0.860362159 | 1.016223715 | 0.849408625 | 1.215799566 |
| ENSG00000072736 | -0.171908827 | 0.458244324 | 0.842055942 | 0.534642018 | 1.326229863 |
| ENSG00000117971 | 1.444117552 | 0.002304267 | 4.238110578 | 1.67444208 | 10.72690509 |
| ENSG00000198707 | -0.010954374 | 0.954294338 | 0.989105407 | 0.680073389 | 1.438564603 |
| ENSG00000086758 | 0.092326069 | 0.567085267 | 1.096722371 | 0.79944541 | 1.504542953 |
| ENSG00000107874 | 0.151847399 | 0.399158811 | 1.163982598 | 0.81779492 | 1.656717908 |
| ENSG00000275385 | -0.038159896 | 0.464781151 | 0.962559019 | 0.868945369 | 1.066257901 |
| ENSG00000076248 | -0.041823214 | 0.813950569 | 0.959039311 | 0.676957243 | 1.358662469 |
| ENSG00000113749 | -0.093842947 | 0.776323172 | 0.910425736 | 0.476532121 | 1.73938961 |
| ENSG00000109971 | -0.217983369 | 0.154413645 | 0.804138815 | 0.595718374 | 1.085478077 |
| ENSG00000104763 | -0.471778175 | 0.004292393 | 0.623891893 | 0.451328256 | 0.862434577 |
| ENSG00000099985 | -0.00548599 | 0.951007381 | 0.994529031 | 0.834863952 | 1.184729549 |
| ENSG00000177105 | 0.293665564 | 0.100555538 | 1.341335238 | 0.944753562 | 1.904391042 |
| ENSG00000135341 | 0.070102477 | 0.754492051 | 1.072618094 | 0.691245405 | 1.664401046 |
| ENSG00000240065 | -0.053272611 | 0.564370268 | 0.948121509 | 0.791021473 | 1.136422242 |
| ENSG00000018280 | 0.128111693 | 0.308720717 | 1.136679955 | 0.888194408 | 1.454683016 |
| ENSG00000128604 | 0.136906987 | 0.406525428 | 1.146721484 | 0.829961222 | 1.584375422 |
| ENSG00000204252 | 0.079406573 | 0.352699247 | 1.082644407 | 0.915709656 | 1.280011523 |
| ENSG00000092010 | -0.03448156 | 0.805306644 | 0.966106155 | 0.734425422 | 1.270872541 |
| ENSG00000147533 | -0.233058083 | 0.172166454 | 0.792107565 | 0.566866251 | 1.106847325 |
| ENSG00000164733 | -0.141783069 | 0.315603434 | 0.867809491 | 0.657905311 | 1.144683436 |
| ENSG00000156738 | -0.016249825 | 0.904167494 | 0.983881491 | 0.755194207 | 1.281819669 |
| ENSG00000169692 | 0.179673414 | 0.12290673 | 1.196826432 | 0.952562705 | 1.503726213 |
| ENSG00000158473 | 0.135386169 | 0.41771462 | 1.144978855 | 0.825265405 | 1.588551477 |
| ENSG00000131504 | -0.492557402 | 0.031922583 | 0.611061664 | 0.389634774 | 0.958324004 |
| ENSG00000198168 | 0.025834824 | 0.880977136 | 1.026171435 | 0.731731758 | 1.439089945 |
| ENSG00000054148 | 0.090843271 | 0.480376273 | 1.095097359 | 0.850900867 | 1.409374784 |
| ENSG00000177556 | -0.231826986 | 0.322400102 | 0.793083326 | 0.501072969 | 1.255268596 |
| ENSG00000176978 | 0.364675238 | 0.003998175 | 1.440046259 | 1.123391178 | 1.845958264 |
| ENSG00000121691 | 0.008858071 | 0.954650661 | 1.00889742 | 0.743461148 | 1.36910181 |
| ENSG00000262406 | -0.109219762 | 0.05120189 | 0.896533372 | 0.803313863 | 1.000570418 |
| ENSG00000224389 | 0.250656102 | 0.14186873 | 1.284868144 | 0.919611885 | 1.795198795 |
| ENSG00000026297 | 0.076984502 | 0.652057152 | 1.080025338 | 0.772868981 | 1.509252875 |
| ENSG00000141433 | 0.487553776 | 0.052745682 | 1.628328088 | 0.994235809 | 2.666824449 |
| ENSG00000145113 | -0.046874559 | 0.527730744 | 0.954207086 | 0.825006941 | 1.10364061 |
| ENSG00000173391 | -0.001479065 | 0.987295249 | 0.998522029 | 0.832327643 | 1.197901151 |
| ENSG00000254999 | 0.07352471 | 0.760929984 | 1.076295131 | 0.670251183 | 1.728324006 |
| ENSG00000198931 | 0.134009671 | 0.323540603 | 1.143403877 | 0.876299087 | 1.491924898 |
| ENSG00000157368 | 0.195359426 | 0.133524889 | 1.215747879 | 0.941908894 | 1.569199436 |
| ENSG00000132510 | 0.237476265 | 0.197815458 | 1.268044899 | 0.883423981 | 1.820120236 |
| ENSG00000100985 | -0.053215858 | 0.399982472 | 0.94817532 | 0.837662409 | 1.073268213 |
| ENSG00000179588 | 0.093977576 | 0.512509967 | 1.098535113 | 0.829227487 | 1.455305586 |
| ENSG00000108424 | -0.207428422 | 0.284498734 | 0.812671409 | 0.555832286 | 1.188190818 |
| ENSG00000122122 | -0.013878683 | 0.896492624 | 0.986217182 | 0.800136739 | 1.215572642 |
| ENSG00000148334 | 0.037142901 | 0.781055484 | 1.037841318 | 0.798695086 | 1.348593 |
| ENSG00000170266 | -0.28179421 | 0.149614818 | 0.754428922 | 0.514220767 | 1.106845611 |
| ENSG00000177455 | 0.178974113 | 0.263842722 | 1.195989784 | 0.873745326 | 1.637080646 |
| ENSG00000198918 | 0.04843 | 0.563847931 | 1.049621895 | 0.890440251 | 1.23726002 |
| ENSG00000058600 | -0.009511146 | 0.968853665 | 0.990533942 | 0.614507242 | 1.596657325 |
| ENSG00000241685 | 0.092983709 | 0.698020261 | 1.097443857 | 0.686103975 | 1.755394317 |
| ENSG00000151694 | -0.057652601 | 0.793736334 | 0.943977828 | 0.612732657 | 1.454295163 |
| ENSG00000143507 | 0.03375998 | 0.802678169 | 1.034336315 | 0.793709473 | 1.347913374 |
| ENSG00000138071 | -0.050506311 | 0.750587255 | 0.950747928 | 0.696331496 | 1.298119687 |
| ENSG00000142166 | -0.034798018 | 0.867201289 | 0.965800471 | 0.642316222 | 1.452198337 |
| ENSG00000162944 | 0.30610577 | 0.277402631 | 1.358125948 | 0.781724916 | 2.359533455 |
| ENSG00000204516 | 0.024574397 | 0.844971054 | 1.024878836 | 0.801120038 | 1.311135136 |
| ENSG00000142192 | 0.05216536 | 0.789423351 | 1.053549943 | 0.71843574 | 1.544978097 |
| ENSG00000216490 | 0.545149281 | 0.048950817 | 1.724865852 | 1.00251037 | 2.967712152 |
| ENSG00000064666 | 0.047304642 | 0.768511124 | 1.048441359 | 0.765131326 | 1.436654397 |
| ENSG00000102962 | -0.42103201 | 0.00333566 | 0.656369091 | 0.495496663 | 0.869471815 |
| ENSG00000198286 | 0.050347311 | 0.530630233 | 1.051636278 | 0.898503905 | 1.230867062 |
| ENSG00000143669 | -0.259121683 | 0.27646222 | 0.771729111 | 0.483944977 | 1.2306478 |
| ENSG00000196396 | 0.178497037 | 0.43143327 | 1.195419341 | 0.766299297 | 1.864842375 |
| ENSG00000197943 | 0.382865886 | 0.02329544 | 1.466481341 | 1.053461136 | 2.041430337 |
| ENSG00000085365 | -0.149430057 | 0.319860345 | 0.861198671 | 0.641558559 | 1.156033444 |
| ENSG00000068745 | -0.086140451 | 0.717499108 | 0.917465363 | 0.575347316 | 1.463016631 |
| ENSG00000237541 | 0.013517542 | 0.837405953 | 1.013609317 | 0.890843044 | 1.153293899 |
| ENSG00000135828 | -0.001454394 | 0.994100034 | 0.998546663 | 0.679126684 | 1.468202416 |
| ENSG00000136244 | 0.010554367 | 0.912663234 | 1.010610261 | 0.836900042 | 1.220376447 |
| ENSG00000121858 | -0.010094673 | 0.937058476 | 0.989956107 | 0.770555744 | 1.271826343 |
| ENSG00000173369 | 0.106008666 | 0.130083857 | 1.111831511 | 0.969236475 | 1.275405272 |
| ENSG00000125347 | -0.05352991 | 0.657159167 | 0.947877589 | 0.748327455 | 1.200640065 |
| ENSG00000154124 | -0.007742618 | 0.972770958 | 0.992287279 | 0.636142818 | 1.547819165 |
| ENSG00000101439 | 0.051939213 | 0.61670671 | 1.053311713 | 0.859464352 | 1.290880257 |
| ENSG00000249437 | -1.029958345 | 0.29286428 | 0.357021832 | 0.052386087 | 2.433176379 |
| ENSG00000170425 | 0.004842492 | 0.969215639 | 1.004854236 | 0.785769458 | 1.285023267 |
| ENSG00000106178 | -0.125237994 | 0.032007283 | 0.882286898 | 0.786856975 | 0.989290551 |
| ENSG00000107338 | 0.008737412 | 0.958413547 | 1.008775695 | 0.726383561 | 1.400951861 |
| ENSG00000013725 | 0.148037171 | 0.403991952 | 1.159555997 | 0.819019271 | 1.641683117 |
| ENSG00000165030 | -0.018704301 | 0.922720256 | 0.981469539 | 0.672596411 | 1.432184949 |
| ENSG00000132824 | 0.110481697 | 0.50830856 | 1.116815907 | 0.805027801 | 1.549359872 |
| ENSG00000067182 | 0.270362372 | 0.179815044 | 1.310439231 | 0.882765915 | 1.9453073 |
| ENSG00000051620 | -0.364810926 | 0.120415487 | 0.694327918 | 0.438142472 | 1.100307065 |
| ENSG00000100604 | 0.047538505 | 0.323991058 | 1.04868658 | 0.954153741 | 1.152585265 |
| ENSG00000075624 | 0.15412421 | 0.44490702 | 1.166635786 | 0.785605706 | 1.73247094 |
| ENSG00000144589 | 0.133863816 | 0.584189204 | 1.143237118 | 0.707835293 | 1.846462195 |
| ENSG00000152495 | 0.132092457 | 0.748235537 | 1.141213827 | 0.509399885 | 2.556673133 |
| ENSG00000184381 | 0.282086969 | 0.178192935 | 1.325894026 | 0.879354323 | 1.999188406 |
| ENSG00000163362 | 0.07087583 | 0.619094164 | 1.073447927 | 0.811757447 | 1.419500931 |
| ENSG00000273749 | 0.000794926 | 0.997361008 | 1.000795242 | 0.624836673 | 1.602964679 |
| ENSG00000117280 | 0.03239756 | 0.832891915 | 1.032928075 | 0.764487533 | 1.395628263 |
| ENSG00000138814 | -0.109241416 | 0.54593509 | 0.896513958 | 0.628884505 | 1.278036383 |
| ENSG00000160883 | 0.051219645 | 0.65779952 | 1.052554056 | 0.839108534 | 1.320294092 |
| ENSG00000162897 | -0.005025634 | 0.978691048 | 0.994986973 | 0.688111835 | 1.438718282 |
| ENSG00000131979 | -0.157328774 | 0.382415317 | 0.854423101 | 0.600278088 | 1.216167721 |
| ENSG00000184216 | 0.168388078 | 0.203764871 | 1.183395771 | 0.91274567 | 1.534299857 |
| ENSG00000153029 | 0.231814714 | 0.227188742 | 1.260886082 | 0.865527513 | 1.836837869 |
| ENSG00000124570 | 0.10202689 | 0.413484976 | 1.10741325 | 0.867186756 | 1.414186848 |
| ENSG00000026508 | -0.07850998 | 0.533678602 | 0.924492833 | 0.721992111 | 1.183789942 |
| ENSG00000129657 | 0.090482972 | 0.650339423 | 1.094702867 | 0.740261026 | 1.618853788 |
| ENSG00000114520 | -0.135039692 | 0.457657123 | 0.873681233 | 0.611768923 | 1.247724211 |
| ENSG00000055917 | 0.08361112 | 0.670702941 | 1.087206019 | 0.739478381 | 1.598446903 |
| ENSG00000110777 | -0.021614632 | 0.85108678 | 0.97861729 | 0.78092492 | 1.226355795 |
| ENSG00000095970 | 0.08117295 | 0.323472687 | 1.084558455 | 0.923151433 | 1.274186444 |
| ENSG00000171049 | -0.226412611 | 0.224568809 | 0.797389023 | 0.55332628 | 1.14910366 |
| ENSG00000204498 | 0.441859373 | 0.029822973 | 1.555596966 | 1.044162352 | 2.31753416 |
| ENSG00000221983 | 0.027298492 | 0.852479948 | 1.027674509 | 0.770722296 | 1.37029239 |
| ENSG00000121236 | 0.803880574 | 0.027393163 | 2.234194084 | 1.093752818 | 4.563758029 |
| ENSG00000073861 | 0.284228911 | 0.346699195 | 1.328737058 | 0.735083657 | 2.401824817 |
| ENSG00000156234 | -0.098988069 | 0.172029205 | 0.905753515 | 0.785803896 | 1.044012932 |
| ENSG00000002586 | 0.064344251 | 0.673887665 | 1.066459465 | 0.790302561 | 1.439114394 |
| ENSG00000077984 | 0.042899419 | 0.627832842 | 1.0438329 | 0.877618745 | 1.24152672 |
| ENSG00000231925 | 0.13024899 | 0.422069499 | 1.139111975 | 0.828840186 | 1.565532312 |
| ENSG00000109332 | -0.359416627 | 0.117184786 | 0.698083451 | 0.445280043 | 1.09441353 |
| ENSG00000276045 | 0.160448633 | 0.326249557 | 1.174037465 | 0.852238975 | 1.617344442 |
| ENSG00000150637 | -0.469641191 | 0.401856273 | 0.625226565 | 0.208532829 | 1.874564594 |
| ENSG00000159352 | 0.270560689 | 0.185273458 | 1.310699139 | 0.878314322 | 1.955942412 |
| ENSG00000196549 | -0.151847119 | 0.136913279 | 0.859119613 | 0.703322401 | 1.049428412 |
| ENSG00000049249 | -0.39333411 | 0.142855823 | 0.674803251 | 0.398728663 | 1.142028326 |
| ENSG00000275718 | -0.128839692 | 0.323752055 | 0.879114884 | 0.680624362 | 1.135491207 |
| ENSG00000081059 | -0.159945659 | 0.187794841 | 0.852190097 | 0.671693102 | 1.081190144 |
| ENSG00000143947 | 0.134636324 | 0.443087267 | 1.144120619 | 0.811060365 | 1.613951376 |
| ENSG00000113263 | -0.028490608 | 0.914421614 | 0.971911422 | 0.578037696 | 1.634169916 |
| ENSG00000205339 | -0.012557649 | 0.935599694 | 0.987520869 | 0.72821273 | 1.339165641 |
| ENSG00000141655 | -0.193983621 | 0.087332153 | 0.823671395 | 0.659436277 | 1.028809895 |
| ENSG00000111144 | -0.196320794 | 0.395942746 | 0.82174858 | 0.522256053 | 1.292987846 |
| ENSG00000171150 | 0.037167896 | 0.857668458 | 1.03786726 | 0.691408422 | 1.557933655 |
| ENSG00000110330 | -0.212906865 | 0.200913262 | 0.808231409 | 0.583225636 | 1.120043376 |
| ENSG00000071889 | 0.21488423 | 0.12790611 | 1.239718367 | 0.940109035 | 1.634812103 |
| ENSG00000171867 | 0.267470828 | 0.019661306 | 1.306655511 | 1.043669861 | 1.635908718 |
| ENSG00000103241 | 0.018268935 | 0.872164789 | 1.018436833 | 0.815253086 | 1.272259621 |
| ENSG00000173930 | -0.030817103 | 0.925521922 | 0.969652904 | 0.508166428 | 1.850233903 |
| ENSG00000009694 | 0.523758216 | 0.388194406 | 1.688360967 | 0.513813631 | 5.547853501 |
